# Benchmark Study for Calculations of p*K*_a_ Values of Metal Ligands in Proteins

**DOI:** 10.1021/acs.jctc.6c00884

**Published:** 2026-07-06

**Authors:** Maryam Haji Dehabadi, Mehdi Irani, Sonia Jafari, Ulf Ryde

**Affiliations:** † Department of Chemistry, 48530 University of Kurdistan, Sanandaj 66177-15175, Iran; ‡ Division of Computational Chemistry, Lund University, Chemical Centre, P.O. Box 124, SE-221 00 Lund, Sweden

## Abstract

We have compared the performance of 64 different computational methods, based on combined quantum mechanical (QM) and molecular mechanical (QM/MM) or QM-cluster calculations in a continuum solvent, to estimate the acid constant (p*K*_a_) of metal-bound ligands in proteins. As a calibration set, we use 12 experimental p*K*_a_ values from six different proteins that involve Zn^2+^, Fe^3+^, or Fe^4+^. We employ two different density functional theory (DFT) methods (TPSS and B3LYP), two basis sets (def2-SV­(P) and def2-TZVPD), QM regions of three different sizes (∼40, ∼100, and ∼350 atoms), relaxed or fixed surroundings, and three different values of the dielectric constant of the continuum-solvation model (ε = 4, 20, or 80). The results clearly show that QM-cluster+continuum-solvation is much better than QM/MM. In general, the most accurate results are obtained with ε = 80 and the minimal QM region. The two DFT methods, the two basis sets, and relaxing or fixing the surroundings give similar results. The best-performing method is TPSS with the minimal QM region, def2-TZVPD, relaxed surroundings, and ε = 80, yielding a mean absolute deviation (after removal of a systematic error of 11.6 p*K*_a_ units, pu) of 2.0 pu and a maximum deviation of 5.0 pu. The coefficient of determination (*R* ^2^) and Kendall’s τ with respect to the experimental p*K*_a_ values are both 0.64, while Spearman’s rank correlation coefficient is 0.78. This level of accuracy should be sufficient to reliably determine the protonation states of metal-bound ligands in QM-based studies of enzymatic reaction mechanisms.

## Graphical abstract

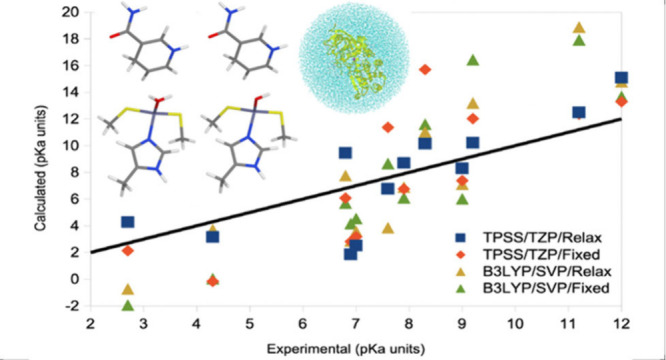

## 1 Introduction

Metalloenzymes are a broad group of enzymes that contain metal ions in their active sites. Metal ions strongly distort the properties of bound ligands, in particular their p*K*_a_ values. p*K*_a_ values can have a significant impact on the structure and function of metalloenzymes, and therefore on their reaction mechanisms. Unfortunately, it is quite hard to deduce the protonation state of metal ligands by experiments, especially as protons are not discernible in X-ray crystal structures. Therefore, computational methods are often employed for predicting p*K*_a_ values in proteins.^1^ However, such calculations are challenging due to the complexity and diversity of the systems involved, as well as the fact that protonation reactions typically involve a change in the net charge of the studied system, resulting in large and long-range solvation effects and electrostatic interactions. Several approaches have been used for computational p*K*_a_ calculations: microscopic methods,^2,3^ macroscopic methods based on continuum electrostatics,^4^ and knowledge-based methods that rely on empirical parameters.^5,6^

Among the three classes of computational p*K*_a_ calculations, microscopic methods such as quantum mechanical (QM) or quantum mechanics/molecular mechanics (QM/MM) approaches are considered the most reliable for computing p*K*_a_ values of small molecules.^3,7^ Traditional QM methods use thermodynamic cycles to compute protonation/deprotonation free energies in both gas-phase and solution,^8–17^ yet they may not always yield reliable p*K*_a_ values due to factors such as species instability in the gas-phase or significant conformational differences between gas-phase and solution.^14,18^ In the case of proteins, QM approaches are impractical due to system size and can only be applied to model systems that capture the local protein environment of the residue of interest. Nevertheless, the choice of the model size and local environment can influence theoretical p*K*_a_ values.^19^ A more practical approach is the hybrid QM/MM method, in which the titratable residue and its immediate surroundings are modeled at the QM level, while the remaining environment is treated with an MM force field.^20–22^ Molecular dynamics (MD) based methods, such as free energy perturbation^23,24^ and constant pH MD (CPHMD) simulations,^25–35^ can provide reliable p*K*_a_ values for protein residues. Combining enhanced sampling techniques with CPHMD simulations further improves the accuracy of p*K*_a_ predictions.^31,36–41^

Macroscopic methods rely on either the numerical solution of the Poisson–Boltzmann equation (PBE)^4,42–45^ or the generalized Born (GB) technique with analytical approximations of electrostatic energies.^46,47^ These methods model proteins as a homogeneous medium with a low dielectric constant, while the environment (solvent) is modeled with a high dielectric constant. The PBE-based methods and their variations^45,48–53^ allow modeling of the solvent accessibility to titratable residues^54,55^ and multiple ionizable residues.^56,57^ Even though there are different suggestions for the dielectric constant of proteins, ranging from 4 to 80,^58–66^ the appropriate value depends on the polarity of the surrounding residues and the flexibility of the protein.^66,67^ This issue can be addressed by accounting for the protein flexibility through techniques that use ensembles of conformers.^68–75^ An example of such an approach is the multiconformation continuum electrostatic method, which has been shown to successfully predict p*K*_a_ values of several protein residues with different force fields.^61,68,76,77^

Empirical methods are based on the statistical fitting of environmental descriptors and parameters to the three-dimensional structures of proteins. The reasonably accurate predictions and the low computational cost make them widespread and attractive. There are a variety of empirical tools with comparable accuracies,^78–80^ but PROPKA^5,6,21,81^ is a widely used empirical tool that estimates changes in amino acid p*K*_a_ values from water to a protein environment. In this tool, the environmental perturbation is expressed as the sum of contributions from the protein environment. Recent studies with machine-learning algorithms for p*K*_a_ estimations of transition metal–hydride complexes have provided results with a root-mean-squared error of ∼2.7 p*K*_a_ units (pu).^82^

Numerous theoretical approaches have been used to estimate the p*K*_a_ values of metal-bound ligands in proteins. For example, Pettersson and Eklund used a simple point-charge model with a screened dielectric constant to estimate the effects of electrostatic interactions between bound NADH and NAD and ionizing Zn-bound groups in liver alcohol dehydrogenase.^83^ The results provided clear evidence that the effects of coenzyme binding on the kinetic constants are predominantly of electrostatic origin and attributable to ionization of the water molecule that is bound to the active-site zinc ion.

Merz has presented an approach to estimate the p*K*_a_ of ionizable groups in human carbonic anhydrase II (CAII) using free-energy perturbation simulations.^84^ The activity of CAII is dependent on a group with a p*K*_a_ around 7.0. They reported that Glu-106 had a p*K*_a_ of 2.2 ± 2.8, which suggested that this residue could not be the activity-linked group.

Riccardi et al. explored the applicability of the generalized solvent boundary potential (GSBP) based QM/MM approach to study chemical reactions in biomolecules. They analyzed structural and energetic properties of human CAII and compared them to those from MD simulations and available experimental data.^85^ The p*K*_a_ of the zinc-bound water calculated with a semiempirical QM thermodynamic integration approach agreed well with experiments for the wild-type CAII. For the E106Q mutant, a 9 pu downward shift relative to the wild-type enzyme was found, in contrast to experiments that found little change. In a review of their QM/MM studies on CAII,^86^ they highlighted the importance of electrostatics in shaping the energetics and kinetics of proton transfer in CAII for its function. Once the p*K*_a_ for the zinc-water is in the proper range (near 7.0), proton transfer through a relatively well-solvated cavity toward the protein surface is facile, making this process secondary to the modulation of p*K*_a_ of the zinc-bound water.

In another study, the p*K*_a_ of the zinc-bound water in CAII was determined using empirical valence-bond simulations.^87^ They obtained a p*K*_a_ of 7.1 ± 0.3, in good agreement with the experimental value of 6.9 ± 0.1.^88^ Jiao et al. investigated the p*K*_a_ shifts of zinc-bound water due to mutations to the active site of CAII using a density functional theory (DFT)/continuum technique.^89^ Their calculations supported the conclusion that changes in conformation and electronic polarization in mutated active sites account for the altered deprotonation behavior of the zinc-bound water.

Thus, various computational methods have been used to estimate the p*K*_a_ values of metal-bound ligands in proteins, with varying results. However, we are not aware of any study that has compared multiple methods across a range of proteins. In this study, we calculate the p*K*_a_ values for metal-bound ligands in six metalloproteins, each featuring unique combinations of metal centers (Zn or Fe), ligands, cofactors, and mutations. The proteins examined include alcohol dehydrogenase (ADH) in various states (with NAD^+^, NADH, or no coenzyme), thioredoxin-like ferredoxins (TLF) in two mutant forms (C55S and C59S), carbonic anhydrase (CA), both wild-type and E106Q mutant, compound II (CmpII) from myoglobin (Mb) and cytochrome P450, as well as the heme nitric oxide/oxygen binding domain (H-NOX), in both wild-type and I5L mutant forms. A more detailed description of the studied systems is given in the Supporting Information and in Table 1. We compare the results of 64 different approaches involving either QM/MM^90–98^ or QM-cluster calculations in a continuum solvent,^99^ and test various methodological parameters (e.g., DFT functional, basis set, QM region size, dielectric constant, and treatment of surrounding residues).

**Table 1:** Studied Systems, Describing the Protein, the Organism, the Crystal Structure (Protein Data Bank Code; PDB) Used for the Calculations, the Coenzyme or the Mutated Form of the Protein (State Column), the Metal Center, the Protonable Ligand in the Acid (AH) and Base (A^–^) Forms, the Experimental p*K*_a_ Value, and the Abbreviation We Use in This Article

Protein	Organism	PDB ID	State	Metal Ion	AH	A^–^	p*K*_a_	Abbreviation
ADH	*Equus caballus*	1AXE^107^	NAD^+^	Zn(II)	CF_3_CH_2_OH	CF_3_CH_2_O^–^	4.3 ^108^	ADH-TFE
		1AXE^107^	NAD^+^	Zn(II)	HOH	OH^–^	7.6 ^109–111^	ADH-NAD
		1AXE^107^	NADH				11.2 ^112^	ADH-NADH
		8ADH^100^	No coenzyme				9.2 ^109^	ADH-Apo
TLF	*Aquifex aeolicus*	1M2B^101^	C55S	Fe(III)_2_	Ser	Ser^–^	9.0 ^113^	TLF-C55S
		1M2D^101^	C59S	Fe(III)_2_			8.3 ^113^	TLF-C59S
CA	*Homo sapiens*	2ILI^102^	WT	Zn(II)	HOH	OH^–^	∼7.0 ^114^	CA-WT
		2ILI^102^	E106Q	Zn(II)			∼6.9 ^115^	CA-E106Q
Mb	*Equus caballus*	2V1E^103^	CmpII	Fe(IV)	OH^–^	O^2–^	<2.7 ^116^	Mb-CmpII
P450	*Streptomyces coelicolor*	1S1F^104^	CmpII	Fe(IV)	OH^–^	O^2–^	≈12 ^117^	P450-CmpII
H-NOX	*Thermoanaerobacter tengcongensis*	1U56 ^106^	WT	Fe(III)	HOH	OH^–^	6.8 ^105^	HNOX-WT
		3NVR^105^	I5L	Fe(III)	HOH	OH^–^	7.9 ^105^	HNOX-I5L

## 2 Methods

### 2.1 Studied Systems

We have studied six different metalloproteins. The systems, the experimental p*K*_a_ values, and the employed crystal structures are described in Table 1. Four states of ADH from horse were studied: three with reduced or oxidized NAD coenzyme (1AXE) and the last without the coenzyme (8ADH).^100^ Two mutant [2Fe–2S] ferredoxins from *Aquifex aeolicus* were studied: 1M2B^101^ and 1M2D^101^ for the C55S and C59S mutants, respectively. For CA, structures from *Homo sapiens* (2ILI)^102^ were used for the wild-type and the E106Q mutant. Mb and P450 were studied from horse (2V1E)^103^ and *Streptomyces coelicolor* (1S1F),^104^ respectively. H-NOX structures from *Thermoanaerobacter tengcongensis* were chosen for the wild-type (1U56)^105^ and the I5L mutant (3NVR).^106^ Altogether, these choices resulted in 12 distinct systems, as summarized in Table 1.

Each protein was set up starting from the crystal structure specified in Table 1. For each structure, all heteromolecules were removed, except those in the active site, the metal, and its ligands. For ADH, we also kept the NAD coenzyme. For all systems, we used only the first chain of dimeric protein structures. For residues with alternative conformations, we kept the one with the highest occupation or the first if they have equal occupation numbers. All crystal-water molecules were kept. The protonation states of all titrable protein residues were determined by a detailed analysis of their local environments. All Asp, Glu, Arg, and Lys residues were assumed to be charged unless they are buried within the protein and the hydrogen-bond pattern suggests they are neutral. We used PROPKA^5,6,21,81^ to identify residues that may have unusual protonation states (estimated p*K*_a_ values above 7 for Asp, Glu, and His or below 7 for Lys, Arg, Cys, and Tyr). This list was extended by identifying all titrable groups that are not solvent-accessible and do not form any ionic pairs (possibly water-mediated) using the local software *changepdb* (version 2408). Each residue on this list was then visually investigated for solvent accessibility and the formation of hydrogen bonds. Based on this study, Asp-49 and Glu-267 in the ADH structure, Glu-106 for the wild type of CA, and Glu-362 in the P450 structure were neutralized. A thorough manual investigation of all His residues gave the protonation assignment detailed in Table S1 in the Supporting Information. All Cys residues were protonated, except those coordinated to the metal ions. There are no disulfide bridges in any of the studied proteins.

### 2.2 QM/MM Calculations

After protonation, the proteins were immersed in a periodic truncated octahedral box of TIP3P water molecules,^118^ extending at least 20 Å from the solute, using the *tleap* module in the Amber software suite.^119^ Next, hydrogen atoms and added water molecules were subjected to 1000 cycles of minimization with the heavy atoms of the proteins restrained to the starting crystal structures with a force constant of 1000 kcal/mol/Å^2^. This was followed by a 10 ps constant-volume equilibration with the same restraints. Finally, the systems were equilibrated through a 1 ns constant-volume simulation and a 1 ns simulated annealing at constant pressure, with the same restraints. Bond lengths involving hydrogen atoms were constrained by the SHAKE algorithm^120^ (not during minimizations), allowing a time step of 2 fs during the simulations. The temperature was maintained at 300 K using Langevin dynamics with a collision frequency of 2 ps^–1^.^121^ The pressure was kept constant at 1 atm using Berendsen’s weak coupling isotropic algorithm with a relaxation time of 1 ps.^122^ Long-range electrostatics were handled by particle-mesh Ewald summation^123^ with a fourth-order B-spline interpolation and a tolerance of 10^–5^. The cutoff radius for the Lennard-Jones interactions was set to 8 Å. After the final equilibration, the octahedral systems were truncated to a spherical shape with the largest radius that fits into the octahedral system around the geometric center of the proteins.

Based on these equilibrated structures, we performed QM/MM calculations^97,98^ using the ComQum program package (version 3.0).^124,125^ In this approach, the protein and solvent are split into three subsystems: System 1 (the QM region) was relaxed by QM methods, whereas System 2 contained all atoms in residues and water molecules with at least one atom within 6 Å of any atom in the QM region and was optionally optimized by MM in each step of the QM/MM geometry optimization. System 3 involved the remaining part of the protein and the solvent, and was kept fixed at the original coordinates (equilibrated crystal structure).

In the QM calculations, system 1 was represented by a wave function, whereas the other atoms were represented by an array of partial point charges, one per atom, taken from MM libraries. Thereby, the polarization of the QM system by the surroundings is included in a self-consistent manner. The QM calculations were performed using the Turbomole software.^126,127^ Two DFT methods (TPSS and B3LYP)^10,128–130^ and two different basis sets (def2-SV­(P) and def2-TZVPD)^131^ were used. For ADH, TLF, and H-NOX with the largest QM region, calculations with the larger basis set suffered from severe convergence problems that could not be resolved despite extensive attempts using various strategies. For these cases, the def2-TZVP basis set was used instead.^131^ The calculations were accelerated by the resolution-of-identity approximation,^132–134^ which involves fitting the electron density in an auxiliary basis set (we employed the built-in def2-SV­(P)^135^ or universal^136^ auxiliary basis sets). Empirical dispersion corrections were included using the DFT-D3 approach^137^ and Becke–Johnson (BJ) damping,^138^ as implemented in Turbomole.

The MM part of the QM/MM calculations was performed using the Amber software,^119^ with the Amber ff14SB^139^ force field for the protein and the GAFF^140^ force field for the substrate. Water molecules were described by the TIP3P model.^118^

When a bond exists between systems 1 and 2 (a junction), the hydrogen link-atom approach was employed. In this approach, the QM system is capped with hydrogen link atoms (HL), whose positions are linearly related to the corresponding heavy atoms (carbon link atoms, CL) in the full system.^124,141^ All atoms were included in the point-charge model, except the CL atoms.^142^ The point charges do not necessarily sum up to an integer, because the Amber force field does not employ charge groups.^139^

The total QM/MM energy in ComQum is calculated by eq 1,^124,125^

1 $$E_{\mathrm{QM}/\mathrm{MM}}=E_{\mathrm{QM}1+\mathrm{ptch}23}^{\mathrm{HL}}+E_{\mathrm{MM}123,q_{1}=0}^{\mathrm{CL}}-E_{\mathrm{MM}1,q_{1}=0}^{\mathrm{HL}}$$

where *E*_QM1+ptch23_^HL^ is the QM energy of system 1 truncated by HL atoms and embedded in the set of point charges modeling of systems 2 and 3 (but excluding the self-energy of the point charges). $E_{\mathrm{MM}1,q_{1}=0}^{\mathrm{HL}}$ is the MM energy of system 1, still truncated by HL atoms, but without any electrostatic interactions. Finally, $E_{\mathrm{MM}123,q_{1}=0}^{\mathrm{CL}}$ is the classical energy of all atoms in the model with CL atoms and with the charges of the QM region set to zero (to avoid double-counting of the electrostatic interactions). Thus, ComQum employs a subtractive scheme with electrostatic embedding and van der Waals link-atom corrections.^143^ No cutoff is used for any of the interactions in the three energy terms in eq 1. The geometry optimizations were continued until the energy change between two iterations was less than 2.6 J/mol (10^–6^ a.u.) and the maximum norm of the Cartesian gradients was below 10^–3^ a.u.

### 2.3 Spin States of the Metal Centers

Six of the studied systems feature a Zn­(II) ion at the metal center. Owing to its *d* ^10^ electronic configuration, Zn­(II) has a closed-shell singlet ground state with no unpaired electrons. In contrast, the metal centers in Mb-CmpII and P450-CmpII are Fe­(IV) ions, which may exist in either a quintet (*S* = 2; four unpaired electrons) or triplet (*S* = 1; two unpaired electrons) spin state, depending on the ligand environment and electronic structure. Based on previous spectroscopic and computational studies, which demonstrated that the most stable spin state for Fe­(IV) complexes in heme proteins is generally the triplet state,^103,144^ we employed the triplet spin configuration in all calculations for these systems. However, we also performed calculations for the quintet state (Table S2), confirming that the triplet state is indeed lower in energy by 36–177 kJ/mol across all our systems. For H-NOX, the heme Fe­(III) ion was modeled in the high-spin sextet (*S* = 5/2) state. The ⟨*S* ^2^⟩ values for these calculations (shown in Table S3) are close to the expected values, 2.02–2.12 and 8.75–8.79 (2 and 8.75 expected).

In the TLF variants, the active site contains two high-spin Fe­(III) ions (*d* ^5^ configuration) that are antiferromagnetically coupled. Under idealized octahedral symmetry, this results in a total spin state of *S* = 0. To describe this antiferromagnetic coupling within DFT, we employed the broken-symmetry (BS) formalism.^145,146^ Specifically, a single BS state was constructed, with one Fe­(III) ion bearing a net α-spin and the other a net β-spin. These BS states were generated either by the fragment-based approach proposed by Szilagyi and Winslow^147^ or by initially optimizing the high-spin configuration (all unpaired electrons aligned) and subsequently inverting the spin on one Fe center to obtain the desired BS state.^148^

### 2.4 QM Systems

Three different sizes of the QM region were employed in this study, referred to as the minimal (Min), intermediate (Int), and large (Big) QM systems. The minimal QM system comprised only the metal ion and its directly coordinated ligands. The intermediate QM system extended the Min region by including all groups forming hydrogen bonds with the metal ligands. In this case, backbone amide groups were modeled as CH_3_CONHCH_3_ fragments, and protein side chains were represented by their functional groups truncated at the CA–CB bond. The largest QM system included all functional groups located within 3.5 Å of any atom in the Min system. These Big QM regions were generated using our in-house script *changepdb* (version 2408), which automates the construction of QM regions.^149^

For HNOX-I5L, the Min and Int systems were constructed following this standard protocol. However, the standard Big QM region for this enzyme proved too large to achieve reliable convergence. Therefore, a reduced Big QM region was defined, which included all functional groups within 3.5 Å of any atom in a reduced Min region in which the porphyrin side chains were excluded (but the full heme cofactor was included in the Big QM system).

Similarly, a modified protocol was adopted for ADH, because we estimated the effect of the coenzyme on the p*K*_a_ of the metal-bound solvent molecule. In the Min system, the metal ion and its coordinating ligands were included along with the nicotinamide ring of the coenzyme (with the connecting carbon of the first ribose replaced by a hydrogen atom; not for ADH-apo). The Int system also incorporated the adjacent ribose ring, in which the CH_2_ group linked to the phosphate was replaced by a hydrogen atom. The Big QM system encompassed the complete coenzyme, all residues forming hydrogen bonds to it, and all functional groups within 3.5 Å of the Min region. These QM systems for ADH are illustrated in Figure S7 of the Supporting Information.

The sizes of the Min, Int, and Big systems for each protein are summarized in Table S1. For example, the Min, Int, and Big QM regions consisted of 40, 103, and 357 atoms for CA-WT, respectively (including hydrogen link atoms). Representative structures are shown in Figure S8 of the Supporting Information.

### 2.5 QM-Cluster Calculations

In some calculations, the QM system was immersed in a continuum solvent, employing the conductor-like screening model (COSMO),^150,151^ implemented in Turbomole. The default optimized COSMO atomic radii and a water solvent radius of 1.30 Å were employed to construct the solvent-accessible surface cavity,^152^ whereas radii of 2.11 and 1.72 Å were used for P and F, respectively,^153^ and 2.0 Å for Zn and Fe. Structures for the QM-cluster calculations were taken directly from the QM/MM calculations without further optimization. The dielectric constant of proteins has been much discussed, but typically, values of 4–20 have been used.^154,155^ We tested three values: 4, 20, and 80.

### 2.6 p*K*_a_ Calculations

To calculate the p*K*_a_ values from our theoretical results, we used eq 2.^156^

2 $$pK_{a}=\frac{\Delta G\left(A^{-}\right)-\Delta G\left(\mathrm{AH}\right)+\Delta G\left(H^{+}\right)}{RT\ln10}$$

where Δ*G*(H^+^) = −1131.0 kJ/mol, which includes the hydration free energy of a proton, the translational Gibbs free energy of a proton, and the change in reference state from 1 atm to 1 M, all calculated at 300 K and 1 atm pressure.^157^ Several values of this constant have been suggested and used,^20,157,158^ but it has little effect on our results, because we are mainly interested in relative p*K*_a_ values. All calculations were based on QM/MM-optimized structures. Δ*G*(A^–^) and Δ*G*(AH) were obtained from either the QM/MM or QM-cluster calculations, using either optimized structures or single-point energy calculations.

### 2.7 Quality Measures

To evaluate the performance of each computational method, we employed 12 quality metrics. These include the mean signed error (MSE), which quantifies systematic bias; the mean absolute deviation (MAD), which measures the average unsigned error; and the maximum absolute error (Max), which reflects the worst-case deviation. We also calculated the MAD and Max values after removing the systematic error (i.e., after subtracting the MSE), denoted as trMAD and trMax, respectively. This can be seen as a correction to Δ*G*(H^+^) in eq 2, or as a correction for all systematic errors in the approach used (similar to the widely used approach of employing a reference protonation reaction with a known p*K*_a_ value).^1–3^ Additionally, we evaluated the range of the calculated p*K*_a_ values (defined as the difference between the largest and smallest values) and the slope of the best regression line between the calculated and experimental p*K*_a_ values. The coefficient of determination (*R* ^2^) was computed to assess the quality of the linear fit, and two rank-based correlation coefficients—Spearman’s ρ and Kendall’s τ—were used to evaluate the consistency of predicted and experimental p*K*_a_ rankings. The latter considered all 66 possible pairs of p*K*_a_ values.

To allow a fair comparison of the computed ranges, we introduced a “relative range” (RelRange) metric, defined as the absolute deviation of the calculated range from the experimental range of 9.3 pu. Similarly, to penalize deviations from the ideal slope of 1 in a symmetric manner, we defined a “relative slope” (RelSlope): For slopes (*k*) greater than or equal to 1, the penalty was computed as 1 – (1/*k*), and for slopes less than 1, it was calculated as 1 – *k*. This formulation ensures similar penalization for under- and overestimation of the ideal slope.

Following our previous studies on the redox potentials of iron–sulfur clusters and blue copper proteins,^159,160^ we focused on quality metrics that reflect relative accuracy—i.e., those that are independent of systematic error (and therefore also on Δ*G*(H^+^) in eq 2). In particular, we emphasized trMAD, trMax, RelRange, RelSlope, *R* ^2^, ρ, and τ. Notably, correlation-based metrics such as *R* ^2^, ρ, and τ tend to favor methods that produce broad ranges of predicted values, while trMAD and trMax favor methods that yield uniformly small deviations across all systems.

To derive a balanced and comprehensive performance measure, we defined a quality score as the average rank of six key metrics: trMAD, trMax, RelRange, RelSlope, *R* ^2^, and τ among all tested methods (Av6). We excluded Spearman’s ρ from the final average because it strongly correlated with Kendall’s τ, thereby reducing redundancy. This combined ranking approach ensures that both accuracy in magnitude and consistency in trend across different systems are appropriately weighted in evaluating the predictive power of each computational method.

## 3 Results and Discussion

In this study, we have computed p*K*_a_ values for 12 acid–base groups located near metal centers in proteins (see Table 1), using 64 different computational protocols. In total, 768 p*K*_a_ values were obtained from 1536 energy calculations, considering both the protonated (AH) and deprotonated (A^–^) states of each group across all methods. The 64 protocols involved a systematic variation of five or six parameters: the type of the calculation (QM/MM or QM-cluster), the size of the QM region (Min, Int, and Big, as defined in Section 2.4), the density functional (TPSS or B3LYP), the basis set (def2-SV­(P) or def2-TZVPD, abbreviated as SV and TZ, respectively), and whether the MM environment (system 2) was allowed to relax (Rel) or not (Fix). For the QM-cluster (QM+COSMO) calculations, single-point energies were computed using structures optimized via QM/MM, with solvation modeled by COSMO using dielectric constants of 4, 20, or 80.

The full set of calculated p*K*_a_ values is presented in Tables S4–S7 in the Supporting Information. To assess the accuracy and consistency of each computational protocol, we employed a range of statistical quality measures, as described in Section 2.7.

### 3.1 Performance of QM/MM Methods

Table 2 summarizes the quality metrics obtained for the 16 QM/MM protocols tested in this study. These methods differ in terms of DFT functional (TPSS or B3LYP), basis set (SV or TZ), QM region size (Min, Int, or Big), treatment of the MM surroundings (fixed or relaxed), and whether the QM/MM geometry was used directly (Opt) or only for single-point energy calculations based on structures obtained at the TPSS/SV level. Overall, the QM/MM methods performed poorly compared to the QM-cluster approaches (discussed in the next section), with overall quality ranks ranging from 44 to 64 out of the 64 protocols assessed (i.e., only four QM-cluster methods have overall ranks worse than the best QM/MM methods).

**Table 2:** Tweleve Quality Metrics for Each QM/MM Protocol, Categorized by QM Region Size (Min, Int, Big) and Calculation Type (Geometry Optimization, Opt, or Single-Point Energy) Using Either the TPSS (TP) or B3LYP (B3) Functionals with the def2-SV­(P) (SV) or def2-TZVPD (TZ) Basis Sets^a^

QM system		Minimal QM system								Intermediate QM system				Big QM system			
Calculation type		Opt		Single Point						Opt	Single Point			Opt	Single Point		
Functional/basis set		TP/SV		TP/TZ		B3/SV		B3/TZ		TP/SV	TP/TZ	B3/SV	B3/TZ	TP/SV	TP/TZ	B3/SV	B3/TZ
Relaxed surroundings		No	Yes	No	Yes	No	Yes	No	Yes	No	No	No	No	No	No	No	No
Measure	MSE	33.4	17.6	32.0	17.0	33.3	17.2	31.6	16.4	26.0	24.7	25.7	24.6	27.7	28.3	27.5	27.9
	MAD	37.6	29.8	36.2	29.2	36.8	28.9	35.4	28.4	33.1	33.0	33.4	33.1	28.3	29.6	28.0	29.3
	Max	126.7	97.2	125.3	93.9	125.4	96.0	123.8	92.7	107.3	105.8	106.7	108.0	83.5	84.0	84.3	85.1
	trMAD	39.5	35.5	38.1	34.5	38.9	34.7	37.2	33.6	32.4	30.9	32.9	31.4	24.0	25.2	22.9	24.2
	trMax	93.2	79.7	93.2	76.9	92.1	78.8	92.1	76.3	81.3	81.1	81.0	83.4	55.8	55.7	56.8	57.2
	*R* ^2^	0.29	0.21	0.27	0.19	0.31	0.22	0.28	0.20	0.38	0.35	0.40	0.36	0.12	0.12	0.12	0.13
	Range	141.0	119.8	138.4	117.7	137.8	118.8	135.2	117.0	139.3	137.2	138.7	139.2	89.5	93.1	90.1	94.8
	RelRange	131.7	110.5	129.1	108.4	128.5	109.5	125.9	107.7	130.0	127.9	129.4	129.9	80.2	83.8	80.8	85.5
	Slope	10.7	8.0	10.0	7.4	10.7	8.0	9.9	7.3	10.3	9.7	10.6	9.9	4.0	4.2	4.0	4.5
	RelSlope	0.9	0.9	0.9	0.9	0.9	0.9	0.9	0.9	0.9	0.9	0.9	0.9	0.7	0.8	0.7	0.8
	ρ	0.75	0.55	0.71	0.49	0.75	0.61	0.75	0.48	0.69	0.71	0.71	0.71	0.00	0.04	0.00	0.02
	τ	0.52	0.33	0.48	0.30	0.52	0.36	0.52	0.27	0.48	0.52	0.52	0.52	–0.03	0.03	–0.03	0.03
Rank	MSE	64	40	62	38	63	39	61	37	56	54	55	53	58	60	57	59
	MAD	64	56	62	53	63	52	61	49	59	57	60	58	48	55	47	54
	Max	64	56	62	54	63	55	61	53	59	57	58	60	49	50	51	52
	trMAD	64	60	62	58	63	59	61	57	55	53	56	54	50	52	49	51
	trMax	64	56	63	54	61	55	62	53	59	58	57	60	50	49	51	52
	relRange	64	56	60	54	59	55	57	53	63	58	61	62	49	51	50	52
	relSlope	54	46	51	44	55	45	49	43	52	48	53	50	28	33	29	37
	*R* ^2^	34	43	37	47	33	40	35	46	30	32	29	31	51	52	50	49
	ρ	15	45	28	47	15	40	15	48	33	26	28	26	53	50	53	51
	τ	22	46	32	47	22	44	22	48	32	22	22	22	53	49	53	49
	Av6	50.3	51.2	50.8	50.7	48.8	49.7	47.7	50.0	48.5	45.2	46.3	46.5	46.8	47.7	47.0	48.3
	Sd6	18.1	7.0	13.4	5.4	17.1	7.6	16.1	5.2	14.1	14.9	16.5	16.3	9.3	7.3	8.9	5.7
	Total	61	64	63	62	58	59	53	60	57	44	47	48	49	53	50	55

a Calculations were performed with either a fixed or relaxed MM environment. The metrics include the mean signed error (MSE), mean absolute deviation (MAD), maximum absolute error (Max), and the corresponding measures after removal of the systematic error (trMAD, trMax), along with *R* ^2^, range, RelRange, slope, RelSlope, Spearman’s ρ, and Kendall’s τ. The lower section of the table reports the rank of each method, along with the average and standard deviation of the ranks of six selected metrics (trMAD, trMax, RelRange, RelSlope, *R* ^2^, τ; Av6 and Sd6), and the final rank, based on Av6. Lower ranks indicate better performance. The ranking also involves the QM-cluster methods in Tables 3 and 4.

Among the QM/MM variants, the best performance was obtained with the TPSS functional and fixed surroundings, with the intermediate QM region and the large basis set. However, all QM/MM methods yielded gravely overestimated ranges of calculated p*K*_a_ values (89–141 pu), exceeding the experimental range of 9.3 pu by far. This led to exaggerated slopes (4–11) and large absolute deviations from experimental data (trMAD values of 23–39 pu and trMax values of 56–93 pu).

Interestingly, the wide range of computed p*K*_a_ values yielded high correlation-based measures, such as Spearman’s ρ (up to 0.75) and Kendall’s τ (up to 0.52), particularly with the Min region. As previously discussed, these metrics can misleadingly favor methods that overestimate the variability of computed values. In contrast, the other quality measures (e.g., trMAD, trMax, and RelRange) indicate that these methods do not accurately reproduce the experimental trends.

As shown in eq 1, the QM/MM energy consists of a QM (*E*_QM1+ptch23_^HL^) and an MM component ( $E_{\mathrm{MM}123,q_{1}=0}^{\mathrm{CL}}-E_{\mathrm{MM}1,q_{1}=0}^{\mathrm{HL}}$ ). When the MM surrounding is not allowed to relax, the MM component is rather small (e.g., 0.3–12 pu in absolute terms for Min/TPSS/SV) compared to the QM component (3–147 pu; cf. Table S8). In this case, the MM term includes van der Waals and bonded interactions between the QM and MM regions. However, if the MM region is allowed to relax, the MM component increases to 3–31 pu, whereas the QM component decreases to 3–79 pu. This shift indicates that the MM surroundings relax in response to changes in the net charges of the AH and A^–^ states, leading to significant differences in the electrostatic interactions within the MM region itself (and not only between the MM and QM regions, as is the case when the MM region is fixed).

The poor results of the QM/MM methods arise primarily because the net charge of the QM region changes between the protonated and deprotonated forms, and the MM environment in QM/MM methods cannot adequately relax in response to these changes, especially when the MM region is fixed. This issue has also been observed in previous studies on redox potentials in metalloproteins.^159,160^ Consequently, due to their overall poor performance and lack of balance between accuracy and correlation, we excluded the 16 QM/MM methods from the final evaluation and ranking of the 48 QM-cluster approaches.

### 3.2 Results from QM-Cluster Calculations

The calculated p*K*_a_ values for the 48 studied QM-cluster methods are presented in Tables S5–S7, and the quality measures are listed in Tables 3 and 4. It can be seen that a dielectric constant of 4 gives quite poor results, with rankings of 25–48. It performs worse than ε = 20 and 80 with otherwise the same parameters, with only a single exception in each case. Moreover, ε = 80 gives better results than ε = 20 for 75% of the calculations; always with the Min QM region, but only for one calculation with the Big region (25%).

**Table 3:** Quality Metrics for Each QM-Cluster Protocol Using the Min QM System^a^

QM system		Min																							
Functional/basis set		TP/SV						TP/TZ						B3/SV						B3/TZ					
Relaxed surroundings		No			Yes			No			Yes			No			Yes			No			Yes		
Dielectric constant		4	20	80	4	20	80	4	20	80	4	20	80	4	20	80	4	20	80	4	20	80	4	20	80
Measure	MSE	24.5	15.3	13.4	23.7	14.2	12.6	23.4	15.2	13.5	22.0	13.4	11.6	23.0	15.0	13.3	22.3	13.9	12.1	22.3	14.6	13.0	21.1	13.0	11.2
	MAD	28.9	15.3	13.4	28.8	14.2	12.6	27.9	15.2	13.5	27.1	13.4	11.6	27.1	15.0	13.3	27.1	13.9	12.1	26.7	14.6	13.0	26.2	13.0	11.2
	Max	58.0	24.7	19.6	55.8	22.8	22.4	60.7	27.9	20.9	52.4	20.2	14.7	56.4	23.5	20.5	54.4	22.2	19.8	58.3	25.6	18.9	50.5	19.9	14.7
	trMAD	20.0	5.4	2.8	20.4	5.7	3.2	18.3	4.5	2.7	18.7	4.8	2.0	18.2	5.1	3.3	18.8	5.3	3.0	17.0	4.1	3.1	17.7	4.1	2.3
	trMax	37.7	9.4	6.3	38.1	10.1	9.8	37.3	12.7	7.4	37.5	10.6	5.0	35.5	8.6	7.2	36.5	9.7	7.7	36.0	11.0	5.9	36.8	10.6	5.0
	*R* ^2^	0.22	0.56	0.75	0.20	0.48	0.67	0.20	0.46	0.61	0.19	0.41	0.64	0.28	0.70	0.79	0.26	0.61	0.76	0.24	0.50	0.54	0.22	0.44	0.56
	Range	72.6	22.6	17.8	71.6	21.4	19.8	75.7	24.0	15.9	69.3	22.2	13.2	70.3	21.0	19.9	70.0	22.3	19.6	72.9	21.3	12.7	67.6	22.6	13.6
	RelRange	63.3	13.3	8.5	62.3	12.1	10.5	66.4	14.7	6.6	60.0	12.9	3.9	61.0	11.7	10.6	60.7	13.0	10.3	63.6	12.0	3.4	58.3	13.3	4.3
	Slope	4.6	2.4	1.9	4.5	2.3	1.9	4.3	2.0	1.5	4.1	1.8	1.3	4.9	2.6	2.1	4.9	2.5	1.9	4.4	2.0	1.5	4.2	1.8	1.2
	RelSlope	0.8	0.6	0.5	0.8	0.6	0.5	0.8	0.5	0.4	0.8	0.4	0.2	0.8	0.6	0.5	0.8	0.6	0.5	0.8	0.5	0.3	0.8	0.4	0.2
	ρ	0.72	0.83	0.94	0.53	0.77	0.85	0.71	0.83	0.87	0.56	0.74	0.78	0.72	0.87	0.91	0.66	0.79	0.84	0.74	0.78	0.69	0.64	0.76	0.67
	τ	0.52	0.67	0.53	0.36	0.58	0.70	0.48	0.64	0.73	0.39	0.55	0.64	0.52	0.73	0.76	0.45	0.64	0.73	0.55	0.58	0.55	0.45	0.61	0.55
Rank	MSE	48	36	28	47	32	17	46	35	30	42	29	12	45	34	26	43	31	15	44	33	23	41	24	6
	MAD	48	35	22	47	27	10	46	34	24	44	23	5	45	33	21	43	26	8	42	31	16	41	17	1
	Max	46	17	4	44	14	13	48	30	9	42	7	1	45	15	8	43	12	5	47	22	3	39	6	2
	trMAD	47	23	4	48	28	7	44	12	3	45	15	1	43	19	8	46	22	5	41	10	6	42	9	2
	trMax	47	9	4	48	14	12	45	19	6	46	16	2	41	8	5	43	10	7	42	17	3	44	15	1
	RelRange	46	14	5	45	11	7	48	19	4	42	12	2	44	9	8	43	13	6	47	10	1	41	15	3
	RelSlope	35	18	7	34	15	9	32	12	4	28	6	2	38	22	14	36	19	8	33	10	3	30	5	1
	*R* ^2^	33	16	3	34	23	5	35	24	7	36	28	6	29	4	1	30	8	2	31	21	19	32	26	15
	ρ	21	7	1	36	13	5	23	7	3	34	15	12	21	3	2	28	10	6	15	11	24	30	14	27
	τ	22	6	21	36	12	5	26	7	2	35	17	7	22	2	1	29	7	2	17	12	17	29	11	17
	Av6	38.3	14.3	7.3	40.8	17.2	7.5	38.3	15.5	4.3	38.7	15.7	3.3	36.2	10.7	6.2	37.8	13.2	5.0	35.2	13.3	8.2	36.3	13.5	6.5
	Sd6	10.2	6.2	6.8	6.9	6.8	2.7	8.6	6.2	1.9	6.9	7.2	2.5	8.8	8.1	5.0	7.3	6.1	2.5	10.7	4.6	7.8	6.7	7.2	7.4
	Total	42	13	6	48	19	7	42	14	2	44	15	1	36	9	4	40	10	3	35	11	8	37	12	5

a The entries are the same as in Table 2.

**Table 4:** Quality Metrics for Each QM-Cluster Protocol Using the Int and Big QM Systems^a^

QM system		Int												Big											
Functional/basis set		TP/SV			TP/TZ			B3/SV			B3/TZ			TP/SV			TP/TZ			B3/SV			B3/TZ		
Relaxed surroundings		No			No			No			No			No			No			No			No		
Dielectric constant		4	20	80	4	20	80	4	20	80	4	20	80	4	20	80	4	20	80	4	20	80	4	20	80
Measure	MSE	19.0	12.8	11.5	19.5	13.2	11.9	18.7	12.6	11.4	18.9	12.7	11.4	12.6	10.9	10.6	13.3	11.7	11.4	12.2	10.7	10.4	12.8	11.3	11.0
	MAD	19.0	12.8	11.5	19.5	13.2	11.9	18.7	12.6	11.4	18.9	12.7	11.4	15.0	13.3	12.9	13.7	12.2	11.9	16.0	14.5	14.2	14.4	13.0	12.7
	Max	50.7	31.2	27.1	45.2	25.9	21.7	51.1	31.5	27.4	44.8	25.4	21.3	30.0	26.0	25.1	28.9	25.0	24.1	31.1	27.1	26.2	29.7	25.7	24.8
	trMAD	10.1	5.2	4.8	10.0	5.0	4.2	9.9	5.6	5.2	9.8	5.5	4.7	9.3	5.7	6.3	8.9	4.8	5.0	10.2	6.6	7.0	9.6	5.6	5.8
	trMax	31.8	18.4	15.6	25.7	12.6	9.8	32.4	18.9	16.0	25.9	12.7	9.9	26.8	25.3	24.9	15.9	15.0	14.7	35.2	33.8	33.4	22.1	21.5	21.3
	*R* ^2^	0.49	0.59	0.58	0.44	0.58	0.58	0.52	0.60	0.58	0.45	0.56	0.55	0.00	0.00	0.00	0.00	0.00	0.01	0.00	0.00	0.01	0.01	0.00	0.00
	Range	52.1	35.4	32.1	46.5	27.0	23.2	53.2	37.3	33.9	47.1	26.4	23.0	40.2	36.5	35.9	27.6	24.3	23.5	50.0	46.2	45.3	34.9	31.9	31.2
	RelRange	42.8	26.1	22.8	37.2	17.7	13.9	43.9	28.0	24.6	37.8	17.1	13.7	30.9	27.2	26.6	18.3	15.0	14.2	40.7	36.9	36.0	25.6	22.6	21.9
	Slope	4.1	2.8	2.5	3.7	2.3	2.0	4.2	2.9	2.6	3.7	2.4	2.1	0.1	–0.2	–0.2	0.2	–0.1	–0.2	0.0	–0.3	–0.4	0.3	0.0	–0.1
	RelSlope	0.8	0.6	0.6	0.7	0.6	0.5	0.8	0.7	0.6	0.7	0.6	0.5	0.9	1.2	1.2	0.8	1.1	1.2	1.0	1.3	1.4	0.7	1.0	1.1
	ρ	0.73	0.68	0.56	0.73	0.74	0.61	0.80	0.65	0.60	0.69	0.73	0.64	–0.07	–0.16	–0.25	–0.01	–0.08	–0.21	0.01	–0.14	–0.24	0.07	–0.02	–0.10
	τ	0.58	0.52	0.42	0.52	0.58	0.42	0.64	0.48	0.42	0.45	0.58	0.48	–0.09	–0.15	–0.18	–0.03	–0.09	–0.15	0.00	–0.12	–0.15	0.00	–0.06	–0.09
Rank	MSE	39	21	11	40	25	14	37	19	8	38	20	9	18	4	2	27	13	10	16	3	1	22	7	5
	MAD	39	14	4	40	19	6	37	11	2	38	12	3	32	20	15	25	9	7	36	30	28	29	18	13
	Max	40	35	27	38	24	11	41	36	29	37	21	10	33	25	20	31	19	16	34	28	26	32	23	18
	trMAD	39	20	16	38	17	11	37	25	21	36	24	13	34	27	30	33	14	18	40	31	32	35	26	29
	trMax	36	26	23	33	18	11	37	27	25	34	20	13	35	32	31	24	22	21	40	39	38	30	29	28
	RelRange	39	29	26	36	22	17	40	32	27	37	21	16	33	31	30	23	20	18	38	35	34	28	25	24
	RelSlope	29	23	20	26	16	11	31	25	21	27	17	13	39	44	46	37	43	45	41	47	48	24	40	42
	*R* ^2^	22	10	13	27	14	12	20	9	11	25	17	18	45	44	41	42	43	38	48	40	37	39	47	46
	ρ	18	26	34	20	15	32	9	29	33	24	18	31	41	45	48	39	42	46	38	44	47	37	40	43
	τ	12	22	32	22	12	32	7	26	32	29	12	26	41	45	48	39	41	45	37	44	45	37	40	41
	Av6	29.5	21.7	21.7	30.3	16.5	15.7	28.7	24.0	22.8	31.3	18.5	16.5	37.8	37.2	37.7	33.0	30.5	30.8	40.7	39.3	39.0	32.2	34.5	35.0
	Sd6	10.8	6.5	6.9	6.3	3.4	8.3	12.8	7.8	7.1	5.0	4.1	5.1	4.7	8.0	8.4	7.9	13.2	13.3	3.9	5.8	6.3	5.8	9.0	9.1
	Total	26	21	21	27	17	15	25	24	23	30	20	17	40	38	39	32	28	29	47	46	45	31	33	34

a The entries are the same as in Table 2.

Somewhat surprisingly, the Min QM region gives the best results overall, except with ε = 4, with ranking ranging from 1 to 19. Likewise, the Big QM region always gives worse results than the corresponding calculations with the Int QM regions, with all other parameters the same. Moreover, it is generally unfavorable to relax the surroundings. It improves results for only 3 of 12 comparable methods (25%). Therefore, it was tested only with the Min QM region.

The two DFT methods give similar results. They are best for half of the 24 comparable methods each. However, TPSS is best for most of calculations with the Int and Big QM regions (83%), but the opposite is true for the Min QM region. In fact, the two DFT methods yield absolute p*K*_a_ values that agree within 1.4 pu on average.

On the other hand, the larger TZ basis set generally gives better results (67% of the comparable methods). This is especially clear for the Int and Big QM regions, for which TZ is better for all except for two calculations with ε = 4 (i.e., 83%). For the Min QM region, the SV basis set gives better results for the B3LYP calculations with ε = 20 and 80, whereas TZ is better for most (67%) of the TPSS calculations. The effect of the basis set on the p*K*_a_ values is somewhat larger than that of the DFT method, 2.7 pu on average, increasing with the size of the QM region (2.2, 2.6, and 3.8 pu for the Min, Int, and Big QM regions, respectively).

In total, the best-performing method is TPSS/TZ with the minimal QM region, relaxed surroundings, and a dielectric constant of 80 (abbreviated in the following as Min/TPSS/TZ/Rel/80). It gives the best trMAD (2.0 pu), the second-best trMax (5.0 pu), range (13.2 pu) and slope (1.3), the sixth-best *R* ^2^ (0.64), the seventh-best ρ (0.78), but only the twelfth-best τ (0.64). On the other hand, it is rather mediocre in absolute terms, with an MSE and MAD of 11.6 pu (i.e., the absolute p*K*_a_ values are always too positive), but the Max absolute error of 14.7 pu is the best among all tested methods.

The corresponding approach with fixed surroundings is the second-best method (Min/TPSS/TZ/Fix/80). It is slightly worse in most quality measures (e.g., trMAD = 2.7 and trMax = 7.4), but has the second-best τ (0.73) and also a better ρ (0.87). The third- and fourth-best approaches are the corresponding B3LYP calculations with the small basis set (Min/B3LYP/SV/Rel/80 and Min/B3LYP/SV/Fix/80). They give the best two results for *R* ^2^ (0.76–0.79) and τ (0.73–0.76), but worse trMAD (3.0–3.3 pu) and trMax (7–8 pu). The corresponding calculations with the large basis set rank five and eight, whereas the corresponding TPSS/SV calculations rank six and seven. The corresponding methods with ε = 20 rank 9–15 and 19 (Min/B3LYP/SV/Fix/20 is best). The best methods with the Int QM regions (ranking 15) use TPSS/TZ with fixed surroundings and ε = 80. Likewise, the best methods with the Big QM regions (ranking 28–29) use also TPSS/TZ with fixed surroundings and ε = 20 or 80.

In absolute terms, Big/B3LYP/SV/Fix/80 gives the lowest MSE (10 pu), whereas Min/B3LYP/TZ/Rel/80 gives the lowest MAD (11 pu) and Min/TPSS/TZ/Rel/80 gives the lowest Max (15 pu).

Employing instead a ranking based on each score divided by its range among all method changes the ranking only slightly (by four positions at most and by 1.5 positions on average). The best approach remains, and all the best eight methods employ the Min QM region and ε = 80 but with slight modifications in the order.

Figure 1 shows the results of the top four methods (after removing the MSE) compared with experimental data (the black line represents the ideal correlation). It can be seen that the four methods often agree in their predictions, e.g., they predict very similar p*K*_a_ values for P450, both CA systems, and TLF-C55S, with a spread of only ∼ 2 pu, whereas larger deviations are observed for ADH-NAD, with differences of up to 7.5 pu (approximately 4 pu on average). All methods give a too large range (13–20 pu) and a mediocre correlation (*R* ^2^ = 0.61–0.79). CA-E106Q, TLF-C59S, ADH-Apo, or ADH-NADH. ADH-TFE, TLX-C55S, and the two CA systems always give results below the ideal correlation line, whereas TLX-C59S, ADH-NADH, ADH-Apo, and P450 always give results above the ideal correlation line.

**Figure 1:**
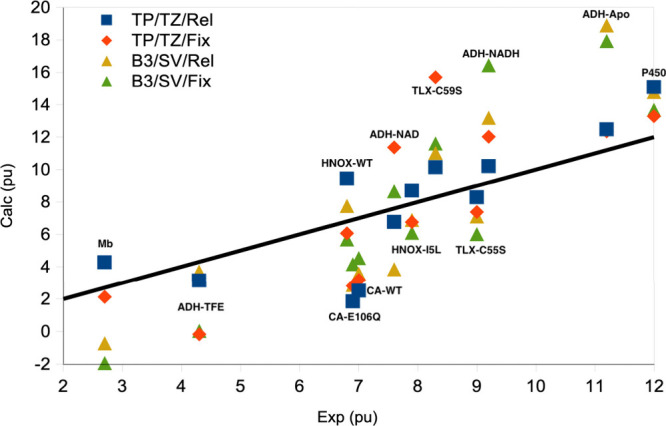
Experimental (Exp) and calculated (Calc) p*K*_a_ values (after removal of the MSE) for the four best methods (all employing the minimal QM region and ε = 80). The black line shows the ideal fit (*y* = *x*). Deviations from this line correspond to residual errors.

The performance of the individual systems varies and depends on the size of the QM model. For CA, the absolute errors are consistently small and similar across all three QM models, with MADs of 7–10 pu across all 48 QM-cluster methods. However, for TLF–C59S, performance is poor across most methods. It has always the largest error for the Min systems with small dielectric constants and typically the second largest error for the Int systems. For the Big QM system, it is the only system that gives too negative p*K*_a_ values (with Int, no system gives too negative p*K*_a_ values, and with Min only the two CA systems with ε = 4). ADH-Apo always gives the largest errors for the Int systems and it is always among the worst for the Min system with ε = 80, together with ADH-NAD, ADH-NADH, and P450. For the Big system, ADH-TFE always gives the largest error. After removal of the systematic error, ADH-Apo is still worst for the Int systems, whereas TLF-C59S is worst for the Big systems, and the two CA systems are worst for the Min models with ε = 4 and 20. With ε = 80, the errors are so small that many systems may give the worst results for the different variants, including all ADH, TLF, and CA systems.

The effect of the dielectric constant diminishes with the size of the QM region. For the Min systems, the mean absolute difference between the p*K*_a_ values estimated with ε = 20 and 4 is 17 pu, whereas for the Int and Big systems, the corresponding mean absolute differences are 7 and 5 pu. The mean absolute differences between ε = 80 and 20 are much smaller, 4, 2, and 1 pu for the Min, Int, and Big QM regions, respectively. However, there are large differences between the various systems, depending on the net charge of the models. Systems with net charges of −2/–3 (AH/A^–^; viz., the two TLF systems and P450 with the Min models, cf. Table S1) have the largest differences. Those with charges of 2/1 or −1/–2 have intermediate effects, and those with charges of 1/0 or 0/–1 have the smallest changes. For the Min and Int systems, there is a noticeable correlation between the net charge of the QM system and the magnitude of the deviations, with the largest errors observed for systems carrying the highest charges. In contrast, this correlation largely disappears for the Big QM regions.

In fact, the variation of the calculated p*K*_a_ values as a function of the net charge of the QM region seems to be the prime problem for the QM-cluster method, especially for the Min QM region and low values of ε. For example, the MSE is 47–53, 26–32, 25–31, 0–7, and −12 to −15 pu for the eight Min models with ε = 4 and QM-region charges of −2, −1, 0, 1, and 2 for the AH state, respectively. Admittedly, the statistics are poor (there are only two or three models of each charge), but the trend is quite clear. The trends remain also for the Int and Big QM models (32–34, 12–15, and 7–10 pu for Int QM models with net charges of −1, 0, and 1, and 22–23, 13–15, 6–9 and 1–2 pu for Int QM models with net charges of – 1, 0, 1, and 2, respectively. For ε = 20. The trend is blurred and for ε = 80, the ordering is essentially random (e.g., average MSEs of 14, 12, 17, 11, and 9 pu for the Min system with charges of – 2, – 1, 0, 1, and 2. This behavior explains why a large dielectric constant is required to obtain accurate results within the QM-cluster framework. At lower ε values, the strong charge dependence implies that separate calibration lines (i.e., MSE corrections) would be needed for each charge class, which would require a substantially larger data set. For the Big QM models, we could have aimed to obtain systems with the same net charge, but for the Min and Int systems, this is not possible because the charge depends on the net charge of the metal site and possible neutralizing groups in the second coordination sphere.

For new proteins, we recommend the use of the calibration line obtained in this study, i.e., to add the MSE for the method of choice to p*K*_a_ values calculated from eq 2. To estimate the stability of such a procedure, we calculated MSE leaving out one system and then predicted p*K*_a_ for that system using the new value of MSE. Repeating this procedure, leaving out all systems once, gave a range of leave-one-out MSE values of 0.7 pu for the best method, increasing trMAD and trMax by only 0.2 and 0.5 pu (to 2.2 and 5.5 pu). Similar results are obtained for all the best methods (e.g., increasing trMAD by 0.2–0.5 pu and trMax by 0.5–0.9 pu for the ten best methods), showing that our approach is quite stable.

### 3.3 Effect of Thermostatistical Corrections

Previous studies have indicated that thermostatistical corrections (zero-point energy and corrections to enthalpy and entropy from vibrational frequencies) have significant effects on calculated p*K*_a_ values.^20^ Therefore, we have also evaluated the effect of including thermostatistical corrections to the calculated p*K*_a_ values. The corrections were obtained from a frequency calculation of the Min model for each protein and protonation state, performed at the TPSS/def2-SV­(P) level for the isolated cluster model, after optimization with H-link atoms fixed in the QM/MM structures from the fixed surroundings. These corrections were added to the p*K*_a_ calculations of all 64 methods. The frequency calculations were performed with the ORCA program (Version 6.1.0).^161–163^ ORCA offers a more convenient platform for setting up and executing frequency calculations, particularly when enabling the BS approach in DFT calculations. These calculations also incorporated the D3BJ^137,138^ dispersion correction scheme and employed the auxiliary basis set def2/J.^136^ The corrections are shown in Table S9. They are always negative (decreasing the predicted p*K*_a_ values), ranging from −2.9 (ADH–TFE) to −8.1 pu (TLX-C55S). Since the calculated p*K*_a_ values without these corrections always were too positive (a positive MSE), this has an important effect on the absolute p*K*_a_ values, decreasing MSE for all methods by 5.8 pu and Max for most methods, typically by 6.7 pu, but with a variation from −8 to +4 pu (increasing Max only for the Big/TP/SV/Fix QM-cluster calculations with ε = 20 and 80).

However, for the p*K*_a_ values after removal of the MSE, the differences are more subtle. The QM/MM approaches are still worse than the QM-cluster approaches. The calculation with the Int QM region, TPSS, the large basis set, and fixed surroundings is best, ranking 43 among all methods, whereas the best QM/MM approach with the Big QM regions ranks 49, and the best with the Min QM region ranks 54. There are only five QM-cluster approaches that are worse than the best QM/MM approaches, all of which use the Big QM region and the SV basis set.

Concentrating on the QM-cluster calculations, the ranking remains similar, with a mean absolute change of only 1.5 positions. The Min/TP/TZ/Rel/80 approach still gives the best results. The (absolute) MSE = MAD = 5.8 pu and Max = 9.9 pu have strongly improved (by 5.8 and 4.8 pu), as with most of the other methods. trMax = 4.6 pu (5.0) and slope = 1.1 (1.3) have slightly improved (from 5.0 pu and 1.3), whereas trMAD = 2.0 pu is unchanged and the other measures have slightly deteriorated: range = 13.9 (before 13.2), *R* ^2^ = 0.54 (0.64), ρ = 0.73 (0.78), and τ_66_ = 0.61 (0.64). In fact, the corresponding approach without relaxation of the surroundings gives the same average rank (Av6) and therefore the same total rank. It gives better results for *R* ^2^, ρ, τ_66_ and the range (0.55, 0.83, 0.67, and 12.1 pu), but is worse for the other measures (including the MSE and Max). The corresponding methods with the small basis set rank 3 and 6 (with fixed and relaxed surroundings, respectively). The corresponding methods with B3LYP rank 4 and 5 (small basis set), 11 and 8 (large basis set). Thus, thermostatistical corrections reduce the systematic error (MSE), but they do not consistently improve the other quality measures. Therefore, it does not seem necessary for a general approach.

### 3.4 Some Method Variations

To assess the stability of the results, we explored several methodological variations. First, we performed a cross-check of the continuum-solvation treatment. For the Min/Fix/TPSS/SV protocol, QM-cluster calculations were repeated using two alternative continuum models: numerical solution of the Poisson–Boltzmann (PB) equation and the generalized Born (GB) approximation (OBC model II),^164^ as implemented in the Amber software.^119^ Electrostatic-potential charges derived from the QM calculations were employed, obtained using the Merz–Kollman scheme.^165^ All calculations were carried out with dielectric constants of ε = 4, 20, and 80, consistent with the COSMO setup. The results are collected in Table S10 in the Supporting Information. It can be seen that the calculated p*K*_a_ values differ somewhat from those obtained with the COSMO solvent, slightly more for GB (MADs of 8.1, 5.0, and 4.5 pu for ε = 4, 20, and 80, respectively) than for PB (7.0, 3.9, and 3.3 pu) and decreasing with ε. However, the trends are very similar, especially for PB (the difference between p*K*_a_ values calculated with ε = 20 and 4 differ by only 0.3 pu between PB and COSMO, and the corresponding difference between p*K*_a_ values calculated with ε = 80 and 20 is only 0.1 pu; for GB, the corresponding MADs are 3.5 and 1.1 pu). However, the quality measures are in general worse than for the COSMO results, except for the slope. This shows that COSMO results are more reliable and accurate than PB and GB (since they are obtained at the QM level without the need to represent the QM region with a charge model).

We then examined the effect of performing geometry optimization within the continuum solvent rather than via QM/MM. Specifically, we optimized the QM-cluster models for the Min/Fix/TP/SV/80 systems in the COSMO continuum solvent. Since the surrounding protein environment is missing, we kept the hydrogen link-atom positions fixed during optimization to avoid atoms moving in a manner not possible in the protein. The resulting p*K*_a_ values are shown in Table S11. It can be seen that they differ somewhat from those obtained with QM/MM optimized structures (the mean absolute difference is 3.3 pu). Compared to experimental p*K*_a_ values, results based on COSMO-optimized structures were worse for most quality measures (e.g., trMAD increased from 2.8 to 4.5 pu), but the slope and range improved slightly (to 15 pu and 1.5, respectively). Thus, we see no advantage in using COSMO-optimized structures.

We also examined the effect of extending relaxed MM region, increasing the cutoff of relaxed residues from 6 Å to 10 Å for the Min/Rel/TP/SV calculations. The results are shown in Table S12. The QM/MM results deteriorate, and the range of calculated p*K*_a_ values increases. However, for QM-cluster calculations based on these structures, the effect is different. For half of the systems, the change in the p*K*_a_ values is small, less than 1 pu. However, for CA, TLF-C59S, and the three ADH systems with water/OH^–^, the effect is larger, up to 6 pu. Interestingly, for ε = 20 and 80, all quality measures improve compared to the smaller relaxed system. Thus, this approach might be interesting to pursue in future studies.

Subsequently, we investigated the sensitivity of the calculated p*K*_a_ values to conformational sampling by performing a 100 ns MD simulation of CA-WT, employing a restrained bonded model for the metal site. Ten snapshots were used for subsequent p*K*_a_ calculations using two QM/MM protocols (with the SV and TZ basis sets) as well as QM-cluster calculations with dielectric constants of ε = 4, 20, and 80 (using the TZ basis set). The results, summarized in Table S13, show that the p*K*_a_ values calculated by QM/MM vary widely (a range of ∼30 pu, with a standard error of ∼3.5 pu), whereas the QM-Cluster calculations show a range of 3.9 pu and a standard error of 0.4 pu. The average p*K*_a_ values of the latter results differ from those of the single-conformation calculations by 0.4–1.3 pu. Consequently, ensemble averaging offers only limited gain while making the calculations more complicated (the metal site must be treated reasonably in MD simulations) and more costly (computational cost increases linearly with the number of snapshots).

In addition, we recalculated the p*K*_a_ values for the Min/Fix/TP/SV/80 series using four additional DFT functionals, r^2^SCAN-D4,^166–168^ M06-L,^169^ M06-D4,^167,168,170^ and ωB97M-V.^171^ The results are summarized in Table S14. All four functionals yield results that deviate somewhat more from TPSS than B3LYP does, with mean absolute differences of 2.2, 1.5, 2.1, and 3.9 pu, respectively (compared to 1.4 pu for B3LYP). Compared to the experimental data, none of the alternative functionals improves overall performance relative to TPSS. Only *R* ^2^ shows a slight improvement for the r^2^SCAN-D4 and M06-D4 calculations (0.78–0.79, compared to 0.74 for TPSS), similar to the trend observed for B3LYP. However, all the other quality measures are somewhat deteriorated with the new DFT methods. For example, the trMAD values increase to 3.1, 3.1, 3.6, and 3.5 pu for r^2^SCAN-D4, M06-L, M06-D4, and ωB97M-V, respectively, compared to 2.8 pu for TPSS. On the other hand, the absolute errors are reduced, e.g. giving MADs of 11.2, 11.9, 11.4, and 9.5 pu, compared to 13.4 pu for TPSS. Thus, with a proper choice of DFT method, it is possible to reduce the absolute errors in the calculated p*K*_a_ values, but for the relative values, the effect is limited. Similar trends have been observed in previous studies of redox potentials.^158,159^

Moreover, we attempted to perform microsolvation calculations for a subset of representative systems by adding 2–4 explicit water molecules at proper hydrogen-bonding positions in the Int QM region, followed by reoptimization in the COSMO continuum solvent. However, during the geometry optimizations, the added water molecules frequently reorganized and formed alternative interactions with nearby polar or charged groups, rather than remaining in their initially assigned positions. This behavior reflects the absence of the surrounding protein environment in the cluster model, which in the full system would impose steric and electrostatic constraints that stabilize specific water arrangements. Consequently, microsolvation effects cannot be reliably captured within a static QM-cluster framework without introducing additional constraints or sampling multiple configurations. This highlights the importance of explicit environment when second-sphere or solvent-mediated interactions are expected to play a critical role in determining p*K*_a_ values.

Finally, we considered the role of conformational flexibility in a specific residue. As discussed in the Supporting Information, the His-64 residue in CA, which participates in proton transfer to the active site, can adopt two conformations.^172^ Results presented so far involved the conformation in which the residue points inward into the active site. We also performed some calculations for the other conformation, in which His-64 points outward from the active site. The results in Table S15 show that a change in conformation consistently increases the p*K*_a_ of the Zn-bound water molecule by 5.0–5.1 pu in the QM/MM calculations, but decreases it by 0.3–0.4 pu in the QM-cluster calculations.

## 4 Conclusions

We have compared the accuracy of various QM/MM and QM-cluster calculations in estimating p*K*_a_ values of protonable groups coordinated to metal ions. We study 12 different test cases, originating from six different proteins (alcohol dehydrogenase, ferredoxin, carbonic anhydrase, myoglobin, cytochrome P450, and heme nitric oxide binding protein) and involving either Zn­(II), Fe­(III), or Fe­(IV). The reference p*K*_a_ values range from 2.7 to 12.0 (cf. Table 1). We have compared 64 different methods, 16 QM/MM methods, and 48 QM-cluster calculations in a COSMO continuum solvent (based on QM/MM structures). The methods differ in the size of the QM region (encompassing the metal and direct ligands, hydrogen-bonding groups, or a 3.5 Å surrounding), the DFT method (TPSS or B3LYP), the basis set (def2-SV­(P) or def2-TZVPD), whether the surroundings are relaxed, and the dielectric constant. The various methods are evaluated with a composite quality measure involving trMAD, trMax, the slope, the range, *R* ^2^, and τ.

The results show that ε = 4 gives poor results and ε = 80 is normally better than ε = 20. Interestingly, the Int QM region consistently outperforms the Big QM region, and the Min QM region typically gives the best results. Likewise, relaxed surroundings generally yield worse results than fixed ones, but this is not the case for the best-performing methods. In fact, the best eight approaches (with Min and ε = 80) give rather similar results. p*K*_a_ values predicted with the two DFT methods, with the two basis sets, and with fixed or relaxed surroundings agree within 1.4, 2.7, and 2.0 pu on average, respectively. The Min/TPSS/TZ/Rel/80 gives the best results, with trMAD = 2.0, trMax = 5.0, *R* ^2^ = 0.64, ρ = 0.78, and τ = 0.64. The systematic error (MSE) is quite large, 11.6 pu.

The results partly agree with our previous studies on the calculated redox potentials of iron–sulfur clusters and blue-copper proteins using similar methods,^159,160^ in particular that QM/MM calculations yield significantly worse results than QM-cluster calculations (owing to the insufficient relaxation of the surroundings to the change in the net charge of the metal site). On the other hand, the p*K*_a_ calculations appear to be much less sensitive to the surroundings, in that the Min QM region yields the best results, and relaxed surroundings often deteriorate them. This indicates that it is more important that the protonated and deprotonated structures have identical surroundings than that nearby groups are included in the QM calculations. On the other hand, this restricts the possibility of modeling second-sphere interactions. Moreover, the two DFT methods yield nearly identical results (an average difference of 1.4 pu for the calculated p*K*_a_ values), although TPSS is a meta-generalized gradient approximation functional, whereas B3LYP is a hybrid functional. This dependence is much smaller than for redox potentials, reflecting that p*K*_a_ values involve the same oxidation state of the metals, whereas redox potentials change with it.

Apparently, the best approach reflects more what is needed to obtain stable results with consistent trends, rather than physical realism. A dielectric constant of 80 is larger than what is normally used for proteins, but it is needed to dampen the dependence on the net charge of the QM region. Moreover, the advantage of the minimal QM region probably reflects that dynamic effects (many snapshots) are needed to properly model the effect of surroundings. However, it restricts the ability of the method to model mutations and second- or outer-sphere effects on the p*K*_a_ values (if such are of interest, the QM region necessarily needs to be enhanced, leading to a somewhat lower accuracy).

The accuracy of the p*K*_a_ calculations is slightly better than that of the redox potentials.^159,160^ The trMAD of the best method is 2.0 pu, which translates to 12 kJ/mol. The trMAD for the best method to calculate redox potentials of iron–sulfur clusters was 0.17 V, which translates to 16 kJ/mol. The trMax is 8.8 pu or 29 kJ/mol, whereas it was 0.44 V or 42 kJ/mol for the redox potentials. Still, the accuracy may seem rather discouraging, but we have demonstrated for the redox potentials that it is sufficient to determine the actual oxidation states of both Fe_4_S_4_ clusters and the FeMo and P clusters in nitrogenase.^159,160,173^ We expect that the accuracy of the p*K*_a_ values will be sufficient to determine the proper protonation states of metal clusters in proteins, such as nitrogenase, lytic polysaccharide monooxygenases, and multicopper oxidases, which will be our focus in future studies.

## Supplementary Material





## Data Availability

The data underlying this study are available in the published article and the .

## Notes

Coordinates of all studied systems are provided in a separate zip file archive (ZIP)
Further description of the studied systems; Example figures of the three system sizes for ADH-TFE and CA-WT; Details of the system setup; Results for the quintet state for Mb and P450; ⟨*S* ^2^⟩ values for the open-shell systems; Raw calculated p*K*_a_ values with all methods; QM and MM components of the calculated p*K*_a_ values; Thermostatistical corrections; Method variations (PDF)

## References

[1] Seybold P. G., Shields G. C. (2015). Computational Estimation of pKa Values. Wiley Interdiscip. Rev. Comput. Mol. Sci..

[2] Riccardi D., Schaefer P., Cui Q. (2005). pKa Calculations
in Solution and
Proteins with QM/MM Free Energy Perturbation Simulations: A Quantitative
Test of QM/MM Protocols. J. Phys. Chem. B.

[3] Sastre S., Casasnovas R., Muñoz F., Frau J. (2013). Isodesmic Reaction
for pKa Calculations of Common Organic Molecules. Theor. Chem. Acc..

[4] Li C., Jia Z., Chakravorty A., Pahari S., Peng Y., Basu S., Koirala M., Panday S. K., Petukh M., Li L., Alexov E. (2019). DelPhi Suite: New Developments and Review of Functionalities. J. Comput. Chem..

[5] Søndergaard C. R., Olsson M. H. M., Rostkowski M., Jensen J. H. (2011). Improved Treatment
of Ligands and Coupling Effects in Empirical Calculation and Rationalization
of pKa Values. J. Chem. Theory Comput..

[6] Olsson M. H.
M., Søndergaard C. R., Rostkowski M., Jensen J. H. (2011). PROPKA3: Consistent Treatment of
Internal and Surface
Residues in Empirical pK_a_ Predictions. J. Chem. Theory Comput..

[7] Zanetti-Polzi L., Daidone I., Amadei A. (2020). Fully Atomistic
Multiscale Approach
for pKa Prediction. J. Phys. Chem. B.

[8] Liptak M. D., Shields G. C. (2001). Accurate pKa Calculations for Carboxylic Acids Using
Complete Basis Set and Gaussian-n Models Combined with CPCM Continuum
Solvation Methods. J. Am. Chem. Soc..

[9] Gross K. C., Seybold P. G., Hadad C. M. (2002). Comparison
of Different Atomic Charge
Schemes for Predicting pKa Variations in Substituted Anilines and
Phenols. Int. J. Quantum Chem..

[10] Tao J., Perdew J. P., Staroverov V. N., Scuseria G. E. (2003). Climbing the Density
Functional Ladder: Nonempirical Meta-Generalized Gradient Approximation
Designed for Molecules and Solids. Phys. Rev.
Lett..

[11] Liptak M. D., Gross K. C., Seybold P. G., Feldgus S., Shields G. C. (2002). Absolute
pKa Determinations for Substituted Phenols. J. Am. Chem. Soc..

[12] Abul
Kashem Liton M., Idrish Ali M., Tanvir Hossain M. (2012). Accurate pKa
Calculations for Trimethylaminium Ion with a Variety of Basis Sets
and Methods Combined with CPCM Continuum Solvation Methods. Comput. Theor. Chem..

[13] Toth A. M., Liptak M. D., Phillips D. L., Shields G. C. (2001). Accurate
Relative
PK a Calculations for Carboxylic Acids Using Complete Basis Set and
Gaussian-n Models Combined with Continuum Solvation Methods. J. Chem. Phys..

[14] Satchell J. F., Smith B. J. (2002). Calculation of Aqueous
Dissociation Constants of 1,2,4-Triazole
and Tetrazole: A Comparison of Solvation Models. Phys. Chem. Chem. Phys..

[15] Gross K. C., Seybold P. G., Peralta-Inga Z., Murray J. S., Politzer P. (2001). Comparison
of Quantum Chemical Parameters and Hammett Constants in Correlating
pKa Values of Substituted Anilines. J. Org.
Chem..

[16] Charif I. E., Mekelleche S. M., Villemin D., Mora-Diez N. (2007). Correlation
of Aqueous pKa Values of Carbon Acids with Theoretical Descriptors:
A DFT Study. J. Mol. Struct. THEOCHEM.

[17] Namazian M., Zakery M., Noorbala M. R., Coote M. L. (2008). Accurate Calculation
of the pKa of Trifluoroacetic Acid Using High-Level Ab Initio Calculations. Chem. Phys. Lett..

[18] Casasnovas R., Ortega-Castro J., Frau J., Donoso J., Muñoz F. (2014). Theoretical
pKa Calculations with Continuum Model Solvents, Alternative Protocols
to Thermodynamic Cycles. Int. J. Quantum Chem..

[19] Li H., Robertson A. D., Jensen J. H. (2004). The Determinants of Carboxyl pKa
Values in Turkey Ovomucoid Third Domain. Proteins
Struct. Funct. Bioinforma..

[20] Li H., Hains A. W., Everts J. E., Robertson A. D., Jensen J. H. (2002). The Prediction of Protein pKa’s
Using QM/MM:
The pKa of Lysine 55 in Turkey Ovomucoid Third Domain. J. Phys. Chem. B.

[21] Jensen J. H., Li H., Robertson A. D., Molina P. A. (2005). Prediction and Rationalization of
Protein pKa Values Using QM and QM/MM Methods. J. Phys. Chem. A.

[22] Kamerlin S. C. L., Haranczyk M., Warshel A. (2009). Progress in Ab Initio QM/MM Free-Energy
Simulations of Electrostatic Energies in Proteins: Accelerated QM/MM
Studies of pKa, Redox Reactions and Solvation Free Energies. J. Phys. Chem. B.

[23] Yu H., Ratheal I. M., Artigas P., Roux B. (2011). Protonation of Key
Acidic Residues Is Critical for the K^+^-Selectivity of the
Na/K Pump. Nat. Struct. Mol. Biol..

[24] Sakipov S. N., Flores-Canales J. C., Kurnikova M. G. (2019). A Hierarchical Approach to Predict
Conformation-Dependent Histidine Protonation States in Stable and
Flexible Proteins. J. Phys. Chem. B.

[25] Goh G. B., Hulbert B. S., Zhou H., Brooks C. L. (2014). Constant pH Molecular
Dynamics of Proteins in Explicit Solvent with Proton Tautomerism. Proteins.

[26] Wallace J. A., Shen J. K. (2011). Continuous Constant
pH Molecular Dynamics in Explicit
Solvent with pH-Based Replica Exchange. J. Chem.
Theory Comput..

[27] Arthur E. J., Yesselman J. D., Brooks C. L. (2011). Predicting Extreme pKa Shifts in
Staphylococcal Nuclease Mutants with Constant pH Molecular Dynamics. Proteins Struct. Funct. Bioinforma..

[28] Lee M. S., Salsbury F. R., Brooks C. L. (2004). Constant-pH
Molecular Dynamics Using
Continuous Titration Coordinates. Proteins Struct.
Funct. Bioinforma..

[29] Meng Y., Roitberg A. E. (2010). Constant pH Replica Exchange Molecular Dynamics in
Biomolecules Using a Discrete Protonation Model. J. Chem. Theory Comput..

[30] Bürgi R., Kollman P. A., Van Gunsteren W. F. (2002). Simulating
Proteins at Constant pH:
An Approach Combining Molecular Dynamics and Monte Carlo Simulation. Proteins Struct. Funct. Bioinform..

[31] Swails J. M., Roitberg A. E. (2012). Enhancing Conformation
and Protonation State Sampling
of Hen Egg White Lysozyme Using pH Replica Exchange Molecular Dynamics. J. Chem. Theory Comput..

[32] Baptista A. M., Teixeira V. H., Soares C. M. (2002). Constant-pH
Molecular Dynamics Using
Stochastic Titration. J. Chem. Phys..

[33] Khandogin J., Brooks C. L. (2005). Constant
pH Molecular Dynamics with Proton Tautomerism. Biophys. J..

[34] Mongan J., Case D. A., McCammon J. A. (2004). Constant pH Molecular
Dynamics in
Generalized Born Implicit Solvent. J. Comput.
Chem..

[35] Khandogin J., Brooks C. L. (2006). Toward the Accurate First-Principles Prediction of
Ionization Equilibria in Proteins. Biochemistry.

[36] Lee J., Miller B. T., Damjanović A., Brooks B. R. (2014). Constant pH Molecular
Dynamics in Explicit Solvent with Enveloping Distribution Sampling
and Hamiltonian Exchange. J. Chem. Theory Comput..

[37] Meng Y., Sabri Dashti D., Roitberg A. E. (2011). Computing Alchemical Free Energy
Differences with Hamiltonian Replica Exchange Molecular Dynamics (H-REMD)
Simulations. J. Chem. Theory Comput..

[38] Williams S.
L., de Oliveira C. A. F., McCammon J. A. (2010). Coupling Constant pH Molecular Dynamics
with Accelerated Molecular Dynamics. J. Chem.
Theory Comput..

[39] Swails J. M., York D. M., Roitberg A. E. (2014). Constant pH Replica Exchange Molecular
Dynamics in Explicit Solvent Using Discrete Protonation States: Implementation,
Testing, and Validation. J. Chem. Theory Comput..

[40] Barroso
daSilva F. L., Dias L. G. (2017). Development of Constant-pH Simulation
Methods in Implicit Solvent and Applications in Biomolecular Systems. Biophys. Rev..

[41] Liu J., Swails J., Zhang J. Z. H., He X., Roitberg A. E. (2018). A Coupled
Ionization-Conformational Equilibrium Is Required to Understand the
Properties of Ionizable Residues in the Hydrophobic Interior of Staphylococcal
Nuclease. J. Am. Chem. Soc..

[42] Lu B., Cheng X., Huang J., McCammon J. A. (2009). An Adaptive Fast
Multipole Boundary Element Method for Poisson- Boltzmann Electrostatics. J. Chem. Theory Comput..

[43] Jo S., Vargyas M., Vasko-Szedlar J., Roux B., Im W. (2008). PBEQ-Solver
for Online Visualization of Electrostatic Potential of Biomolecules. Nucleic Acids Res..

[44] Holst M., Baker N., Wang F. (2000). Adaptive Multilevel
Finite Element
Solution of the Poisson-Boltzmann Equation I. Algorithms and Examples. J. Comput. Chem..

[45] Rocchia W., Alexov E., Honig B. (2001). Extending
the Applicability of the
Nonlinear Poisson-Boltzmann Equation: Multiple Dielectric Constants
and Multivalent Ions. J. Phys. Chem. B.

[46] Feig M., Brooks C. L. (2004). Recent Advances in the Development
and Application of Implicit Solvent Models in Biomolecule Simulations. Curr. Opin. Struct. Biol..

[47] Feig M., Onufriev A., Lee M. S., Im W., Case D. A., Brooks C. L. (2004). Performance Comparison
of Generalized
Born and Poisson Methods in the Calculation of Electrostatic Solvation
Energies for Protein Structures. J. Comput.
Chem..

[48] Teixeira V. H., Cunha C. A., Machuqueiro M., Oliveira A. S. F., Victor B. L., Soares C. M., Baptista A. M. (2005). On the Use of Different Dielectric
Constants for Computing Individual and Pairwise Terms in Poisson-Boltzmann
Studies of Protein Ionization Equilibrium. J.
Phys. Chem. B.

[49] Gilson M. K., Rashin A., Fine R., Honig B. (1985). On the Calculation
of Electrostatic Interactions in Proteins. J.
Mol. Biol..

[50] Baker N. A. (2005). Improving
Implicit Solvent Simulations: A Poisson-Centric View. Curr. Opin. Struct. Biol..

[51] Bashford D., Karplus M. (1990). pKa’s of Ionizable
Groups in Proteins: Atomic
Detail from a Continuum Electrostatic Model. Biochemistry.

[52] Dolinsky T. J., Nielsen J. E., McCammon J. A., Baker N. A. (2004). PDB2PQR: An Automated
Pipeline for the Setup of Poisson-Boltzmann Electrostatics Calculations. Nucleic Acids Res..

[53] Potter M. J., Gilson M. K., McCammon J. A. (1994). Small Molecule pKa Prediction with
Continuum Electrostatics Calculations. J. Am.
Chem. Soc..

[54] Havranek J. J., Harbury P. B. (1999). Tanford-Kirkwood Electrostatics for Protein Modeling. Proc. Natl. Acad. Sci. U. S. A..

[55] Reynolds J. A., Gilbert D. B., Tanford C. (1974). Empirical
Correlation between Hydrophobic
Free Energy and Aqueous Cavity Surface Area. Proc. Natl. Acad. Sci. U. S. A..

[56] Gilson M. K. (1993). Multiple-Site
Titration and Molecular Modeling: Two Rapid Methods for Computing
Energies and Forces for Ionizable Groups in Proteins. Proteins Struct. Funct. Bioinform..

[57] Lim C., Bashford D., Karplus M. (1991). Absolute pKa
Calculations with Continuum
Dielectric Methods. J. Phys. Chem..

[58] Antosiewicz J., McCammon J. A., Gilson M. K. (1996). The Determinants of pKa’s
in Proteins. Biochemistry.

[59] Antosiewicz J., McCammon J. A., Gilson M. K. (1994). Prediction
of pH-Dependent Properties
of Proteins. J. Mol. Biol..

[60] Zhu Z., Gunner M. R. (2005). Energetics of Quinone-Dependent
Electron and Proton
Transfers in Rhodobacter sphaeroides Photosynthetic Reaction Centers. Biochemistry.

[61] Georgescu R. E., Alexov E. G., Gunner M. R. (2002). Combining Conformational Flexibility
and Continuum Electrostatics for Calculating pKa’s in Proteins. Biophys. J..

[62] Rabenstein B., Ullmann G. M., Knapp E.-W. (1998). Energetics
of Electron-Transfer and
Protonation Reactions of the Quinones in the Photosynthetic Reaction
Center of Rhodopseudomonas viridis. Biochemistry.

[63] Spassov V.
Z., Luecke H., Gerwert K., Bashford D. (2001). pKa Calculations Suggest
Storage of an Excess Proton in a Hydrogen-Bonded Water Network in
Bacteriorhodopsin. J. Mol. Biol..

[64] Sandberg L., Edholm O. (1999). A Fast and Simple Method to Calculate Protonation States
in Proteins. Proteins Struct. Funct. Bioinform..

[65] Alexov E. G., Gunner M. R. (1999). Calculated Protein
and Proton Motions Coupled to Electron
Transfer: Electron Transfer from QA to QB in Bacterial Photosynthetic
Reaction Centers. Biochemistry.

[66] Song Y., Mao J., Gunner M. R. (2003). Calculation
of Proton Transfers in Bacteriorhodopsin
BR and M Intermediates. Biochemistry.

[67] Muegge I., Qi P. X., Wand A. J., Chu Z. T., Warshel A. (1997). The Reorganization
Energy of Cytochrome c Revisited. J. Phys. Chem.
B.

[68] Warwicker J., Watson H. C. (1982). Calculation of the Electric Potential in the Active
Site Cleft Due to α-Helix Dipoles. J.
Mol. Biol..

[69] Cole C., Warwicker J. (2002). Side-Chain Conformational Entropy
at Protein-Protein
Interfaces. Protein Sci..

[70] Koehl P., Delarue M. (1994). Application of a Self-Consistent
Mean Field Theory
to Predict Protein Side-Chains Conformation and Estimate Their Conformational
Entropy. J. Mol. Biol..

[71] You T. J., Bashford D. (1995). Conformation and Hydrogen
Ion Titration of Proteins:
A Continuum Electrostatic Model with Conformational Flexibility. Biophys. J..

[72] Warwicker J. (1986). Continuum
Dielectric Modelling of the Protein-Solvent System, and Calculation
of the Long-Range Electrostatic Field of the Enzyme Phosphoglycerate
Mutase. J. Theor. Biol..

[73] Kieseritzky G., Knapp E.-W. (2008). Optimizing pKa Computation
in Proteins with pH Adapted
Conformations. Proteins Struct. Funct. Bioinform..

[74] Beroza P., Case D. A. (1996). Including Side Chain
Flexibility in Continuum Electrostatic
Calculations of Protein Titration. J. Phys.
Chem..

[75] Barth P., Alber T., Harbury P. B. (2007). Accurate, Conformation-Dependent
Predictions of Solvent Effects on Protein Ionization Constants. Proc. Natl. Acad. Sci. U. S. A..

[76] Alexov E. G., Gunner M. R. (1997). Incorporating Protein
Conformational Flexibility into
the Calculation of pH-Dependent Protein Properties. Biophys. J..

[77] Song Y., Mao J., Gunner M. R. (2009). MCCE2:
Improving Protein pKa Calculations with Extensive
Side Chain Rotamer Sampling. J. Comput. Chem..

[78] Cvitkovic J. P., Pauplis C. D., Kaminski G. A. (2019). PKA17—A Coarse-Grain Grid-Based
Methodology and Web-Based Software for Predicting Protein pKa Shifts. J. Comput. Chem..

[79] Tan K. P., Nguyen T. B., Patel S., Varadarajan R., Madhusudhan M. S. (2013). Depth: A Web Server to Compute Depth,
Cavity Sizes,
Detect Potential Small-Molecule Ligand-Binding Cavities and Predict
the pKa of Ionizable Residues in Proteins. Nucleic
Acids Res..

[80] Milletti F., Storchi L., Cruciani G. (2009). Predicting Protein pKa by Environment
Similarity. Proteins Struct. Funct. Bioinforma..

[81] Bas D. C., Rogers D. M., Jensen J. H. (2008). Very Fast
Prediction and Rationalization
of pKa Values for Protein-Ligand Complexes. Proteins Struct. Funct. Bioinform..

[82] Sinha V., Laan J. J., Pidko E. A. (2021). Accurate
and Rapid Prediction of
pKa of Transition Metal Complexes: Semiempirical Quantum Chemistry
with a Data-Augmented Approach. Phys. Chem.
Chem. Phys..

[83] Pettersson G., Eklund H. (1987). Electrostatic Effects of Bound NADH and NAD^+^ on Ionizing Groups in Liver Alcohol Dehydrogenase. Eur. J. Biochem..

[84] Merz K. M. (1991). Determination
of pKa’s of Ionizable Groups in
Proteins: the pKa of Glu 7 and 35 in hen eggs white lysozyme and Glu
106 in human carbonic anhydrase. J. Am. Chem.
Soc..

[85] Riccardi D., Cui Q. (2007). pKa Analysis for the Zinc-Bound Water in Human Carbonic Anhydrase
II: Benchmark for “Multiscale” QM/MM Simulations and
Mechanistic Implications. J. Phys. Chem. A.

[86] Riccardi D., Yang S., Cui Q. (2010). Proton Transfer
Function of Carbonic
Anhydrase: Insights from QM/MM Simulations. Biochimica et Biophysica Acta - Proteins and Proteomics..

[87] Maupin C. M., McKenna R., Silverman D. N., Voth G. A. (2009). Elucidation of the
Proton Transport Mechanism in Human Carbonic Anhydrase II. J. Am. Chem. Soc..

[88] Duda D., Tu C., Qian M., Laipis P., Agbandje-McKenna M., Silverman D. N., McKenna R. (2001). Structural and Kinetic Analysis of
the Chemical Rescue of the Proton Transfer Function of Carbonic Anhydrase
II. Biochemistry.

[89] Jiao D., Rempe S. B. (2012). Combined Density
Functional Theory (DFT) and Continuum
Calculations of pKa in Carbonic Anhydrase. Biochemistry.

[90] Hu H., Yang W. (2008). Free Energies of Chemical
Reactions in Solution and in Enzymes with
Ab Initio Quantum Mechanics/Molecular Mechanics Methods. Annu. Rev. Phys. Chem..

[91] Warshel A., Levitt M. (1976). Theoretical Studies
of Enzymic Reactions: Dielectric,
Electrostatic and Steric Stabilization of the Carbonium Ion in the
Reaction of Lysozyme. J. Mol. Biol..

[92] Gao J., Truhlar D. G. (2002). Quantum
Mechanical Methods for Enzyme Kinetics. Annu.
Rev. Phys. Chem..

[93] Martí S., Roca M., Andrés J., Moliner V., Silla E., Tuñón I., Bertrán J. (2004). Theoretical Insights in Enzyme Catalysis. Chem. Soc. Rev..

[94] Van
Der Kamp M. W., Mulholland A. J. (2013). Combined Quantum Mechanics/Molecular
Mechanics (QM/MM) Methods in Computational Enzymology. Biochemistry.

[95] Xu D., Cui Q., Guo H. (2014). Quantum Mechanical/Molecular
Mechanical Studies of
Zinc Hydrolases. Int. Rev. Phys. Chem..

[96] Boulanger E., Harvey J. N. (2018). QM/MM Methods for Free Energies and Photochemistry. Curr. Opin. Struct. Biol..

[97] Senn H. M., Thiel W. (2009). QM/MM Methods for Biomolecular Systems. Angew.
Chemie - Int. Ed..

[98] Ryde U. (2016). QM/MM Calculations
on Proteins. Methods Enzymol..

[99] Siegbahn P. E. M., Himo F. (2011). The Quantum Chemical
Cluster Approach for Modeling
Enzyme Reactions. Wiley Interdiscip. Rev. Comput.
Mol. Sci..

[100] Colonna-Cesari F., Perahia D., Karplus M., Eklund H., Brädén C. I., Tapia O. (1986). Interdomain Motion
in Liver Alcohol Dehydrogenase. Structural and Energetic Analysis
of the Hinge Bending Mode. J. Biol. Chem..

[101] Yeh A. P., Ambroggio X. I., Andrade S. L. A., Einsle O., Chatelet C., Meyer J., Rees D. C. (2002). High Resolution
Crystal Structures of the Wild Type and Cys-55 → Ser and Cys-59
→ Ser Variants of the Thioredoxin-like [2Fe-2S] Ferredoxin
from Aquifex Aeolicus. J. Biol. Chem..

[102] Fisher S. Z., Maupin C. M., Budayova-Spano M., Govindasamy L., Tu C., Agbandje-McKenna M., Silverman D. N., Voth G. A., McKenna R. (2007). Atomic Crystal and
Molecular Dynamics Simulation Structures of Human Carbonic Anhydrase
II: Insights into the Proton Transfer Mechanism. Biochemistry.

[103] Hersleth H.-P., Uchida T., Røhr A. K., Teschner T., Schuonemann V., Kitagawa T., Trautwein A. X., Goorbitz C. H., Andersson K. K. (2007). Crystallographic
and Spectroscopic
Studies of Peroxide-Derived Myoglobin Compound II and Occurrence of
Protonated Fe^IV^-O. J. Biol. Chem..

[104] Zhao B., Guengerich F. P., Bellamine A., Lamb D. C., Izumikawa M., Lei L., Podust L. M., Sundaramoorthy M., Kalaitzis J. A., Reddy L. M., Kelly S. L., Moore B. S., Stec D., Voehler M., Falck J. R., Shimada T., Waterman M. R. (2005). Binding
of Two Flaviolin Substrate
Molecules, Oxidative Coupling, and Crystal Structure of Streptomyces
Coelicolor A3(2) Cytochrome P450 158A2. J. Biol.
Chem..

[105] Olea C., Kuriyan J., Marletta M. A. (2010). Modulating Heme
Redox Potential through Protein-Induced Porphyrin Distortion. J. Am. Chem. Soc..

[106] Pellicena P., Karow D. S., Boon E. M., Marletta M. A., Kuriyan J. (2004). Crystal Structure of an Oxygen-Binding
Heme Domain
Related to Soluble Guanylate Cyclases. Proc.
Natl. Acad. Sci. U. S. A..

[107] Bahnson B. J., Colby T. D., Chin J. K., Goldstein B. M., Klinman J. P. (1997). A Link between Protein Structure
and Enzyme Catalyzed
Hydrogen Tunneling. Proc. Natl. Acad. Sci. U.
S. A..

[108] Kvassman J., Pettersson G. (1980). Effect of pH on the Binding of Decanoate
and Trifluoroethanol to Liver Alcohol Dehydrogenase. Eur. J. Biochem..

[109] Kvassman J., Pettersson G. (1979). Effect of
pH on Coenzyme Binding
to Liver Alcohol Dehydrogenase. Eur. J. Biochem..

[110] DeTraglia M. C., Schmidt J., Dunn M. F., McFarland J. T. (1977). Liver Alcohol
Dehydrogenase Coenzyme Reaction Rates. J. Biol.
Chem..

[111] Shore J. D., Gutfreund H., Brooks R. L., Santiago D., Santiago P. (1974). Proton Equilibria and Kinetics in the Liver Alcohol
Dehydrogenase Reaction Mechanism. Biochemistry.

[112] Andersson P., Kvassman J., Lindström A., Oldén B., Pettersson G. (1981). Effect of NADH on the pKa of Zinc-Bound
Water in Liver Alcohol Dehydrogenase. Eur. J.
Biochem..

[113] Subramanian S., Duin E. C., Fawcett S. E. J., Armstrong F. A., Meyer J., Johnson M. K. (2015). Spectroscopic and
Redox Studies of
Valence-Delocalized [Fe_2_S_2_]^+^ Centers
in Thioredoxin-like Ferredoxins. J. Am. Chem.
Soc..

[114] Steiner H., Jonsson B.-H., Lindskog S. (1975). The Catalytic Mechanism
of Carbonic Anhydrase. Hydrogen-Isotope Effects on the Kinetic Parameters
of the Human C Isoenzyme. Eur. J. Biochem..

[115] Liang Z., Xue Y., Behravan G., Jonsson B. -H, Lindskog S. (1993). Importance of the Conserved
Active-site Residues Try7,
Glu106 and Thr199 for the Catalytic Function of Human Carbonic Anhydrase
II. Eur. J. Biochem..

[116] Yosca T. H., Behan R. K., Krest C. M., Onderko E. L., Langston M. C., Green M. T. (2014). Setting an Upper
Limit on the Myoglobin
Iron­(IV)­Hydroxide pKa: Insight into Axial Ligand Tuning in Heme Protein
Catalysis. J. Am. Chem. Soc..

[117] Yosca T. H., Rittle J., Krest C. M., Onderko E. L., Silakov A., Calixto J. C., Behan R. K., Green M. T. (2013). Iron­(IV)­Hydroxide
pK­(a) and the Role of Thiolate Ligation in C-H Bond Activation by
Cytochrome P450. Science.

[118] Jorgensen W. L., Chandrasekhar J., Madura J. D., Impey R. W., Klein M. L. (1983). Comparison of Simple
Potential Functions for Simulating
Liquid Water. J. Chem. Phys..

[119] Case D. A., Cheatham T. E., Darden T., Gohlke H., Luo R., Merz K. M., Onufriev A., Simmerling C., Wang B., Woods R. J. (2005). The Amber Biomolecular
Simulation
Programs. J. Comput. Chem..

[120] Ryckaert J. P., Ciccotti G., Berendsen H. J. C. (1977). Numerical
Integration of the Cartesian Equations of Motion of a System with
Constraints: Molecular Dynamics of n-Alkanes. J. Comput. Phys..

[121] Wu X., Brooks B. R. (2003). Self-Guided Langevin Dynamics Simulation
Method. Chem. Phys. Lett..

[122] Berendsen H. J. C., Postma J. P. M., van
Gunsteren W. F., DiNola A., Haak J. R. (1984). Molecular Dynamics
with Coupling
to an External Bath. J. Chem. Phys..

[123] Darden T., York D., Pedersen L. (1993). Particle Mesh
Ewald:
An N·log­(N) Method for Ewald Sums in Large Systems. J. Chem. Phys..

[124] Ryde U. (1996). The Coordination of the Catalytic
Zinc in Alcohol Dehydrogenase Studied
by Combined Quantum-Chemical and Molecular Mechanics Calculations. J. Comput. Aided. Mol. Des..

[125] Ryde U., Olsson M. H. M. (2001). Structure, Strain,
and Reorganization
Energy of Blue Copper Models in the Protein. Int. J. Quantum Chem..

[126] Von Arnim M., Ahlrichs R. (1998). Performance of Parallel
TURBOMOLE
for Density Functional Calculations. J. Comput.
Chem..

[127] Furche F., Ahlrichs R., Hättig C., Klopper W., Sierka M., Weigend F. (2014). Turbomole. WIREs Comput. Mol. Sci..

[128] Becke A. D. (1993). Density-Functional Thermochemistry.
III. The Role of
Exact Exchange. J. Chem. Phys..

[129] Becke A.
D. (1988). Density-Functional Exchange-Energy
Approximation With
Correct Asymptotic-Behavior. Phys. Rev. A.

[130] Lee C., Yang W., Parr R. G. (1988). Development of the Colle-Salvetti
Correlation-Energy Formula into a Functional of the Electron Density. Phys. Rev. B.

[131] Weigend F., Ahlrichs R. (2005). Balanced Basis Sets of Split Valence,
Triple Zeta Valence and Quadruple Zeta Valence Quality for H to Rn:
Design and Assessment of Accuracy. Phys. Chem.
Chem. Phys..

[132] Lehtola S. (2021). Straightforward
and Accurate Automatic Auxiliary Basis
Set Generation for Molecular Calculations with Atomic Orbital Basis
Sets. J. Chem. Theory Comput..

[133] Eichkorn K., Treutler O., Öhm H., Häser M., Ahlrichs R. (1995). Auxiliary Basis Sets to Approximate
Coulomb Potentials. Chem. Phys. Lett..

[134] Eichkorn K., Weigend F., Treutler O., Ahlrichs R. (1997). Auxiliary
Basis Sets for Main Row Atoms and Transition Metals and Their Use
to Approximate Coulomb Potentials. Theor. Chem.
Accounts.

[135] Schäfer A., Horn H., Ahlrichs R. (1992). Fully Optimized Contracted
Gaussian-Basis Sets for Atoms Li to Kr. J. Chem.
Phys..

[136] Weigend F. (2006). Accurate Coulomb-Fitting
Basis Sets for H to Rn. Phys. Chem. Chem. Phys..

[137] Grimme S., Antony J., Ehrlich S., Krieg H. (2010). A Consistent
and Accurate Ab Initio Parametrization of Density Functional Dispersion
Correction (DFT-D) for the 94 Elements H-Pu. J. Chem. Phys..

[138] Grimme S., Ehrlich S., Goerigk L. (2011). Effect of the Damping
Function in Dispersion Corrected Density Functional Theory. J. Comput. Chem..

[139] Maier J. A., Martinez C., Kasavajhala K., Wickstrom L., Hauser K. E., Simmerling C. (2015). Ff14SB: Improving
the Accuracy of Protein Side Chain and Backbone Parameters from Ff99SB. J. Chem. Theory Comput..

[140] Wang J., Wolf R. M., Caldwell J. W., Kollman P. A., Case D. A. (2004). Development and Testing of a General
Amber Force Field. J. Comput. Chem..

[141] Reuter N., Dejaegere A., Maigret B., Karplus M. (2000). Frontier Bonds
in QM/MM Methods: A Comparison of Different Approaches. J. Phys. Chem. A.

[142] Hu L., Söderhjelm P., Ryde U. (2011). On the Convergence
of QM/MM Energies. J. Chem. Theory Comput..

[143] Cao L., Ryde U. (2018). On the Difference Between
Additive and Subtractive
QM/MM Calculations. Front. Chem..

[144] Rydberg P., Sigfridsson E., Ryde U. (2004). On the Role of the
Axial Ligand in Heme Proteins: A Theoretical Study. J. Biol. Inorg. Chem..

[145] Noodleman L. (1981). Valence Bond Description of Antiferromagnetic
Coupling
in Transition Metal Dimers. J. Chem. Phys..

[146] Noodleman L., Lovell T., Liu T., Himo F., Torres R. A. (2002). Insights into Properties and Energetics
of Iron-Sulfur
Proteins from Simple Clusters to Nitrogenase. Curr. Opin. Chem. Biol..

[147] Szilagyi R. K., Winslow M. A. (2006). On the Accuracy of Density Functional
Theory for Iron—Sulfur Clusters. J. Comput.
Chem..

[148] Greco C., Fantucci P., Ryde U., de Gioia L. (2011). Fast Generation
of Broken-Symmetry States in a Large System Including Multiple Iron-Sulfur
Assemblies: Investigation of QM/MM Energies, Clusters Charges, and
Spin Populations. Int. J. Quantum Chem..

[149] Hu L., Söderhjelm P., Ryde U. (2013). Accurate Reaction Energies
in Proteins Obtained by Combining QM/MM and Large QM Calculations. J. Chem. Theory Comput..

[150] Klamt A., Schüürmann G. (1993). COSMO: A New
Approach
to Dielectric Screening in Solvents with Explicit Expressions for
the Screening Energy and Its Gradient. J. Chem.
Soc. Perkin Trans. 2.

[151] Schäfer A., Klamt A., Sattel D., Lohrenz J. C. W., Eckert F. (2000). COSMO Implementation in TURBOMOLE: Extension of an
Efficient Quantum Chemical Code towards Liquid Systems. Phys. Chem. Chem. Phys..

[152] Klamt A., Jonas V., Bürger T., Lohrenz J. C. W. (1998). Refinement and Parametrization of COSMO-RS. J. Phys. Chem. A.

[153] Sigfridsson E., Ryde U. (1998). Comparison of Methods
for Deriving
Atomic Charges from the Electrostatic Potential and Moments. J. Comput. Chem..

[154] Ullmann G. M., Knapp E. W. (1999). Electrostatic Models
for Computing
Protonation and Redox Equilibria in Proteins. Eur. Biophys. J..

[155] Warshel A., Dryga A. (2011). Simulating Electrostatic Energies
in Proteins: Perspectives and Some Recent Studies of pKa’s,
Redox, and Other Crucial Functional Properties. Proteins Struct. Funct. Bioinform..

[156] Cao L., Caldararu O., Ryde U. (2017). Protonation States of Homocitrate
and Nearby Residues in Nitrogenase Studied by Computational Methods
and Quantum Refinement. J. Phys. Chem. B.

[157] Kelly C. P., Cramer C. J., Truhlar D. G. (2006). Aqueous Solvation
Free Energies of Ions and Ion-Water Clusters Based on an Accurate
Value for the Absolute Aqueous Solvation Free Energy of the Proton. J. Phys. Chem. B.

[158] Stier M., Kästner J. (2026). Reliable Redox-Potential
Simulations
of Proteins. J. Chem. Theory Comput..

[159] Dehabadi M. H., Irani M., Ryde U. (2025). Predicting Reduction
Potentials of Blue Copper Proteins Using Quantum Mechanical Calculations. Inorg. Chem..

[160] Jafari S., Tavares Santos Y. A., Bergmann J., Irani M., Ryde U. (2022). Benchmark Study of
Redox Potential Calculations for Iron-Sulfur Clusters
in Proteins. Inorg. Chem..

[161] Neese F. (2025). Software Update: The ORCA Program
System—Version 6.0. WIREs Comput. Mol.
Sci..

[162] Neese F. (2012). The ORCA Program System. WIREs Comput. Mol.
Sci..

[163] Neese F., Wennmohs F., Becker U., Riplinger C. (2020). The ORCA Quantum
Chemistry Program Package. J. Chem. Phys..

[164] Onufriev A., Bashford D., Case D. A. (2004). Exploring Protein
Native States and Large-Scale Conformational Changes with a Modified
Generalized Born Model. Proteins Struct. Funct.
Bioinforma..

[165] Besler B. H., Merz K. M., Kollman P. A. (1990). Atomic Charges Derived
from Semiempirical Methods. J. Comput. Chem..

[166] Furness J. W., Kaplan A. D., Ning J., Perdew J. P., Sun J. (2020). Accurate and
Numerically Efficient r^2^SCAN Meta-Generalized
Gradient Approximation. J. Phys. Chem. Lett..

[167] Caldeweyher E., Ehlert S., Hansen A., Neugebauer H., Spicher S., Bannwarth C., Grimme S. (2019). A Generally Applicable
Atomic-Charge Dependent London Dispersion Correction. J. Chem. Phys..

[168] Caldeweyher E., Mewes J.-M., Ehlert S., Grimme S. (2020). Extension
and Evaluation of the D4 London-Dispersion Model for Periodic Systems. Phys. Chem. Chem. Phys..

[169] Cao L., Ryde U. (2020). What Is the Structure
of the E_4_ Intermediate
in Nitrogenase?. J. Chem. Theory Comput..

[170] Zhao Y., Truhlar D. G. (2008). The M06 Suite of Density Functionals
for Main Group Thermochemistry, Thermochemical Kinetics, Noncovalent
Interactions, Excited States, and Transition Elements: Two New Functionals
and Systematic Testing of Four M06-Class Functionals and 12 Other
Function. Theor. Chem. Acc..

[171] Mardirossian N., Head-Gordon M. (2016). ωB97M-V:
A Combinatorially
Optimized, Range-Separated Hybrid, Meta-GGA Density Functional with
VV10 Nonlocal Correlation. J. Chem. Phys..

[172] Eriksson A. E., Jones T. A., Liljas A. (1988). Refined Structure
of
Human Carbonic Anhydrase II at 2.0 Å Resolution. Proteins Struct. Funct. Bioinform..

[173] Jiang H., Svensson O. K. G., Ryde U. (2023). Quantum Mechanical
Calculations of Redox Potentials of the Metal Clusters in Nitrogenase. Molecules.

