# Supplementary material for: Benchmark Study for Calculations of pK a Values of Metal Ligands in Proteins
Source: J Chem Theory Comput. 2026 Jul 6;22(14):7420–35. doi: 10.1021/acs.jctc.6c00884 (PMC13421986; doi:10.1021/acs.jctc.6c00884)
Supplement: Supplementary file 2 [file ct6c00884_si_002.pdf]

Supporting Information

Benchmark study for calculations of pK_a values of metal ligands in proteins

Maryam Haji Dehabadi,[1] *Mehdi Irani,*[1*] *Sonia Jafari,*[1] *& Ulf Ryde*[2*]

[1] Department of Chemistry, University of Kurdistan, Sanandaj 66177-15175, Iran

[2] Division of Computational Chemistry, Lund University, Chemical Centre, P. O. Box 124, SE-221 00 Lund, Sweden

Correspondence to
Mehdi Irani, m.irani@uok.ac.ir or
Ulf Ryde, Ulf.Ryde@compchem.lu.se, Tel: +46 – 46 2224502.

https://doi.org/10.1021/acs.jctc.6c00884

2026-07-05

1. Description of the Studied Systems

1.1 Alcohol Dehydrogenase

Alcohol dehydrogenase (EC 1.1.1.1; ADH) catalyzes the reversible oxidation of alcohols to aldehydes or ketones, accompanied by the simultaneous reduction of nicotinamide adenine dinucleotide (NAD^+) to NADH (cf. Scheme S1).[1]

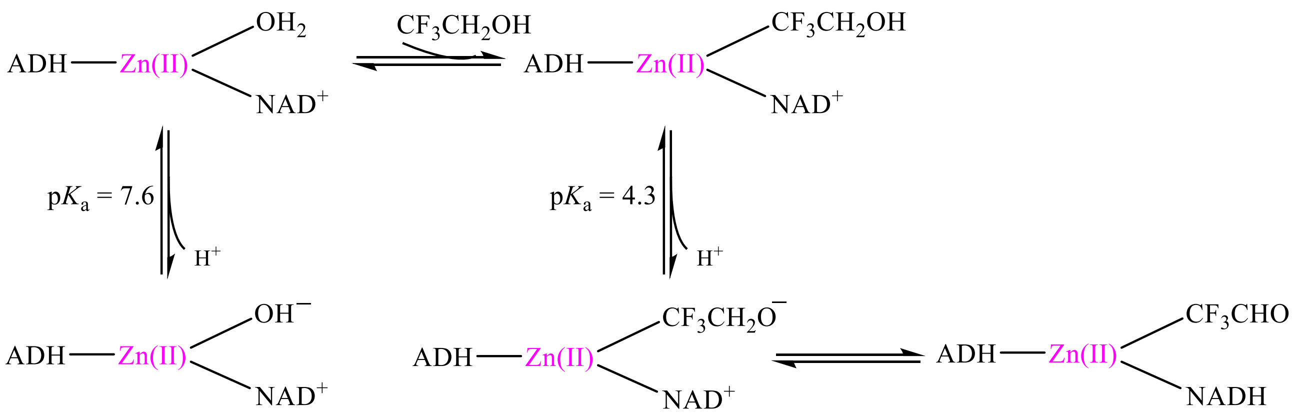

Scheme S1. Reaction mechanism proposed by Kvassman and Pettersson[2] for trifluoroethanol binding and proton transfer during liver alcohol dehydrogenase catalytic reaction.

The major form of this enzyme in mammals is a dimer, comprised of two identical subunits, each containing two bound zinc ions. Each subunit is divided into two domains by a cleft with a deep pocket. This pocket accommodates the substrate and the nicotinamide part of NAD^+ (cf. Figure S1a). The smaller domain (shown as a cyan cartoon in Figure S1b) comprises residues 178 to 318 and contains most of the residues participating in the coenzyme binding.[1] The larger domain (green cartoon in Figure S1b) provides ligands to the protein-bound zinc ions and contains most of the residues controlling substrate binding and the catalytic activity. However, only one of the zinc ions is catalytically active (pink sphere in Figure S1).[3] The second zinc ion (yellow sphere in Figure S1) plays a structural role[4] and does not participate in the catalytic reaction. The catalytic zinc ion is positioned at the bottom of the domain-separating cleft, about 20 Å from the protein surface, and is bound by two cysteines (Cys-46 and 174), one histidine (His-67), and a substrate or solvent molecule.[5] The structural zinc ion is located about 20 Å from the catalytic metal site in a polypeptide lobe that projects out of the catalytic domain. It is tetrahedrally coordinated by sulfur atoms from four cysteine residues (Cys-97, 100, 103, and 111).

The coenzyme of ADH has an adenosine moiety that is bound to the cavity by both hydrophobic interactions[3,6] and hydrogen bonds with Asp-223 and Lys-228. Both hydroxyl groups of the ribose moiety on the adenine side participate in interactions with Asp-223. The coenzyme's pyrophosphate group is close to the edge of the domain that separates the cleft and bends over the pleated-sheet structure of the domain that binds the coenzyme. Hydrogen bonds between the phosphate oxygen atoms and the main-chain nitrogen atoms of Gly-202 and Val-203 stabilize this conformation of the pyrophosphate

moiety. It also forms interactions with the side chains of Arg-47, Arg-369, and Lys-228. The nicotinamide ribose part of the coenzyme is tightly held in the thin cleft between the two subunit domains. One of the nicotinamide-side ribose hydroxyl groups forms hydrogen bonds with the side chains of Ser-48 and His-51 (Scheme S2). This hydrogen-bond network plays a crucial role in the catalytic proton transfer from the substrate to the solution, as depicted in Scheme S2. We included these groups (Ser-48 and His-51) in the intermediate QM system.

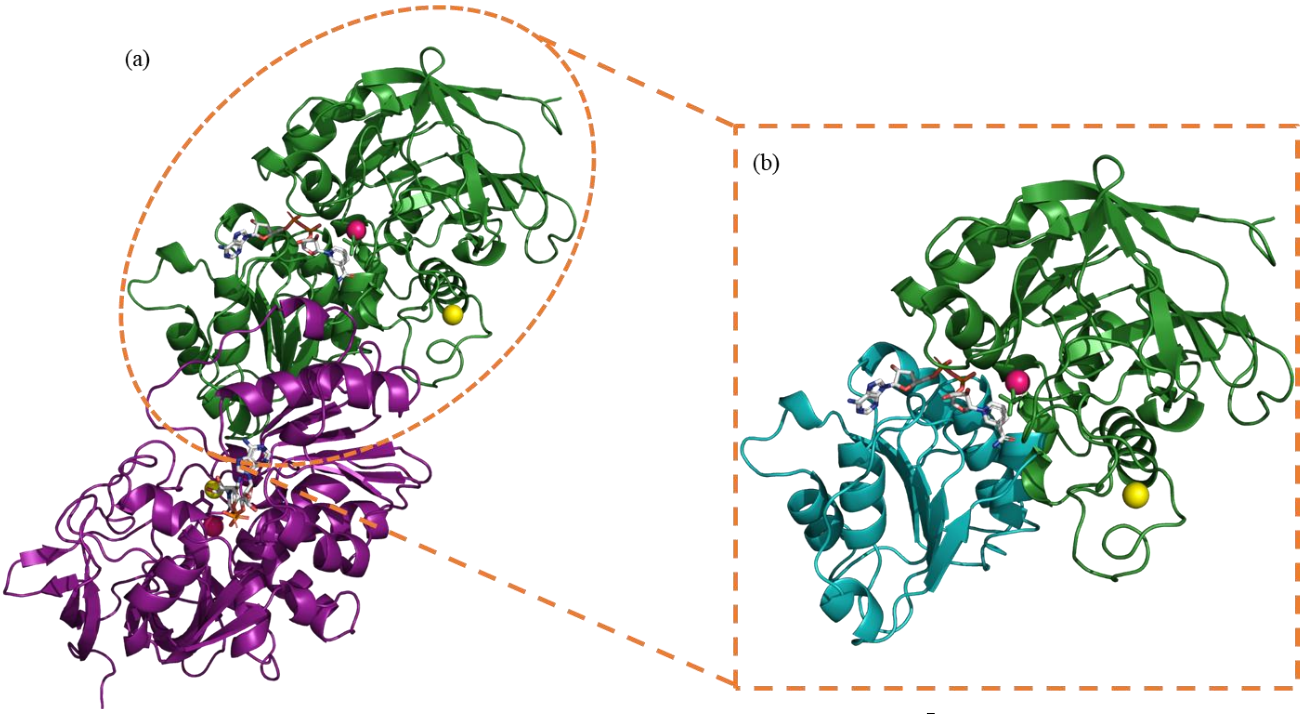

Figure S1. (a) Representation of the liver ADH dimer (PDB ID: 1AXE)[7]. Two chains of the dimer are represented in green and purple. (b) The subunits in one monomer of the liver ADH. The NAD^+ coenzyme and the zinc ions are represented in sticks and spheres, respectively. Residues 178–318 are shown in cyan, the catalytic zinc ion is shown in pink, and the structural zinc ion is shown in yellow.

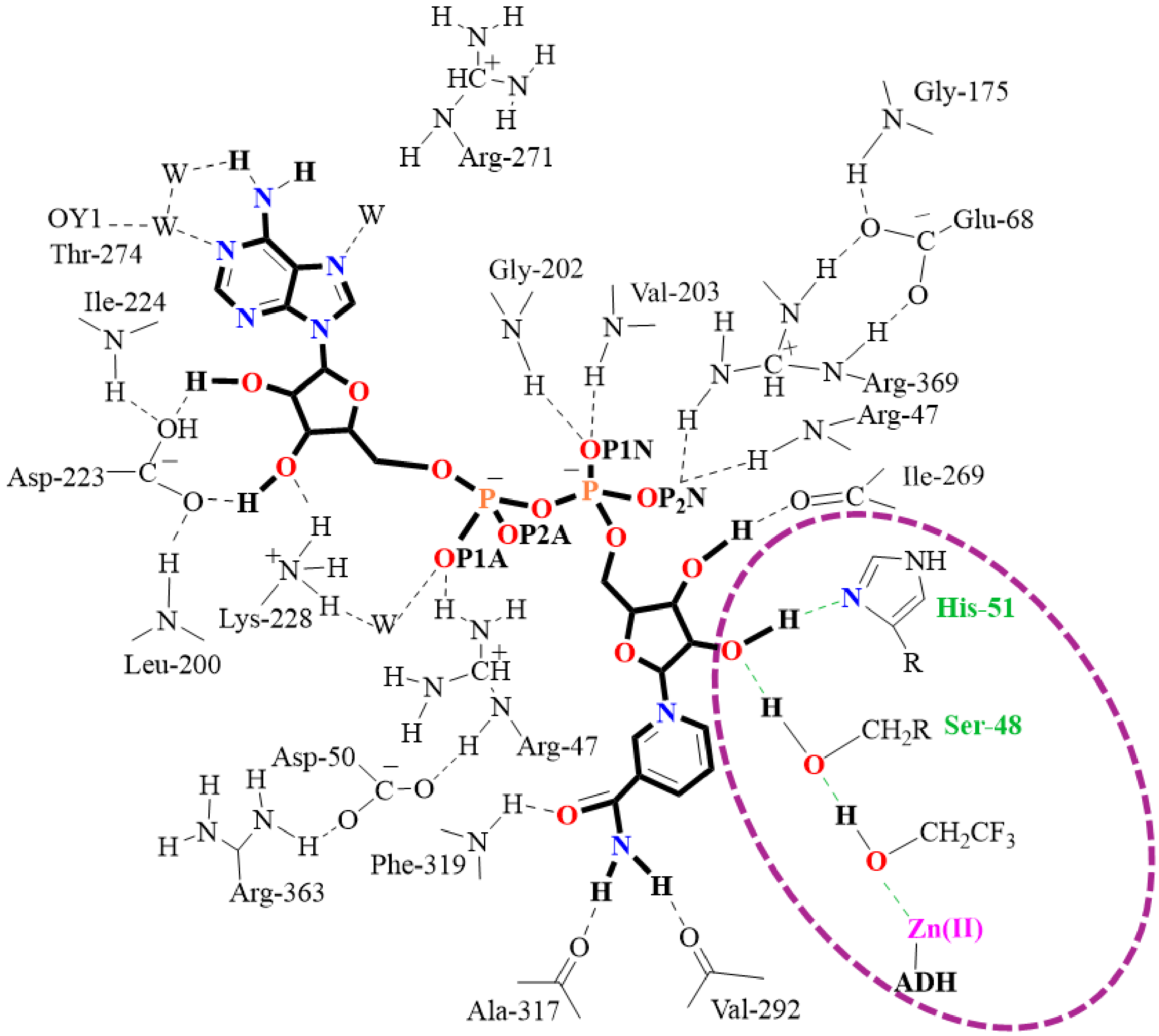

Scheme S2. Schematic drawing of the hydrogen-bond interactions between the coenzyme and liver alcohol dehydrogenase.[8] The circle indicates residues involved in the proposed mechanism for the catalytic proton-transfer step, which involves the ionization of the zinc-bound alcohol and the release of a proton to solution via a system of hydrogen bonds.

We investigate four acid-base reactions of ADH for which experimental pK_a values are available. The reactions differ by the substrate and the cofactor (Scheme S3). The first reaction is the deprotonation of trifluoroethanol (CF_3CH_2OH) when NAD^+ is bound to ADH (pK_a = 4.3).[2] The second and third reactions involve the deprotonation of a Zn-bound water molecule with either NAD^+ (pK_a = 7.6)[9–11] or NADH (pK_a = 11.2)[12] bound to ADH, respectively. The fourth reaction involves the deprotonation of the Zn-bound water molecule in the coenzyme-free form of ADH (pK_a = 9.2).[9]

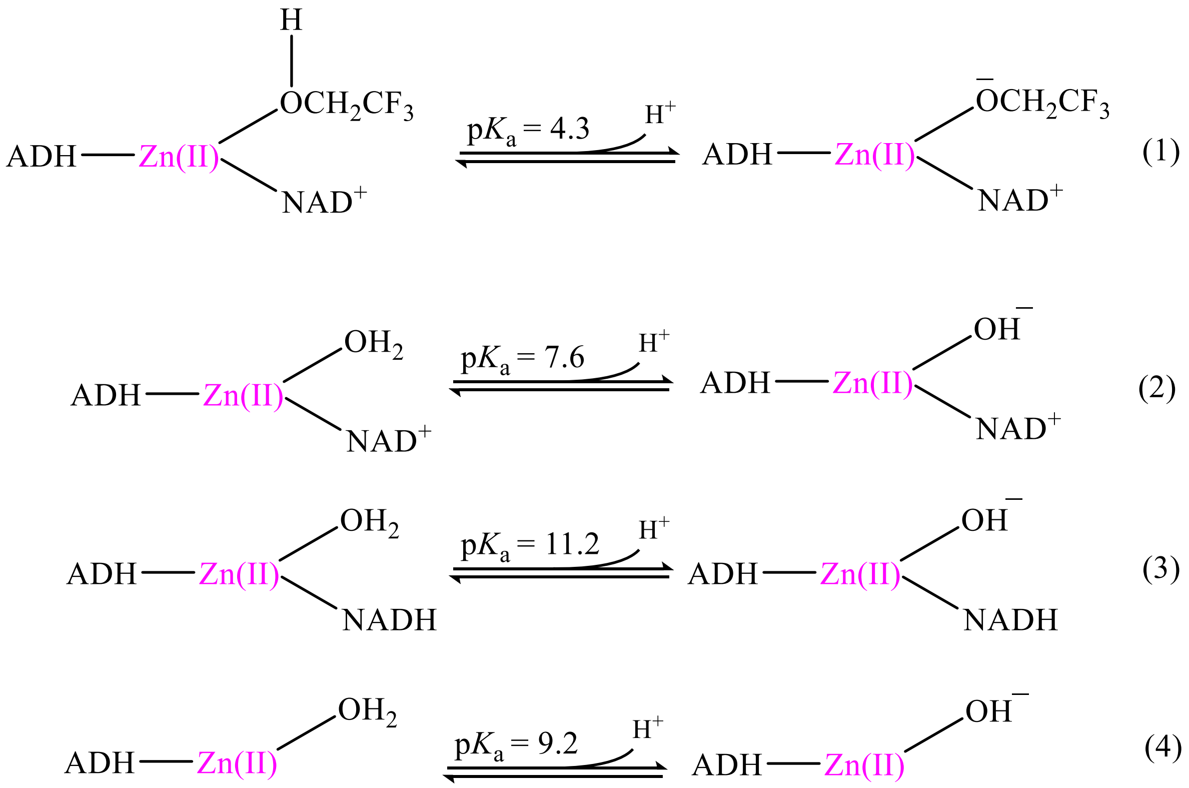

Scheme S3. The reactions studied for the ADH enzyme in different states, depending on the substrate and the coenzyme. Reaction numbers assigned for each acid-base reaction are shown at the end of each reaction.

1.2 Thioredoxin-like Ferredoxins

Thioredoxin-like [2Fe–2S] ferredoxin (TLF) is found in some bacteria. While TLF is not ubiquitous, it does occur across all bacterial lineages, most frequently in proteobacteria and cyanobacteria.[13] The first publication about TLF from *C. pasteurianum* reported that the protein is more abundant in N_2-fixing cells[14] and this observation was confirmed in subsequent investigations.[15–17] However, an inspection of the genomic context of other TLF-encoding genes showed that, while they frequently occur in the vicinity of nitrogen fixation genes,[18] they are also present in bacteria that cannot fix dinitrogen, e.g., *Aquifex aeolicus*.[19,20] Still, the data point to a role of TLF in nitrogen metabolism. The overall stability of TLF and the [2Fe–2S] cluster might suggest an electron-carrier function, which has been investigated in *C. pasteurianum*. TLF does not donate electrons to, or accept them from hydrogenase.[21,22] It is a very inefficient oxidant of pyruvate–ferredoxin oxidoreductase.[21] It binds to the nitrogenase molybdenum-iron (MoFe) protein without electron transfer.[23,24] These negative results suggest that TLF may not be an electron carrier, a possibility supported by structural features (a dimer with protruding loops) suggestive of a regulatory function. On the other hand, electron transfer, or at least the regulation of electron transfer, is the most likely function of TLF domains occurring in complex I[25] or hydrogenases.[26]

TLF is a dimer (**Figure S2**a), consistent with previous biochemical data.[15,16,20,27] The structure of this protein is distinct from that of other [2Fe–2S] proteins and other Fe–S proteins as a whole.[28] It

consists of five β-strands, two long α-helices, and additional short α- or 3_{10} helices.[29] It bears striking similarity to the overall α/β architecture of thioredoxin.[30] The main difference is the presence in thioredoxin of α-helices on both sides of the central β-sheet, while in TLF, the missing helices on one side expose the β-sheet and allow it to form a dimer interface. The TLF fold is one among at least six or seven distinct protein folds accommodating [2Fe–2S] clusters with four cysteine ligands (**Figure S2**b).[28] Other proteins contain [2Fe–2S] clusters having one (MitoNEET)[31] or two (Rieske proteins)[32–34] cysteines replaced by histidines, or an arginine replacing one cysteine (biotin synthase).[35] In the first TLF structure at medium resolution, the [2Fe–2S] clusters were refined with structural restraints corresponding to the canonical geometry, and no diverging structural details were detected.[29] Subsequent structures of the wild type and variants (C55S and C59S) were all obtained at resolutions allowing refinement without restraints on the metal site geometry.[36] The crystal structure of the *Aquifex aeolicus* protein revealed positions of charged or polar residues in the vicinity of the [2Fe–2S] cluster, in particular, Arg-13, which is involved in hydrogen bonds with the SG atoms of the Cys residues binding to Fe, and Gln-11, which is ~6 Å away from the nearest Fe).[36]

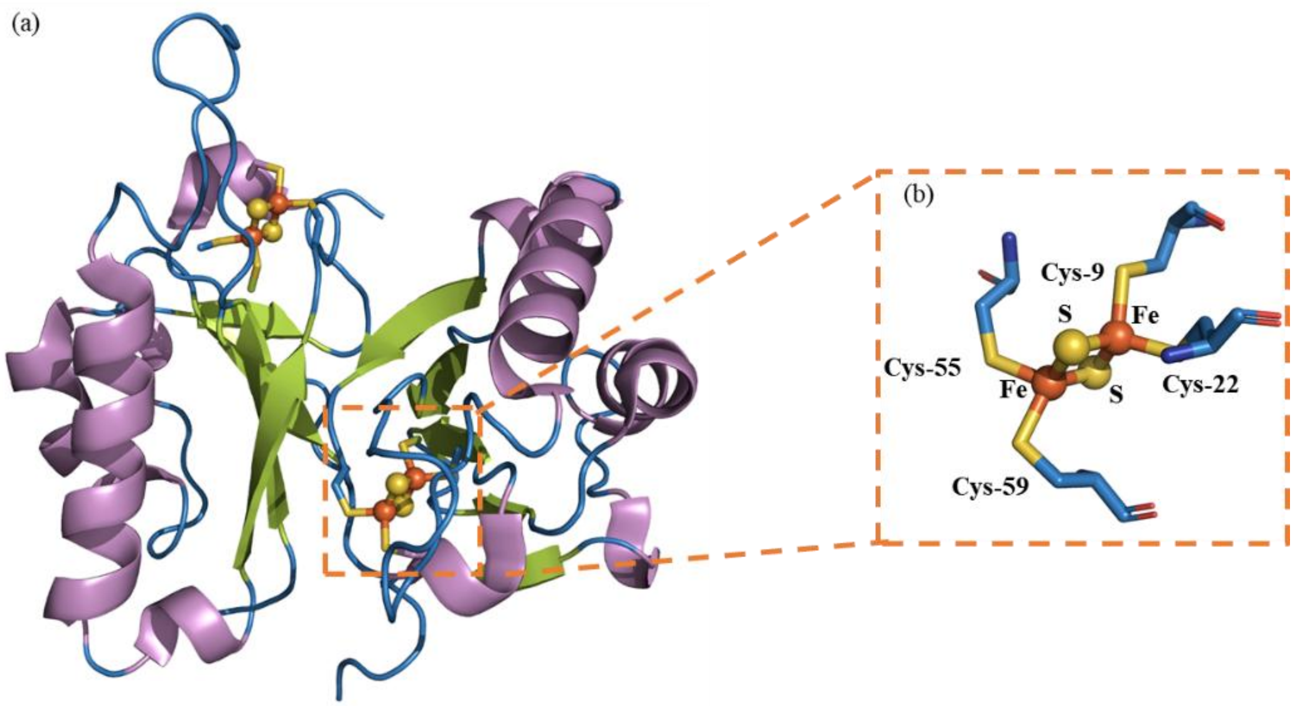

Figure S2. View of the *Aquifex aeolicus* TLF dimer (PDB ID: 1F37)[29]. (a) A cartoon representation of the general structure. (b) Residues involved in the redox reaction. Atoms within the [2Fe–2S] cluster are shown as red and yellow spheres.

A spectroscopic and redox study on the [2Fe−2S] centers of TLF in the wild-type and oxidized Cys-to-Ser variants (C55S and C59S) of *Aquifex aeolicus* ferredoxin 4 (*Aae*Fd4) demonstrated that a single protonation event occurs with a pK_a of 9.0 and 8.3 for the C55S and C59S variants, respectively.[37] As the protonated serine is expected to be a weak cluster ligand and the reducible Fe site of the cluster is solvent exposed,[36] it seems likely that serine is replaced as a Fe ligand by water or hydroxide when

the samples are reduced at pH values below the pK_a (Scheme S4).[37] In this work, we study the acid-base reactions of two mutant forms of TLF in *Aquifex aeolicus*: C55S and C59S (reactions 5 and 6 in **Scheme S4**), for which experimental pK_a values are available.

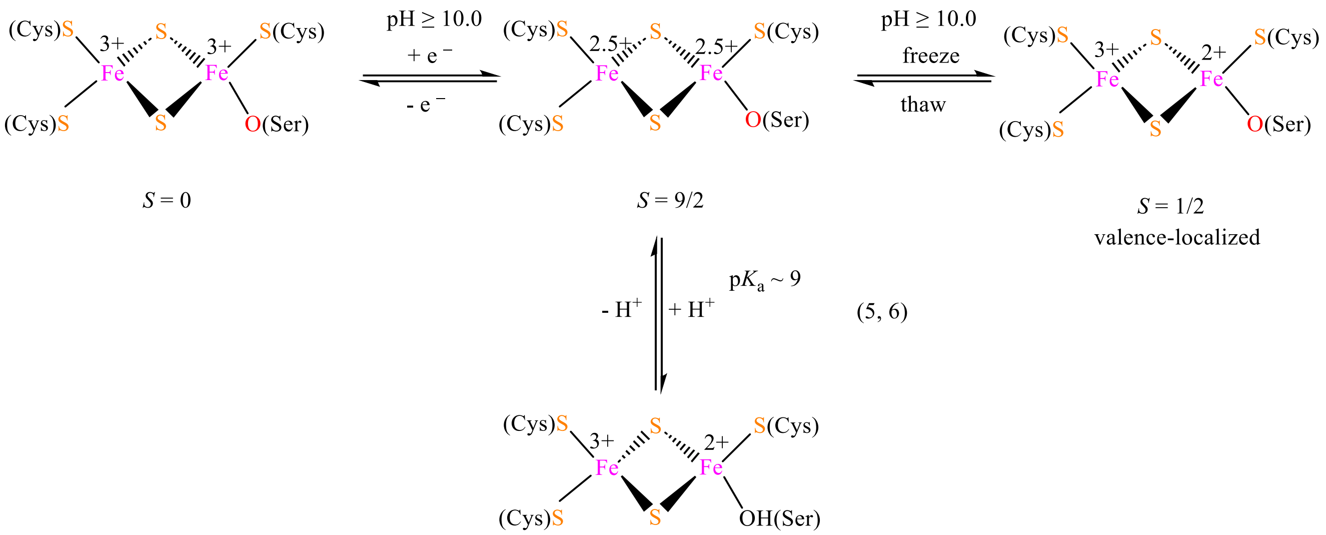

Scheme S4. Schematic summary of the proposed ligation, spin states, and redox properties of the $[2Fe–2S]^{2+}$ centers in the Cys-to-Ser variants of *Aae*Fd4 as a function of pH.[37] Reaction numbers assigned for each acid-base reaction are shown at the end of each reaction. Reactions 5 and 6 are identical but occur in different enzyme mutants (C55S and C59S, respectively).

1.3 Carbonic Anhydrase

Carbonic anhydrase (EC 4.2.1.1; CA) catalyzes the interconversion of carbon dioxide and bicarbonate. Among its various isoforms, human carbonic anhydrase II (HCAII), one of the two erythrocytic forms in humans, is one of the fastest enzymes known, with a k_{cat}, of 1×10^6 s^{-1} and a K_m of 8.3 mM for hydration of CO_2.[38] The corresponding parameters for the reverse reaction are 6×10^5 s^{-1} and 32 mM, respectively. The enzyme can also hydrate aldehydes[39] and hydrolyze esters.[40]

HCAII consists of a single polypeptide chain of 259 amino acid residues and a zinc ion (**Figure S3**a).[41,42] The enzymatic reaction occurs at the zinc coordination center.[43] The zinc ion is tetrahedrally coordinated to three histidine residues (His-94, 96, and 119) and a water/hydroxide (**Figure S3**b)[44,45] with a pK_a of ~7.[38]

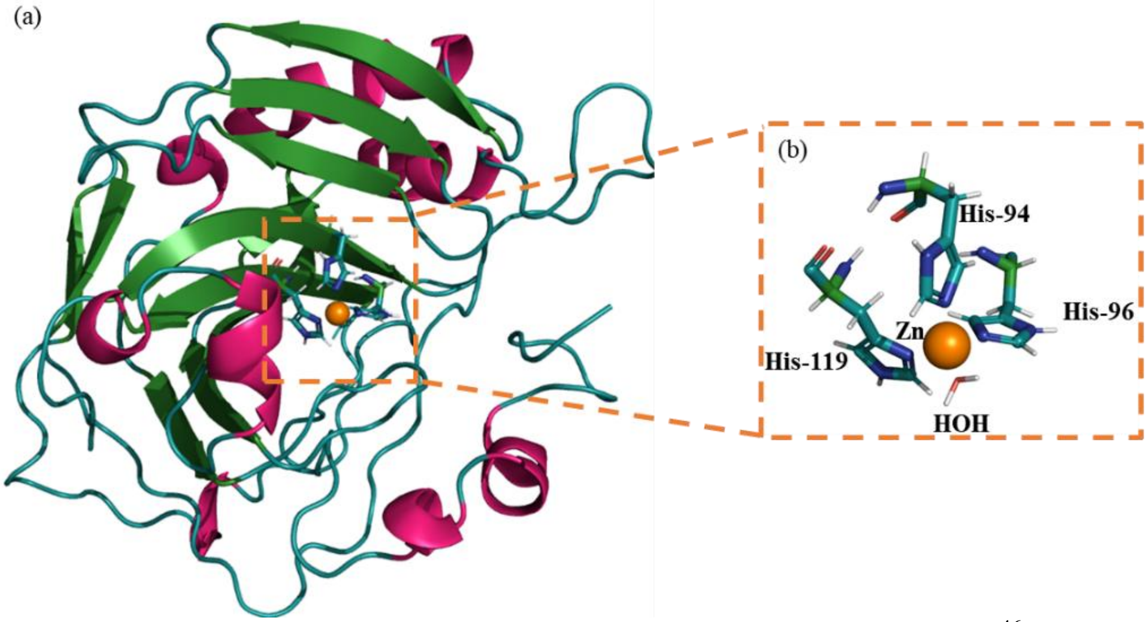

Figure S3. (a) Cartoon representation of human carbonic anhydrase (PDB ID; 2ILI).[46] (b) Details of the Zn active site.

The CA mechanism can be divided into two main steps, as depicted in **Scheme S5**.[47–49] In the hydration direction, the first step is binding a CO_2 molecule in a hydrophobic region of the active site, followed by the nucleophilic attack by a Zn-bound hydroxyl ion on the substrate carbon to form bicarbonate (HCO_3^-). HCO_3^- is then displaced from the active site by a water molecule. The second part of the reaction is rate-limiting and involves the transport of a proton from the Zn-bound water molecule to the side chain of His-64, which is located on the edge of the active site, from which the proton is further transferred out to bulk solvent.

This mechanistic proposal is supported by both kinetic data[47] and experimental observations of several water molecules positioned between zinc-bound water and His-64.[44,45] Based on the examination of the zinc–hydroxide crystal structure of HCAII (PDB ID: 2CBA),[45] the shortest pathway between the zinc ion and His-64 involves two water molecules. Still, three water molecules could also form a bridge between the zinc ion and His-64.[50] It has been observed that His-64 occupies two conformations at different pH values: the "in" conformation points inward toward the active site, and the "out" conformation points outward away from the active site.[44] As a proton shuttle, His-64 is thought to function effectively due to its apparent flexibility.[51,52] We considered the inward conformation for our calculations, but we also performed test calculations on the "out" conformation.

Point mutations of His-64 with non-proton-transferring amino acid residues reduce the rate of catalysis by 20-fold but do not completely eliminate enzyme activity.[50] The small activity of the mutant protein shows that there may be other proton-transfer pathways than the one involving His-64.[53,54] Another mechanistic proposal involves the hydrogen-bond network involving Zn-OH_2, Thr-199, and Glu-106 in removing the proton and generating the nucleophilic Zn-OH. Although this mechanism is supported by the observation that this hydrogen-bond chain is present in all known CAs,[47,55,56] it is

unlikely because it requires the elevation of the pK_a of Glu-106 (from 4 to >7).[57]

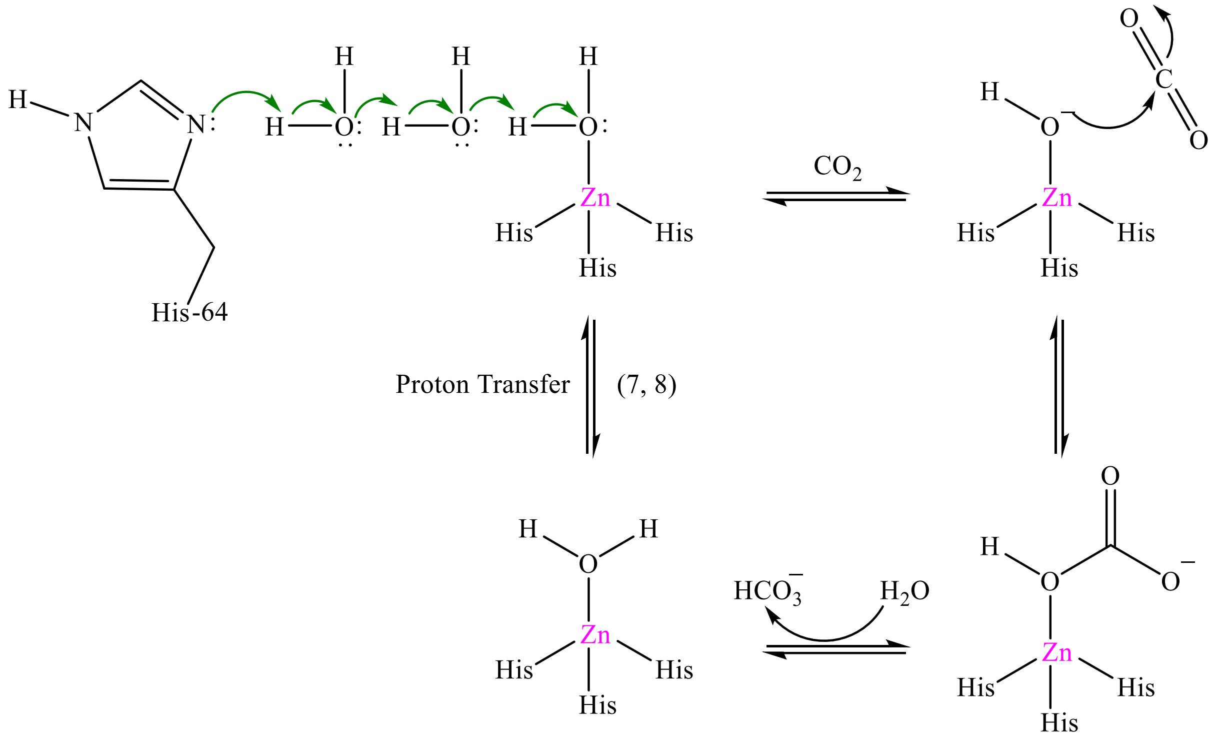

Scheme S5. The acid-base reaction mechanism of CA. Reaction numbers assigned for each acid-base reaction are shown at the end of each reaction. Reactions 7 and 8 are the same but are in different enzyme variants (WT and E106Q).

1.4 Myoglobin

Myoglobin (Mb) is a protein found primarily in the cardiac and skeletal muscles of vertebrates. The primary function of Mb is to supply oxygen to the muscle. Mb serves as a buffer of intracellular oxygen concentrations and as an oxygen reservoir.[58] During muscle activity, Mb becomes desaturated, increasing the oxygen diffusion gradient from the capillaries to the cytoplasm.[59] In addition to its role in oxygen storage and transport, Mb has been shown to possess enzymatic functions. It contributes to the decomposition of bioactive nitric oxide (NO) into nitrate.[59] The removal of nitric oxide enhances mitochondrial respiration, because nitric oxide reversibly inhibits cytochrome oxidase. Furthermore, Mb plays a role in the removal of reactive oxygen species (ROS) and has been reported to exhibit peroxidase-like activity, catalyzing the H_2O_2-dependent oxidation of various substrates (cf. **Scheme S6**).

Structurally, Mb is a monomeric heme protein, consisting of a single chain of 153 amino acids and a non-covalently bound heme moiety. The structure is compact and globular, consisting of five helices assigned the letters A–E (cf. **Figure S4**a).

The active site of Mb is situated in a hydrophobic pocket near the center of the protein and consists of a prosthetic heme group with a Fe(II) ion in the center of a porphyrin ring, coordinated by the four nitrogen atoms of the pyrrole rings (cf. **Figure S4**b). The ion is also bound to the NE2 atom of the conserved proximal histidine (His-93) residue. The sixth coordination site, completing the octahedral coordination around the iron ion, is empty or occupied by O_2. Another His residue (the distal

His-64) forms a hydrogen bond to the bound O_2 molecule, thereby stabilizing the binding and favoring O_2 compared to other competing diatomic gases, e.g., CO and NO.[60]

Significant effort has been devoted to investigating the reaction cycles of these heme proteins. The reaction cycle of myoglobin starts with the resting Fe(III) state (cf. **Scheme S6**), which is five-coordinate with an empty coordination site (1). This state binds H_2O_2, which may initially lead to the formation of a transient Fe(IV)-hydroxide species (Fe(IV)-OH) (3). Subsequently, via proton transfer, this species evolves into compound I, characterized by Fe(IV)=O and a porphyrin radical (4). Compound I is reactive and can take up one electron and a proton to form compound II (Fe(IV)-OH) (5), which in turn returns to the resting state after the uptake of an additional electron and proton.

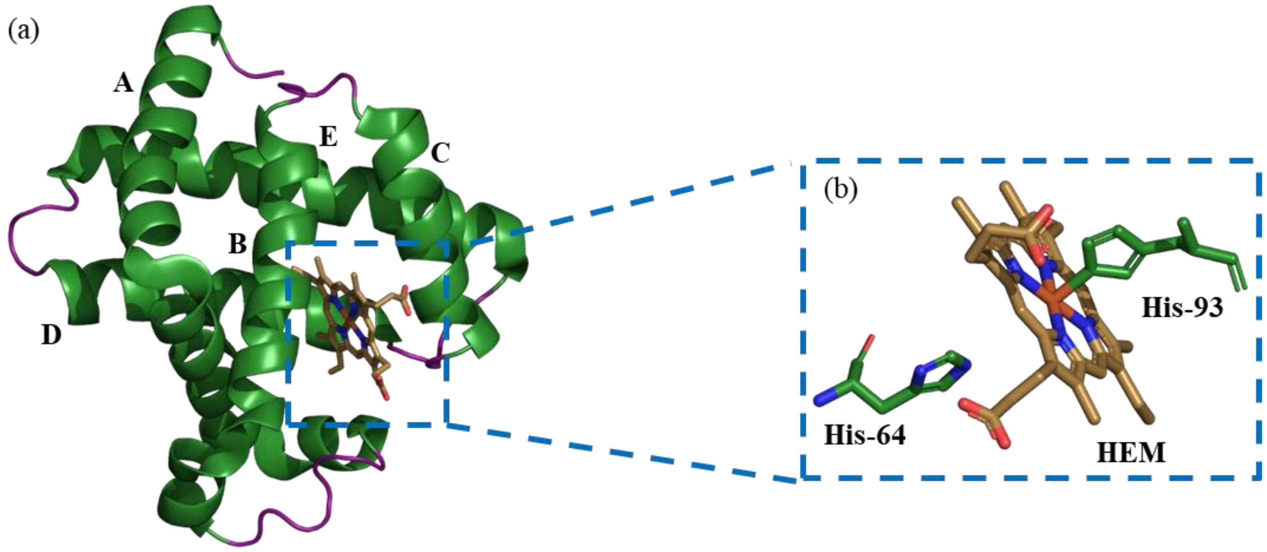

Figure S4. Crystal structure of Mb. (a) Cartoon representation of Mb (PDB ID 2V1E)[61], labeled with the corresponding α-helices. (b) The active site of Mb, including the distal His residue.

The Fe(IV)–hydroxide species, known as Mb compound II (Mb-CmpII), have long served as a model for intermediates in the catalytic cycles of heme peroxidase. Mössbauer measurements performed by Yosca et al. suggested an upper limit, $pK_a \leq 2.7$, for Mb-CmpII.[62] In our study, we estimate the pK_a value for Mb-CmpII by QM/MM calculations (cf. **Scheme S7**).

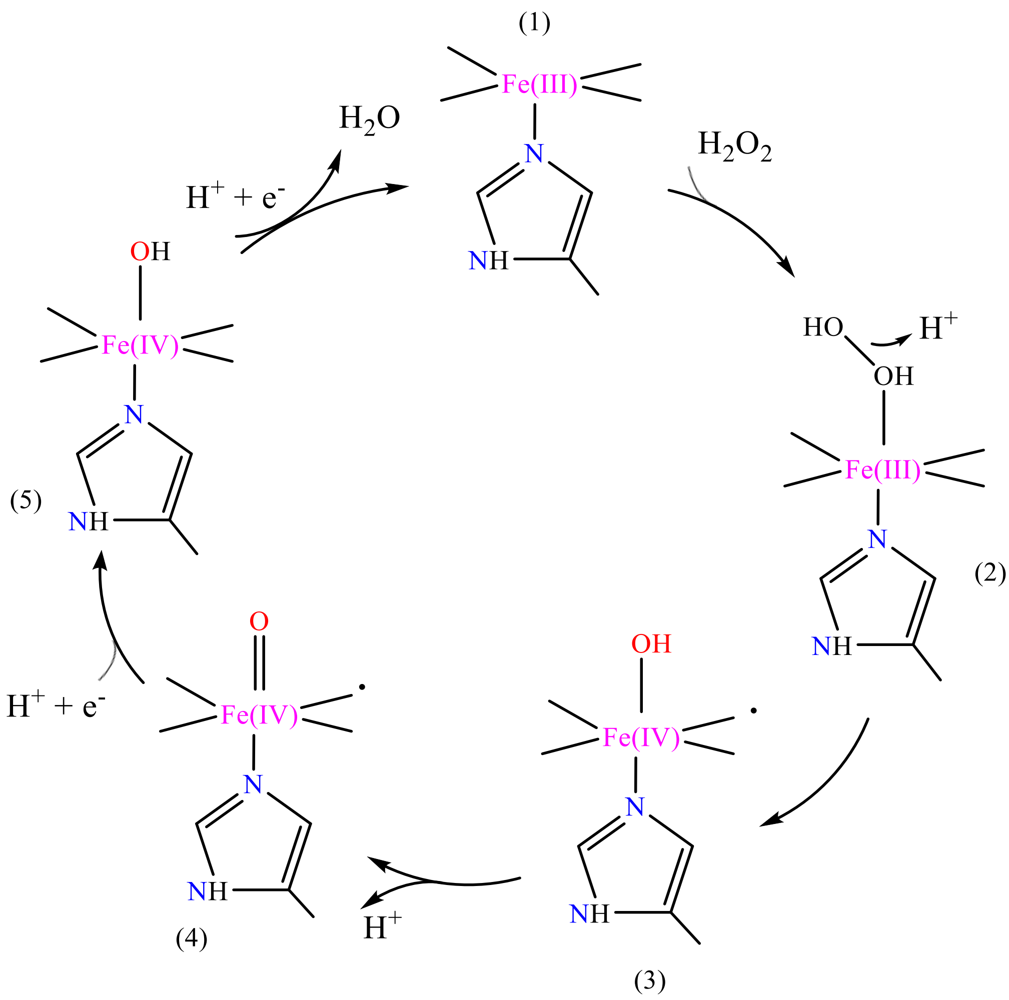

Scheme S6. Peroxidase cycle of myoglobin.

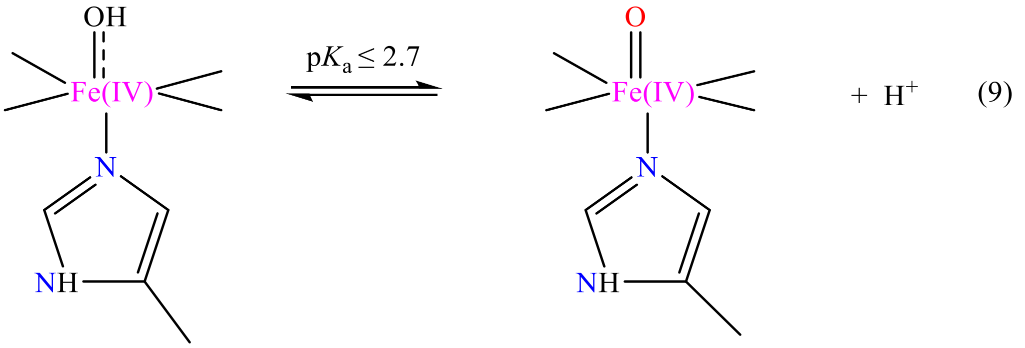

Scheme S7. The acid-base reaction studied for Mb-CmpII.

1.5 Cytochrome P450

The cytochromes P450 (EC 1.14.14.1; P450s or CYPs) are a superfamily of cysteine thiolate-ligated heme-containing monooxygenase enzymes that catalyze the oxidative transformation of a wide range of organic substrates, whose functions are crucial to both exogenous and endogenous metabolism and steroid transformation in humans and other organisms.[63]

P450 enzymes share a common overall fold and topology, despite having less than 20% sequence identity across the gene superfamily (cf. **Figure S5**a).[64] The core of the P450 enzymes is formed by a four-helix bundle comprising the D, E, I, and L-helices, with the prosthetic heme moiety

at the center of the P450 active site, sandwiched between the I- and L-helices.[65,66]

The active site of cytochrome P450 contains a heme–iron center, the same entity found in the oxygen transport and storage proteins hemoglobin and myoglobin. The iron is bound to the protein through a highly conserved cysteine thiolate ligand (cf. **Figure S5**b).

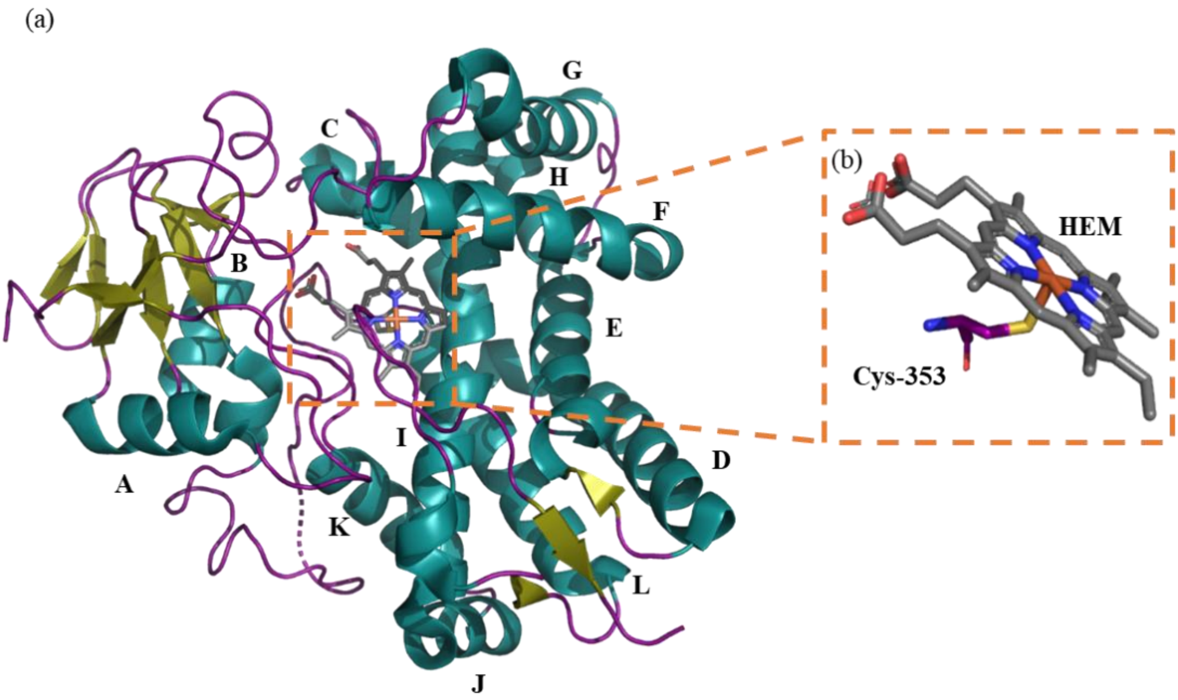

Figure S5. P450 crystal structure. (a) Cartoon representation of cytochrome P450 (PDB ID; 1S1F),[67] labeled with the corresponding α-helices. (b) The central heme moiety and cysteine residue.

The P450s utilize molecular oxygen, two electrons, and two protons to catalyze the controlled activation of inert C–H bonds.[65,68,69] The principal oxidant in these demanding transformations is thought to be a short-lived, highly reactive ferryl radical species, called compound I.[70–72]

The catalytic cycle of P450 can be described in nine steps (cf. Scheme S8).[73] The first step involves the binding of substrate to the resting low-spin ferric enzyme (1). This binding induces structural changes, which often, but not always, manifest themselves in the dissociation of the distally coordinated water molecule and the conversion of the heme from low- to high-spin (2). These substrate-induced structural changes facilitate the reduction of the ferric enzyme, allowing the delivery of the first electron and generating the ferrous substrate-bound form of the enzyme (3). Dioxygen then binds to the ferrous heme, forming what is best described as a ferric superoxide complex (4). The subsequent reduction of this species forms a ferric peroxo species (5), which is protonated at the distal oxygen to generate a ferric hydroperoxo complex (6). The delivery of an additional proton to the distal oxygen cleaves the O–O bond, yielding compound I (Fe(IV)=O and a porphyrin radical; 7) and a water molecule. Compound I then abstracts a hydrogen atom from the substrate to yield compound II (Fe(IV)–OH; 8) and a substrate radical, which rapidly recombines to yield a hydroxylated product and ferric enzyme (9). The hydroxylated product then dissociates, and water coordinates to the heme, regenerating

the resting ferric enzyme.

In this work, we study the acid-base reaction for P450 compound II (P450-CmpII) (cf. **Scheme S9**) and estimate the pK_a value for this reaction by QM/MM calculations. UV−vis and Fe Mössbauer experiments suggest that the pK_a is ~12.[74]

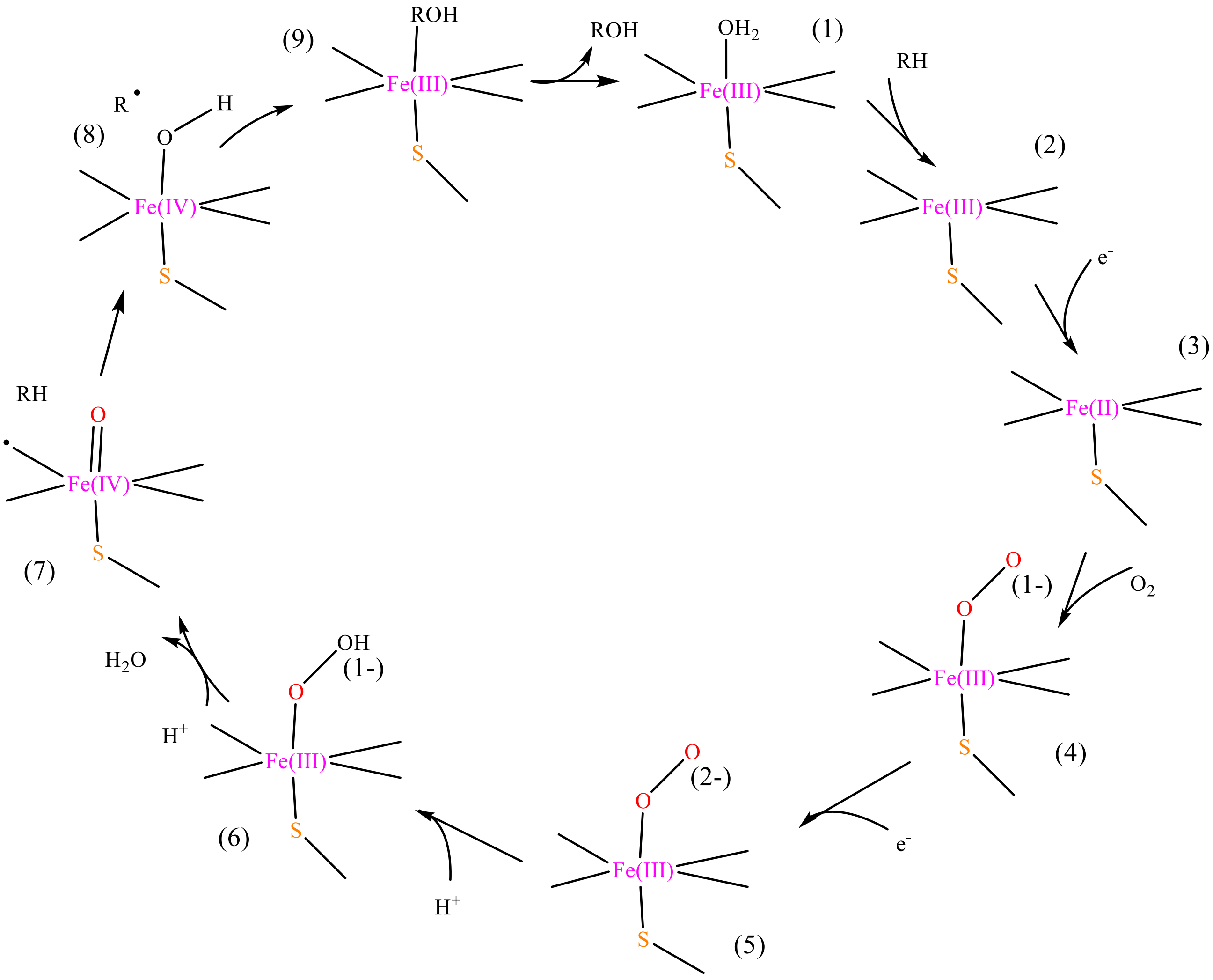

Scheme S8. P450 catalytic cycle.

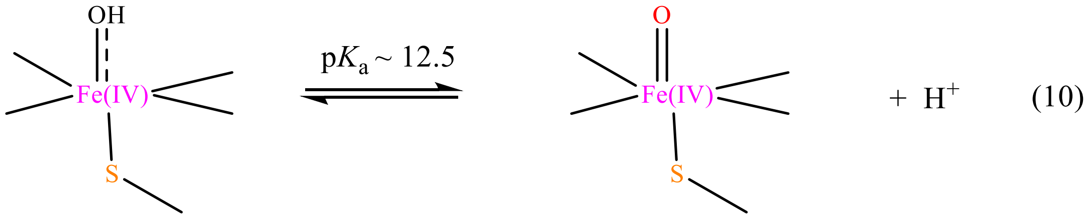

Scheme S9. The acid-base reaction studied for P450-CmpII.

1.6 Heme nitric oxide/oxygen binding (H-NOX) protein

H-NOX proteins are a diverse and versatile family of heme proteins that play important roles in various biological systems. One important function of hemoproteins is to sense gaseous diatomic molecules.[75–77] They are also involved in diverse and complex redox reactions, including electron transfer and oxygen activation.[78,79]

H-NOX proteins share a similar structure, but they exhibit distinct amino acid sequences and heme properties. They have a globular shape with a heme-binding pocket in the center (cf. **Figure S6**a). The heme-binding pocket consists of a proximal histidine residue that coordinates the heme iron and a distal tyrosine residue that stabilizes the bound oxygen or NO. Heme iron can have different oxidation states (+2 or +3) and spin states (high-spin or low-spin), which affect its affinity and specificity for various ligands. The H-NOX proteins can be classified into two groups: In NO-selective H-NOXs (e.g., VCA0720[76] and L1 H-NOX[80]), NO is bound to a five-coordinate Fe(II) complex that does not have a proximal ligand, strictly excluding oxygen as a ligand. They are similar to soluble guanylate cyclase (sGC) in forming five-coordinate NO complexes.[81] However, in O_2/NO binding H-NOXs (e.g., Tt Tar4H[76]), Fe(II) forms a six-coordinated complex. They are more similar to myoglobin and hemoglobin.[81] The selection mechanism for the discrimination between NO and O_2 by H-NOX has been thoroughly studied.[76,80,82–88] A tyrosine in the distal heme pocket of the H-NOX heme fold is necessary for stabilization of a bound O_2. Marletta and co-workers reported the crystal structure of O_2 binding to H-NOX from *Thermoanaerobacter tengcongensis* (*Tt* H-NOX).[88] Structures of a similar protein were reported by Raman and co-workers.[87] One of the significant features of the O_2-bound structure of *Tt* H-NOX is the presence of a distal pocket hydrogen-bonding network, which includes Tyr-140, Asn-74, and Trp-9 (cf. **Figure S6**b).

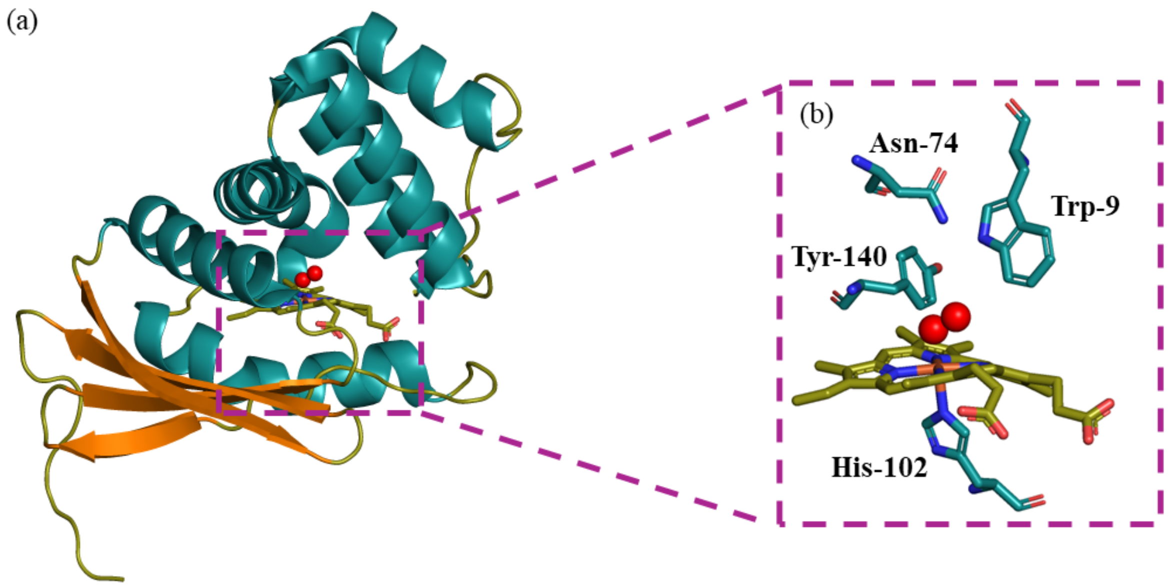

Figure S6. H-NOX(O_2) crystal structure. (a) Cartoon representation of H-NOX(O_2) (PDB ID: 1U55)[88] (b) The active-site structure and residues surrounding the O_2 ligand in a H-NOX(O2) structure.

A unifying feature of hemoproteins is the presence of a redox-active heme cofactor. The redox potentials of these proteins span an unusually wide range, from approximately +450 mV to –550 mV.[79,89,90] Such a large variation in the redox potentials has been attributed to various aspects of protein structure. For example, in human myoglobin and cytochromes, the hydrophobic residues in the heme-

binding pocket are a crucial determinant of redox potential,[91–97] and changes in this pocket can alter the redox potential by more than 400 mV.[90,98,99] Changes in the ligation and spin state of the heme iron also exert great influence upon the redox properties of hemoproteins, including those involved in electron transfer.[100–102]

Heme is a highly conjugated aromatic macrocycle that adopts a planar structure when free in solution. Despite this, high-resolution crystal structures and spectroscopic studies have shown that the heme-porphyrin structure often does not need to be planar when bound to a protein.[103–105] It has been demonstrated previously that the H-NOX protein from *Tt* H-NOX contains a highly distorted heme cofactor. Moreover, as the distortion from planarity decreased, the redox potential decreased by 171 mV.[105,106] Another study addressed whether a direct correlation exists between the degree of heme distortion and the redox potential.[107] They selected the I5L and I5L/P115A *Tt* H-NOX mutants as candidates for altering the porphyrin conformation. They demonstrated that the heme electronic properties can be modulated by porphyrin distortion within the same protein scaffold, without altering the heme ligation state or heme environment. The degree of heme distortion was found to be directly correlated to the electron density at the heme iron, as evidenced by dramatic changes in the heme redox potential and pK_a of the distal ligand (OH^- vs H_2O).[107] The absorption data indicated that planar hemes bind an axial water ligand in contrast to wild-type *Tt* H-NOX, which binds a hydroxide ligand. The UV-vis spectrum of wild-type *Tt* H-NOX is characteristic of a six-coordinate hydroxide-bound heme, while the spectrum of the ferric I5L mutant, which displays an intermediate degree of heme distortion, is characteristic of a mixture of both aqua- and hydroxide-bound heme.

The water ligand of H-NOX proteins can participate in acid-base reactions to generate a heme-bound OH^- ion (cf. **Scheme S10**). pH titrations of the ferric wild-type and mutant *Tt* H-NOX proteins were carried out, and the pK_a values for the distal bound water for wild-type and I5L *Tt* H-NOX were found to be 6.8 and 7.9, respectively.[107]

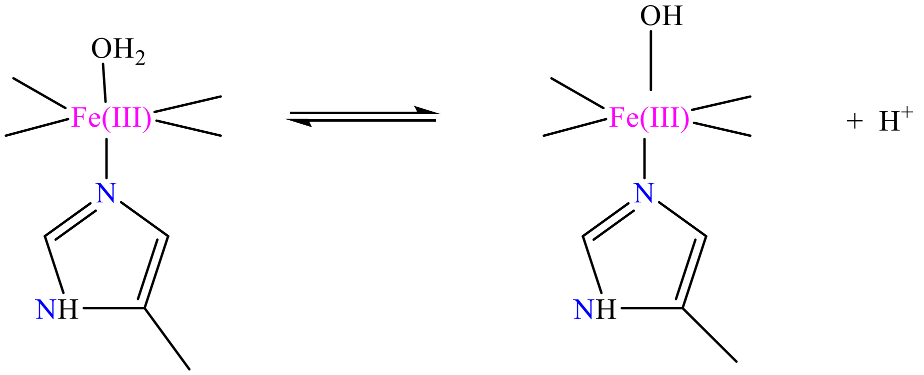

(11, 12)

Scheme S10. The acid-base reaction studied for H-NOX. Reactions 11 and 12 are the same but **occur in** different enzyme variants (WT and I5L, respectively).

2. QM Subsystem Definitions for ADH and CA

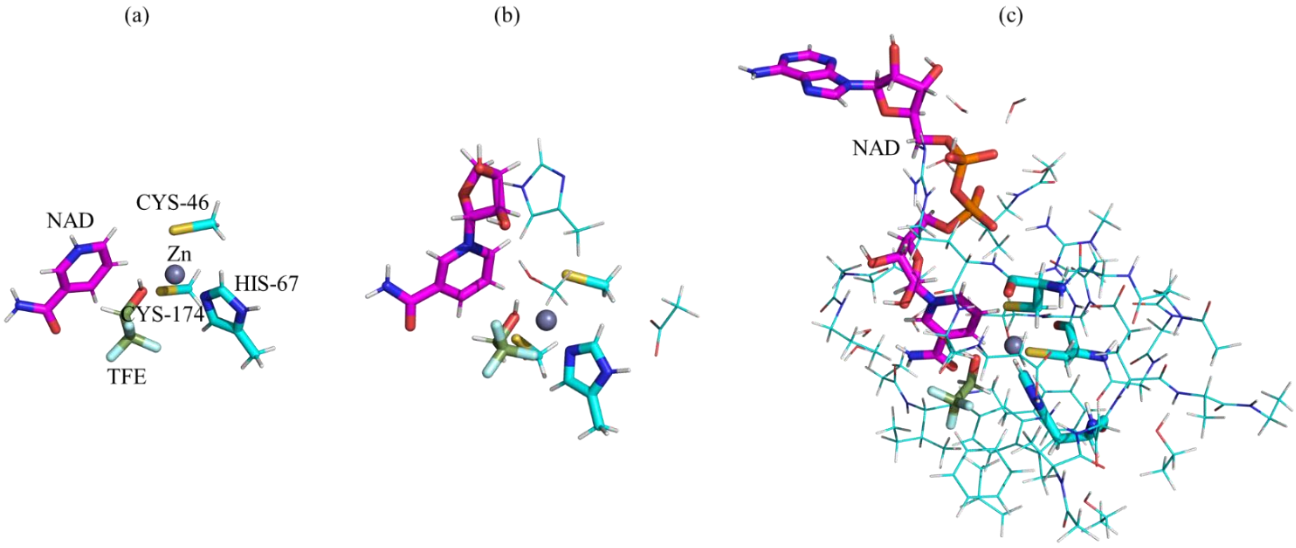

Figure S7. QM systems for the ADH-TFE system: (a) Min, (b) Int, and (c) Big. The minimal system is shown by sticks in the center of the Int and Big models.

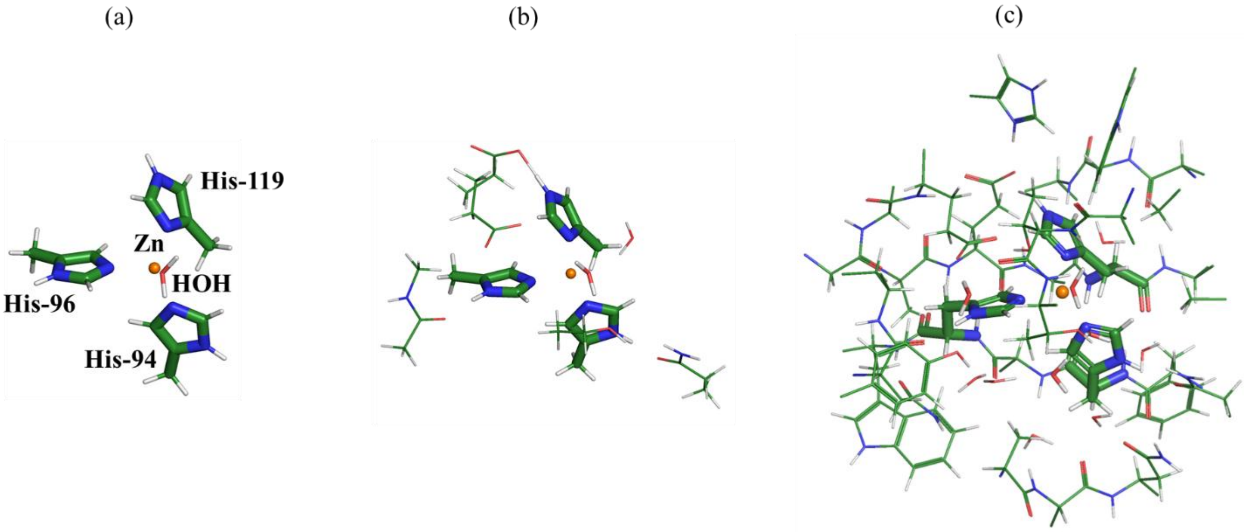

Figure S8. QM systems for CA-WT: (a) Min, (b) Int, and (c) Big. The minimal system is shown by sticks in the center of the Int and Big models.

3. Supplementary Tables

Table S1. Setup of the studied proteins, including the PDB structures used for calculations, QM and QM/MM system sizes (with HL atoms counted within the QM system), charge of the QM region in the AH state (that of the A^- state is one step more negative), and the protonation states of His residues (HID, HIE, and HIP correspond to protonation of ND1, NE2, and both ND1 and NE2, respectively; residue numbers are provided in the table). The residue numbers of neutral Glu (GLH) residues are also included. None of the structures contains any Cys–Cys cross-links or neutral Asp, Lys, or Arg residues.

Protein	State	PDB	Number of atoms				Charge of AH			Protonation state of His residues			GLH
		ID	QM/MM	Min	Int	Big	Min	Int	Big	HID	HIE	HIP	
ADH	TFE	1AXE	41360	48	86	485	1	0	0	67, 138, 139	51	34, 105, 348	267
	NAD^+	1AXE	41012	42	80	485	1	0	0	67, 138, 139	51	34, 105, 348	267
	NADH	1AXE	41068	43	81	481	0	-1	0	67, 138, 139	51	34, 105, 348	267
	Apo	8ADH	32810	26	51	229	0	-1	-1	51, 67, 138, 139		34, 105, 348	267
TLF	C55S	1M2B	12047	25	59	315	-2	-1	0	5, 17	89		
	C59S	1M2D	12161	25	62	311	-2	-1	0	5, 17	89		
CA	WT	2ILI	18939	40	103	357	2	1	2	3, 4, 10, 17, 36, 64, 94, 96, 122	15, 119	107	106
	E106Q	2ILI	18901	40	104	358	2	1	2	3, 4, 10, 17, 36, 64, 94, 96, 122	15, 119	107	
Mb	Cmp II	2V1E	12081	87	170	457	-1	0	0	24, 36, 48, 93, 113, 116	64, 97, 119	82	
P450	Cmp II	1S1F	32247	80	186	438	-2	1	1	351	57, 101, 255, 287, 343	85, 169, 172, 183, 405	362
H-NOX	WT	1U56	28376	88	175	489	-1	0	0	102			
	I5L	3NVR	18662	88	175	345	-1	0	-1	102			

Table S2. Calculated pK_a values for the various calculations on Mb and P450 based on either the triplet (from Table S5) or quintet state for compound II. The lower part of the table shows the energy difference (diff) between the quintet and triplet states (the latter is always more stable) in kJ/mol. Note that very large values reflect method artifacts and are not chemically interpretable as absolute pK_a values.

State	System	TPSS/def2-SV(P)						TPSS/def2-TZVPD						B3LYP/def2-SV(P)						B3LYP/def2-TZVPD					
	Surroundings	Fix			Free			Fix			Free			Fix			Free			Fix			Free		
	Eps	4	20	80	4	20	80	4	20	80	4	20	80	4	20	80	4	20	80	4	20	80	4	20	80
triplet	Mb	37.8	18.6	14.8	37.2	18.4	14.6	35.4	18.8	15.6	35.4	19.0	15.9	30.1	14.3	11.4	29.6	14.4	11.4	31.2	17.2	14.3	31.6	17.6	14.7
	P450	58.2	31.7	26.3	58.2	31.6	26.3	56.4	32.0	26.8	56.4	31.9	26.7	56.6	32.2	27.0	56.5	32.1	26.9	56.2	32.0	26.8	56.1	31.9	26.7
quintet	Mb	43.5	27.2	23.7	43.0	26.8	23.3	43.9	27.5	24.3	43.4	27.2	24.1	49.9	33.9	31.1	48.7	33.0	30.2	44.3	34.2	34.3	42.2	31.4	34.1
	P450	57.0	29.0	23.4	56.5	28.4	22.8	53.4	28.5	23.4	53.4	28.3	23.2	53.4	29.3	24.1	52.9	28.7	23.6	52.8	29.0	23.9	53.0	29.1	24.0
diff	Mb, AH	100	105	107	99	106	108	109	116	117	111	118	119	49	55	55	52	60	60	102	79	57	105	91	64
	Mb, A^-	133	154	158	132	154	158	158	166	167	157	165	166	163	167	168	162	167	168	177	176	173	166	170	176
	P450, AH	87	97	100	89	99	101	106	111	111	105	110	110	55	55	55	58	58	58	60	61	62	60	61	61
	P450, A^-	80	82	83	79	80	81	88	91	92	88	90	90	36	39	39	37	38	38	41	44	45	42	44	45

Table S3. ⟨S^2⟩ values for the various calculations with open-shell systems.

System	State	TPSS/def2-SV(P)						TPSS/def2-TZVPD						B3LYP/def2-SV(P)						B3LYP/def2-TZVPD					
Surroundings		Fix			Free			Fix			Free			Fix			Free			Fix			Free		
Eps		4	20	80	4	20	80	4	20	80	4	20	80	4	20	80	4	20	80	4	20	80	4	20	80
TLF-C55S	AH	3.81	3.86	3.87	3.63	3.69	3.71	4.45	4.46	4.46	4.43	4.44	4.44	3.83	3.89	3.89	3.71	3.77	3.78	4.41	4.43	4.43	4.41	4.42	4.42
	A^-	4.30	4.30	4.30	4.21	4.22	4.22	4.54	4.54	4.55	4.53	4.53	4.54	4.22	4.23	4.23	4.16	4.16	4.16	4.50	4.51	4.51	4.50	4.50	4.51
TLF-C59S	AH	4.20	4.21	4.22	4.04	4.05	4.05	4.51	4.52	4.52	4.43	4.45	4.45	4.12	4.13	4.13	4.05	4.06	4.07	4.48	4.48	4.49	4.47	4.48	4.48
	A^-	4.34	4.35	4.35	4.25	4.26	4.27	4.56	4.57	4.57	4.54	4.56	4.56	4.27	4.27	4.28	4.18	4.19	4.20	4.53	4.53	4.53	4.52	4.52	4.52
Mb	AH	2.02	2.02	2.02	2.02	2.02	2.02	2.03	2.04	2.04	2.05	2.05	2.05	2.02	2.02	2.02	2.02	2.02	2.02	2.03	2.04	2.04	2.04	2.04	2.04
	A^-	2.02	2.02	2.02	2.02	2.02	2.02	2.02	2.02	2.02	2.03	2.03	2.03	2.02	2.02	2.02	2.02	2.02	2.02	2.02	2.02	2.02	2.03	2.03	2.03
P450	AH	2.03	2.02	2.02	2.02	2.02	2.02	2.04	2.05	2.05	2.05	2.05	2.05	2.03	2.02	2.02	2.02	2.02	2.02	2.05	2.05	2.05	2.05	2.05	2.05
	A^-	2.02	2.02	2.02	2.02	2.02	2.02	2.03	2.03	2.03	2.03	2.03	2.03	2.02	2.02	2.02	2.02	2.02	2.02	2.03	2.03	2.03	2.04	2.03	2.03
HNOX-WT	AH	8.79	8.76	8.76	8.77	8.76	8.76	8.76	8.76	8.76	8.76	8.76	8.76	8.79	8.76	8.76	8.77	8.76	8.76	8.76	8.76	8.76	8.76	8.76	8.76
	A^-	8.76	8.76	8.76	8.76	8.76	8.76	8.76	8.76	8.76	8.76	8.76	8.76	8.76	8.76	8.76	8.76	8.76	8.76	8.76	8.76	8.76	8.76	8.76	8.76
HNOX-I5L	AH	8.78	8.76	8.76	8.77	8.76	8.76	8.76	8.76	8.76	8.76	8.76	8.76	8.79	8.76	8.76	8.77	8.76	8.76	8.77	8.76	8.76	8.76	8.76	8.76
	A^-	8.76	8.76	8.76	8.76	8.76	8.76	8.76	8.76	8.76	8.76	8.76	8.76	8.76	8.76	8.76	8.76	8.76	8.76	8.76	8.76	8.76	8.76	8.76	8.76

Table S4. Calculated pK_a values obtained with the QM/MM methods using the Min systems. Opt/SP indicates whether the results are based on optimized structures or on single-point calculations based on the corresponding TPSS/def2-SV(P) QM/MM structures. The DFT methods are TPSS (TP) or B3LYP (B3). The basis sets are def2-SV(P) (SV) or def2-TZVPD (TZ). The surroundings can either be relaxed (free) or fixed at the starting crystal structure. Note that very large values reflect method artifacts and are not chemically interpretable as absolute pK_a values.

Opt/SP	Optimized		Single Point					
DFT/basis	TP/SV		TP/TZ		B3/SV		B3/TZ	
Surroundings	Fix	Free	Fix	Free	Fix	Free	Fix	Free
ADH-TFE	-6.0	-4.5	-2.1	0.7	-1.3	2.0	-0.4	2.9
ADH-NAD	9.8	-3.5	18.1	11.7	15.8	8.7	19.5	13.0
ADH-NADH	46.7	13.1	42.6	14.1	50.8	17.1	44.0	14.6
ADH-Apo	37.4	7.9	33.9	-32.4	40.4	-28.9	34.0	-32.3
TLF-C55S	127.6	99.8	133.3	75.9	133.8	74.5	127.0	70.1
TLF-C59S	135.0	105.5	145.3	71.1	145.4	73.1	143.8	69.8
CA-WT	-1.6	-14.3	3.5	-18.4	4.7	-17.5	4.3	-19.0
CA-E106Q	1.4	-12.8	5.9	-17.5	7.4	-16.7	6.8	-18.1
Mb-CmpII	17.9	13.0	16.9	-1.1	13.5	-5.8	15.5	-2.2
P450-CmpII	99.8	89.5	97.7	68.5	99.5	69.5	97.9	68.4
HNOX-WT	6.5	-2.5	5.8	-9.5	6.0	-10.4	4.6	-10.6
HNOX-I5L	19.6	12.4	18.4	2.8	18.4	1.9	17.5	1.8

Table S5. Calculated pK_a values obtained with the QM + COSMO calculations using the Min systems. All methods involved single-point calculations with fixed surroundings. Note that very large values reflect method artifacts and are not chemically interpretable as absolute pK_a values.

DFT/basis	TP/SV			TP/TZ			B3/SV			B3/TZ		
Eps	4	20	80	4	20	80	4	20	80	4	20	80
ADH-TFE	4.8	10.4	11.6	7.4	12.7	13.8	6.6	12.2	13.3	8.5	13.7	14.7
ADH-NAD	11.4	18.7	20.2	5.4	12.8	14.4	16.0	23.3	24.9	9.4	16.8	18.4
ADH-NADH	39.4	30.7	28.8	35.9	26.8	24.8	35.8	27.4	25.5	32.8	23.8	21.8
ADH-Apo	41.6	32.6	29.4	43.2	32.4	33.6	36.8	27.8	25.9	37.3	26.5	24.1
TLF-C55S	63.6	29.8	22.7	62.9	29.3	22.2	60.9	27.9	20.9	59.8	26.9	19.9
TLF-C59S	66.3	33.0	26.0	64.1	31.1	24.1	69.0	36.2	29.2	60.7	28.5	21.7
CA-WT	-5.9	13.2	17.2	-6.9	11.6	15.5	-6.3	12.7	16.7	-8.0	10.3	14.1
CA-E106Q	-6.3	12.8	16.8	-7.5	11.0	14.9	-6.6	12.4	16.3	-8.6	9.6	13.5
Mb-CmpII	37.8	18.6	14.8	37.2	18.4	14.6	35.4	18.8	15.6	35.4	19.0	15.9
P450-CmpII	58.2	31.7	26.3	58.2	31.6	26.3	56.4	32.0	26.8	56.4	31.9	26.7
HNOX-WT	37.9	22.4	19.6	38.9	23.0	20.1	34.7	22.0	19.6	36.8	23.6	21.0
HNOX-I5L	38.4	22.7	19.8	38.7	22.4	19.4	35.6	22.7	20.3	36.5	22.9	20.3

Table S6. Calculated pK_a values obtained with the various methods using the Int systems. All calculations involved fixed surroundings. Note that very large values reflect method artifacts and are not chemically interpretable as absolute pK_a values.

Method	QM/MM				QM + COSMO											
Opt/SP	Opt	Single Point			Single Point											
DFT/basis	TP/SV	TP/TZ	B3/SV	B3/TZ	TP/SV			TP/TZ			B3/SV			B3/TZ		
Eps					4	20	80	4	20	80	4	20	80	4	20	80
ADH-TFE	-23.7	-24.9	-25.5	-24.8	9.8	8.1	7.8	11.8	10.0	9.7	10.4	8.8	8.5	12.1	10.5	10.2
ADH-NAD	9.1	11.7	12.2	12.6	27.1	24.4	23.9	29.5	26.9	26.4	28.0	25.4	24.9	30.3	27.7	27.2
ADH-NADH	19.1	10.7	10.7	11.2	35.1	22.0	19.2	36.4	23.4	20.7	35.7	22.6	19.9	36.8	23.9	21.3
ADH-Apo	45.5	39.7	46.8	39.6	61.9	42.4	38.3	56.4	37.1	32.9	62.3	42.7	38.6	56.0	36.6	32.5
TLF-C55S	97.8	91.3	100.8	90.9	32.4	18.2	15.4	33.4	19.0	16.1	28.4	15.2	12.4	28.5	15.3	12.6
TLF-C59S	115.6	114.8	115.7	117.0	43.8	27.3	23.9	46.7	28.8	25.0	42.8	26.6	23.2	45.9	27.8	23.9
CA-WT	-1.4	-5.3	-2.0	-6.1	13.5	17.5	18.3	9.9	13.8	14.6	12.8	16.8	17.6	8.9	12.7	13.5
CA-E106Q	0.8	-3.2	0.4	-4.1	16.1	19.6	20.3	12.3	15.7	16.4	15.6	19.0	19.7	11.3	14.6	15.3
Mb-CmpII	2.3	7.2	1.1	5.5	11.0	7.0	6.2	15.4	11.7	10.9	9.1	5.5	4.7	13.8	10.2	9.5
P450-CmpII	79.9	80.3	80.8	81.1	25.9	23.8	23.3	27.5	25.3	24.7	27.1	25.1	24.6	28.1	25.8	25.2
HNOX-WT	30.6	30.3	29.1	28.9	26.1	21.8	20.9	27.0	22.8	21.9	26.4	22.0	21.1	26.7	22.4	21.4
HNOX-I5L	29.3	32.4	28.9	31.6	17.8	14.1	13.3	20.9	17.2	16.4	18.5	14.8	14.0	21.2	17.5	16.7

Table S7. Calculated pK_a values obtained with the various methods using the Big systems. All calculations involved fixed surroundings. Note that very large values reflect method artifacts and are not chemically interpretable as absolute pK_a values.

Method	QM/MM				QM + COSMO											
Opt/SP	Opt	Single Point			Single Point											
DFT/basis	TP/SV	TP/TZ	B3/SV	B3/TZ	TP/SV			TP/TZ			B3/SV			B3/TZ		
Eps					4	20	80	4	20	80	4	20	80	4	20	80
ADH-TFE	16.1	13.7	16.4	14.4	34.3	30.3	29.4	33.2	29.3	28.4	35.4	31.4	30.5	34.0	30.0	29.1
ADH-NAD	14.5	23.0	20.3	25.2	25.0	21.8	21.1	29.8	26.7	26.0	26.8	23.6	23.0	31.7	28.6	28.0
ADH-NADH	6.0	1.7	6.4	1.2	19.9	17.6	17.2	16.3	14.2	13.7	20.1	17.8	17.3	15.8	13.6	13.2
ADH-Apo	12.4	14.8	17.5	16.1	32.8	21.6	19.3	32.5	21.4	19.0	34.3	23.2	20.8	33.5	22.4	20.1
TLF-C55S	86.1	93.9	86.8	94.9	20.2	17.8	17.3	26.1	23.4	22.8	20.0	17.7	17.2	27.2	24.4	23.8
TLF-C59S	57.3	73.2	52.5	37.4	-5.9	-6.1	-6.0	5.6	5.0	5.0	-14.6	-14.8	-14.8	-1.0	-1.9	-2.1
CA-WT	20.9	19.0	20.9	18.8	8.1	18.3	20.5	7.3	17.6	19.7	8.0	18.2	20.4	6.9	17.1	19.3
CA-E106Q	22.0	19.7	22.4	19.9	10.0	20.2	22.4	8.9	19.2	21.4	10.5	20.7	22.8	8.8	19.1	21.3
Mb-CmpII	34.3	32.7	33.3	30.4	15.8	11.7	10.9	17.0	13.7	13.0	17.5	13.7	12.9	15.7	12.3	11.6
P450-CmpII	95.5	97.2	98.9	98.4	20.1	21.4	21.6	18.2	19.8	20.1	20.8	22.1	22.3	19.4	20.8	21.0
HNOX-WT	48.2	41.6	49.2	40.0	33.0	30.5	29.9	24.2	21.7	21.2	32.8	30.3	29.8	23.4	21.0	20.5
HNOX-I5L	12.2	14.6	11.0	14.1	30.7	18.6	16.0	33.2	21.2	18.8	27.9	17.3	15.2	30.9	20.8	18.7

Table S8. Decomposition of QM/MM pK_a values into QM and MM contributions for the Min QM region (TPSS-D3BJ/def2-SV(P)), with fixed and relaxed MM surroundings. For each value, the total QM/MM pK_a value, as well as the QM ($\boldsymbol{E}^{\mathrm{HL}}_{\mathrm{QM1+ptch23}}$ in Eq. 1) and MM ($\boldsymbol{E}^{\mathrm{CL}}_{\mathrm{MM123},\boldsymbol{q}_1=0} - \boldsymbol{E}^{\mathrm{HL}}_{\mathrm{MM1},\boldsymbol{q}_1=0}$) components are given. The $\Delta G(\mathrm{H}^+)$ correction is assigned to the QM term. Values are given in pK_a units.

Protein	State	Fixed			Relaxed		
		QM/MM	QM	MM	QM/MM	QM	MM
ADH	TFE	-6.0	-3.3	-2.7	-4.5	-0.3	-4.2
	NAD	9.8	14.3	-4.5	-3.5	7.4	-10.9
	NADH	46.7	49.1	-2.4	13.1	16.4	-3.3
	Apo	37.4	39.6	-2.2	7.9	-29.4	37.3
TLF	C55S	127.6	138.8	-11.1	99.8	79.2	20.6
	C59S	135.0	146.7	-11.7	105.5	74.4	31.1
CA	WT	-1.6	3.5	-5.1	-14.3	-17.3	3.0
	E106Q	1.4	6.2	-4.8	-12.8	-16.4	3.7
Mb	Mb	17.9	15.9	2.0	13.0	-4.1	17.1
P450	P450	99.8	99.1	0.7	89.5	69.2	20.3
H-NOX	WT	6.5	6.8	-0.3	-2.5	-9.6	7.1
	I5L	19.6	19.3	0.3	12.4	2.7	9.7

Table S9. Thermostatistical corrections to the calculated pK_a values, calculated at the TPSS-D3BJ/def2-SV(P) level of theory.

System	Correction
ADH-TFE	-2.9
ADH-NAD	-5.0
ADH-NADH	-7.1
ADH-Apo	-5.8
TLF-C55S	-8.1
TLF-C59S	-6.7
CA-WT	-5.4
CA-E106Q	-5.4
Mb-CmpII	-5.2
P450-CmpII	-4.8
HNOX-WT	-7.2
HNOX-I5L	-6.2

Table S10. pK_a values for the 12 systems calculated from the Min/Fix/TPSS/def-SV(P) QM/MM structures for the QM-cluster models in continuum solvents described either with PB or GB (calculated with the Amber software, GB with the OBC, model II).[108] Both employ Merz–Kollman ESP charges from the QM calculations. The results are compared with the standard COSMO results obtained with Turbomole, as shown in Table 3. The lower part of the table contains the seven quality measures for each series.

System	PB			GB			COSMO		
Eps	4	20	80	4	20	80	4	20	80
ADH-TFE	6.7	11.3	12.2	8.4	13.3	14.2	4.8	10.4	11.6
ADH-NAD	14.5	20.3	21.4	16.1	22.3	23.5	11.4	18.7	20.2
ADH-NADH	38.4	32.3	31.1	38.8	33.2	32.2	39.4	30.7	28.8
ADH-Apo	39.8	33.3	32.1	39.2	32.8	31.6	41.6	32.6	29.4
TLF-C55S	48.7	22.1	17.1	48.0	21.0	16.0	63.6	29.8	22.7
TLF-C59S	51.3	24.9	19.9	50.3	23.5	18.5	66.3	33.0	26.0
CA-WT	4.4	20.3	23.4	6.7	23.1	26.2	-5.9	13.2	17.2
CA-E106Q	4.1	20.2	23.2	6.3	22.8	25.8	-6.3	12.8	16.8
Mb-CmpII	32.3	17.7	14.9	31.2	16.8	14.2	37.8	18.6	14.8
P450-CmpII	48.5	26.9	22.8	47.5	26.2	22.2	58.2	31.7	26.3
HNOX-WT	32.0	18.7	16.2	31.6	18.9	16.5	37.9	22.4	19.6
HNOX-I5L	33.8	21.0	18.5	32.9	20.2	17.8	38.4	22.7	19.8
trMAD	13.9	3.1	3.8	12.7	2.9	4.7	20.0	5.4	2.8
trMAX	24.6	8.4	8.6	22.6	8.9	9.2	37.7	9.4	6.3
Range	47.2	22.0	19.9	44.0	19.9	18.0	72.6	22.6	17.8
Slope	3.7	1.9	1.6	3.5	1.7	1.4	4.6	2.4	1.9
R^2	0.29	0.65	0.46	0.31	0.60	0.33	0.22	0.56	0.75
ρ	0.71	0.96	0.64	0.72	0.81	0.55	0.72	0.83	0.94
τ	0.48	0.85	0.45	0.52	0.61	0.39	0.52	0.67	0.79

Table S11. pK_a values for the 12 systems of the Min/Fix/TP/SV/80 models optimized either with COSMO or with QM/MM (the latter results from Table S5). The lower part of the table contains the seven quality measures for each series.

System	COSMO	QM/MM
ADH-TFE	13.7	11.6
ADH-NAD	12.6	20.2
ADH-NADH	27.7	28.8
ADH-Apo	26.6	29.4
TLF-C55S	26.6	22.7
TLF-C59S	23.5	26.0
CA-WT	13.9	17.2
CA-E106Q	14.2	16.8
Mb-CmpII	15.3	14.8
P450-CmpII	26.7	26.3
HNOX-WT	24.4	19.6
HNOX-I5L	12.3	19.8
trMAD	4.5	2.8
trMAX	7.7	6.3
Range	15.5	17.8
Slope	1.7	1.9
R^2	0.44	0.75
ρ	0.61	0.94
τ	0.39	0.79

Table S12. pK_a values for the 12 systems of the Min/Free/TP/SV models with an increased region of the QM system surroundings relaxed (10 instead of 6 Å; the latter results are also shown for reference, taken from Table S5). Results are presented after QM/MM optimization and after single-point QM-cluster calculations with ε = 4, 20, or 80. The lower part of the table contains the seven quality measures for each series.

	10 Å relaxed				6 Å relaxed			
		ε				ε		
	QM/MM	4	20	80	QM/MM	4	20	80
ADH-TFE	-2.6	6.3	11.9	13.0	-4.5	7.4	12.7	13.8
ADH-NAD	9.8	11.4	18.7	20.2	-3.5	5.4	12.8	14.4
ADH-NADH	46.7	39.4	30.7	28.8	13.1	35.9	26.8	24.8
ADH-Apo	37.4	41.6	32.6	30.7	7.9	43.2	32.4	33.6
TLF-C55S	127.6	63.6	29.9	22.7	99.8	62.9	29.3	22.2
TLF-C59S	135.0	66.3	33.0	26.0	105.5	64.1	31.1	24.1
CA-WT	-1.6	-5.9	13.2	17.2	-14.3	-6.9	11.6	15.5
CA-E106Q	1.4	-6.3	12.8	16.8	-12.8	-7.5	11.0	14.9
Mb-CmpII	18.0	37.8	18.7	14.8	13.0	37.2	18.4	14.6
P450-CmpII	99.8	58.2	31.6	26.3	89.5	58.2	31.6	26.3
HNOX-WT	6.5	37.9	22.3	19.6	-2.5	38.9	23.0	20.1
HNOX-I5L	19.6	38.7	22.7	19.9	12.4	38.7	22.4	19.4
trMAD	39.3	19.8	5.3	2.8	35.5	20.4	5.7	3.2
trMAX	93.0	37.9	9.5	6.0	79.7	38.1	10.1	9.8
Range	137.6	72.6	21.2	17.7	119.8	71.6	21.4	19.8
Slope	10.5	4.5	2.3	1.9	8.0	4.5	2.3	1.9
R^2	0.29	0.21	0.55	0.75	0.21	0.20	0.48	0.67
ρ	0.75	0.72	0.83	0.94	0.55	0.53	0.77	0.85
τ	0.52	0.52	0.67	0.79	0.33	0.36	0.58	0.70

Table S13. Calculated pK_a values for CA-WT based on ten snapshots extracted from a 100 ns MD simulation. pK_a values were computed using QM/MM (with def2-SV(P) and def2-TZVPD basis sets, denoted SV and TZ) and QM-cluster calculations with COSMO using dielectric constants $\varepsilon = 4$, 20, and 80 (TZ basis set). The lower part summarizes the mean, standard error (SE), and range across the ten snapshots, along with results obtained from the crystal structure.

Snapshot	SV	TZ	4	20	80
1	35.3	24.0	-7.7	10.8	14.7
2	13.8	10.1	-6.6	12.4	16.3
3	34.6	26.6	-6.7	12.7	16.8
4	16.2	5.0	-8.5	11.0	15.1
5	22.3	22.0	-9.7	9.3	13.3
6	14.5	7.0	-7.4	11.5	15.4
7	35.8	27.0	-6.7	12.5	16.5
8	24.8	13.2	-10.0	9.4	13.4
9	10.3	13.8	-6.1	13.2	17.2
10	40.4	19.3	-7.1	11.8	15.7
Av	24.8	16.8	-7.6	11.5	15.4
SE	3.5	2.6	0.4	0.4	0.4
Range	30.1	22.0	3.9	3.9	3.9
Crystal	-14.3	-15.5	-8.0	10.3	14.1

Table S14. pK_a values for the 12 systems of the Min/Fix/TP/SV/80 models recalculated with single-point QM+COSMO calculations with ε = 80 and four different DFT methods. The last column shows the results from the original TPSS calculations (from Table S5), and the lower part of the table contains the seven quality measures for each series, as well as three quality measures for absolute pK_a values.

	M06-L	M06	r^2SCAN	ωB97M-V	TPSS
ADH-TFE	10.3	10.3	9.0	10.6	11.6
ADH-NAD	20.4	18.6	18.2	16.4	20.2
ADH-NADH	28.3	28.1	27.2	27.4	28.8
ADH-Apo	29.4	29.6	29.3	29.0	29.4
TLF-C55S	18.1	17.5	18.2	15.2	22.7
TLF-C59S	23.3	23.6	23.0	21.5	26.0
CA-WT	16.5	15.8	16.1	15.3	17.2
CA-E106Q	16.2	15.5	15.8	14.9	16.8
Mb-CmpII	12.6	10.7	11.5	4.3	14.8
P450-CmpII	25.1	26.8	24.8	21.3	26.3
HNOX-WT	17.2	16.3	16.7	14.9	19.6
HNOX-I5L	18.3	17.1	17.3	15.4	19.8
trMAD	3.1	3.6	3.1	3.5	2.8
trMAX	7.2	7.5	6.9	8.7	6.3
Range	19.0	19.3	20.3	24.7	17.8
Slope	2.0	2.2	2.1	2.3	1.9
R^2	0.74	0.79	0.78	0.74	0.75
ρ	0.90	0.92	0.94	0.87	0.94
τ	0.73	0.76	0.79	0.76	0.79
MSE=MAD	11.9	11.4	11.2	9.5	13.4
Max	19.1	18.9	18.1	18.2	19.6

Table S15. Predicted pK_a values for CA-WT with His-64 in the outward (Out) conformation. Results for the inward (In) conformation from Tables S4 and S5 are included for comparison, along with the difference. All calculations were performed with the Min QM region and fixed surroundings.

Method	DFT	ε	Out	In	Diff
QM/MM	TP/SV		3.6	-1.6	5.1
	TP/TZ		8.5	3.5	5.0
	B3/SV		9.8	4.7	5.1
	B3/TZ		9.3	4.3	5.0
QM+COSMO	TP/SV	4	-6.3	-5.9	-0.3
		20	12.8	13.2	-0.3
		80	16.8	17.2	-0.4
	TP/TZ	4	-6.6	-6.3	-0.3
		20	12.4	12.7	-0.4
		80	16.3	16.7	-0.4
	B3/SV	4	-5.6	-5.3	-0.3
		20	13.5	13.8	-0.4
		80	17.5	17.8	-0.4
	B3/TZ	4	-6.3	-5.9	-0.4
		20	12.6	13.0	-0.4
		80	16.6	17.0	-0.4

4. References

(1) Pettersson, G.; Klinman, J. P. *Liver Alcohol Dehydrogenas*; 1986; Vol. 21. https://doi.org/10.3109/10409238609113616.

(2) KVASSMAN, J.; PETTERSSON, G. Effect of PH on the Binding of Decanoate and Trifluoroethanol to Liver Alcohol Dehydrogenase. *Eur. J. Biochem.* **1980**, *103* (3), 557–564. https://doi.org/10.1111/j.1432-1033.1980.tb05980.x.

(3) Brandon, C. I.; Jornvall, H.; Eklund, H.; Furugren, B. Alcohol Dehydrogenase in The Enzymes (Vol. XI, Part A Boyer, PD, Ed.). 1975.

(4) S. Cedergren-Zeppezauer, E.; Andersson, I.; Ottonello, S.; Bignetti, E. X-Ray Analysis of Structural Changes Induced by NADH When Bound to Cysteine-46-Carboxymethylated Liver Alcohol Dehydrogenase. *Biochemistry* **1985**, *24* (15), 4000–4010. https://doi.org/10.1021/bi00336a030.

(5) Argos, P.; Garavito, R. M.; Eventoff, W.; Rossmann, M. G.; Brändén, C. I. Similarities in Active Center Geometries of Zinc-Containing Enzymes, Proteases and Dehydrogenases. *J. Mol. Biol.* **1978**, *126* (2), 141–158. https://doi.org/10.1016/0022-2836(78)90356-X.

(6) Eklund, H.; Branden, C. I. Alcohol Dehydrogenase, in "Biological Macromolecules and Assemblies," Vol 2, FA Jurnak and A. McPherson, Eds. John Wiley \& Sons, New York 1987.

(7) Bahnson, B. J.; Colby, T. D.; Chin, J. K.; Goldstein, B. M.; Klinman, J. P. A Link between Protein Structure and Enzyme Catalyzed Hydrogen Tunneling. *Proc. Natl. Acad. Sci. U. S. A.* **1997**, *94* (24), 12797–12802. https://doi.org/10.1073/pnas.94.24.12797.

(8) Eklund, H.; Samama, J. P.; Alwyn Jones, T. Crystallographic Investigations of Nicotinamide Adenine Dinucleotide Binding to Horse Liver Alcohol Dehydrogenase. *Biochemistry* **1984**, *23* (25), 5982–5996. https://doi.org/10.1021/bi00320a014.

(9) KVASSMAN, J.; PETTERSSON, G. Effect of PH on Coenzyme Binding to Liver Alcohol Dehydrogenase. *Eur. J. Biochem.* **1979**, *100* (1), 115–123. https://doi.org/10.1111/j.1432-1033.1979.tb02039.x.

(10) DeTraglia, M. C.; Schmidt, J.; Dunn, M. F.; McFarland, J. T. Liver Alcohol Dehydrogenase Coenzyme Reaction Rates. *J. Biol. Chem.* **1977**, *252* (10), 3493–3500. https://doi.org/10.1016/s0021-9258(17)40418-2.

(11) Shore, J. D.; Gutfreund, H.; Brooks, R. L.; Santiago, D.; Santiago, P. Proton Equilibria and Kinetics in the Liver Alcohol Dehydrogenase Reaction Mechanism. *Biochemistry* **1974**, *13* (20), 4185–4191. https://doi.org/10.1021/bi00717a019.

(12) ANDERSSON, P.; KVASSMAN, J.; LINDSTRÖM, A.; OLDÉN, B.; PETTERSSON, G. Effect of NADH on the PKa of Zinc-Bound Water in Liver Alcohol Dehydrogenase. *Eur. J. Biochem.* **1981**, *113* (3), 425–433. https://doi.org/10.1111/j.1432-1033.1981.tb05082.x.

(13) Meyer, J. Miraculous Catch of Iron–Sulfur Protein Sequences in the Sargasso Sea. *FEBS Lett.* **2004**, *570* (1–3), 1–6. https://doi.org/10.1016/J.FEBSLET.2004.06.030.

(14) Hardy, R. W. F.; Knight Jr, E.; McDonald, C. C.; D'Eustachio, A. J. *Non-Heme Iron Proteins: Role in Energy Conversion*, 1st ed.; Antioch Press, 1965.

(15) Meyer, J.; Bruschi, M. H.; Bonicel, J. J.; Bovier-Lapierre, G. E. Amino Acid Sequence of [2Fe-2S] Ferredoxin from Clostridium Pasteurianum. *Biochemistry* **1986**, *25* (20), 6054–6061. https://doi.org/10.1021/BI00368A033.

(16) Meyer, J.; Moulis, J. M.; Lutz, M. Structural Differences between [2Fe-2S] Clusters in Spinach Ferredoxin and in the "Red Paramagnetic Protein" from Clostridium Pasteurianum. A Resonance Raman Study. *Biochem. Biophys. Res. Commun.* **1984**, *119* (3), 828–835. https://doi.org/10.1016/0006-291X(84)90848-9.

(17) Hinton, S. M.; Mortenson, L. E. Identification of Molybdoproteins in Clostridium Pasteurianum. *J. Bacteriol.* **1985**, *162* (2), 477–484. https://doi.org/10.1128/JB.162.2.477-484.1985.

(18) Meyer, J. Ferredoxins of the Third Kind. *FEBS Lett.* **2001**, *509* (1), 1–5. https://doi.org/10.1016/S0014-5793(01)03049-6.

(19) Deckert, G.; Warren, P. V.; Gaasterland, T.; Young, W. G.; Lenox, A. L.; Graham, D. E.; Overbeek, R.; Snead, M. A.; Keller, M.; Aujay, M.; Huber, R.; Feldman, R. A.; Short, J. M.; Olsen, G. J.; Swanson, R. V. The Complete Genome of the Hyperthermophilic Bacterium Aquifex Aeolicus. *Nature* **1998**, *392* (6674), 353–358. https://doi.org/10.1038/32831.

(20) Chatelet, C.; Gaillard, J.; Pétillot, Y.; Louwagie, M.; Meyer, J. A [2Fe–2S] Protein from the Hyperthermophilic Bacterium Aquifex Aeolicus. *Biochem. Biophys. Res. Commun.* **1999**, *261* (3), 885–889. https://doi.org/10.1006/BBRC.1999.1138.

(21) Moulis, J.-M.; Davasse, V. Probing the Role of Electrostatic Forces in the Interaction of Clostridium Pasteurianum Ferredoxin with Its Redox Partners. *Biochemistry* **2002**, *34* (51), 16781–16788. https://doi.org/10.1021/bi00051a028.

(22) Pan, G.; Menon, A. L.; Adams, M. W. W. Characterization of a [2Fe-2S] Protein Encoded in the Iron-Hydrogenase Operon of Thermotoga Maritima. *J. Biol. Inorg. Chem.* **2003**, *8* (4), 469–474. https://doi.org/10.1007/S00775-002-0439-Y.

(23) Chatelet, C.; Meyer, J. Mapping the Interaction of the [2Fe–2S] Clostridium Pasteurianum Ferredoxin with the Nitrogenase MoFe Protein. *Biochim. Biophys. Acta - Protein Struct. Mol. Enzymol.* **2001**, *1549* (1), 32–36. https://doi.org/10.1016/S0167-4838(01)00246-1.

(24) Golinelli, M. P.; Gagnon, J.; Meyer, J. Specific Interaction of the [2Fe-2S] Ferredoxin from Clostridium Pasteurianum with the Nitrogenase MoFe Protein. *Biochemistry* **1997**, *36* (39), 11797–11803. https://doi.org/10.1021/BI970528P.

(25) Sazanov, L. A.; Hinchliffe, P. Structure of the Hydrophilic Domain of Respiratory Complex I from Thermus Thermophilus. *Science* **2006**, *311* (5766), 1430–1436. https://doi.org/10.1126/SCIENCE.1123809.

(26) Vignais, P. M.; Billoud, B.; Meyer, J. Classification and Phylogeny of Hydrogenases. *FEMS Microbiol. Rev.* **2001**, *25* (4), 455–501. https://doi.org/10.1111/J.1574-6976.2001.TB00587.X.

(27) Chatelet, C.; Meyer, J. The [2Fe-2S] Protein I (Shetna Protein I) from Azotobacter Vinelandii Is Homologous to the [2Fe-2S] Ferredoxin from Clostridium Pasteurianum. *JBIC J. Biol. Inorg. Chem.* **1999**, *4* (3), 311–317.

(28) Meyer, J. Iron-Sulfur Protein Folds, Iron-Sulfur Chemistry, and Evolution. *J. Biol. Inorg. Chem.* **2008**, *13* (2), 157–170. https://doi.org/10.1007/S00775-007-0318-7.

(29) Yeh, A. P.; Chatelet, C.; Soltis, S. M.; Kuhn, P.; Meyer, J.; Rees, D. C. Structure of a

Thioredoxin-like [2Fe-2S] Ferredoxin from Aquifex Aeolicus. *J. Mol. Biol.* **2000**, *300* (3), 587–595. https://doi.org/10.1006/JMBI.2000.3871.

(30) Holmgren, A.; Soderberg, B. O.; Eklund, H.; Branden, C. I. Three-Dimensional Structure of Escherichia Coli Thioredoxin-S2 to 2.8 A Resolution. *Proc. Natl. Acad. Sci.* **1975**, *72* (6), 2305–2309. https://doi.org/10.1073/PNAS.72.6.2305.

(31) Paddock, M. L.; Wiley, S. E.; Axelrod, H. L.; Cohen, A. E.; Roy, M.; Abresch, E. C.; Capraro, D.; Murphy, A. N.; Nechushtai, R.; Dixon, J. E.; Jennings, P. A. MitoNEET Is a Uniquely Folded 2Fe-2S Outer Mitochondrial Membrane Protein Stabilized by Pioglitazone. *Proc. Natl. Acad. Sci. U. S. A.* **2007**, *104* (36), 14342–14347. https://doi.org/10.1073/PNAS.0707189104/SUPPL_FILE/07189FIG6.JPG.

(32) Colbert, C. L.; Couture, M. M. J.; Eltis, L. D.; Bolin, J. T. A Cluster Exposed: Structure of the Rieske Ferredoxin from Biphenyl Dioxygenase and the Redox Properties of Rieske Fe-S Proteins. *Structure* **2000**, *8* (12), 1267–1278. https://doi.org/10.1016/S0969-2126(00)00536-0.

(33) Bönisch, H.; Schmidt, C. L.; Schäfer, G.; Ladenstein, R. The Structure of the Soluble Domain of an Archaeal Rieske Iron–Sulfur Protein at 1.1 Å Resolution. *J. Mol. Biol.* **2002**, *319* (3), 791–805. https://doi.org/10.1016/S0022-2836(02)00323-6.

(34) Iwata, S.; Saynovits, M.; Link, T. A.; Michel, H. Structure of a Water Soluble Fragment of the "Rieske" Iron-Sulfur Protein of the Bovine Heart Mitochondrial Cytochrome Bc1 Complex Determined by MAD Phasing at 1.5 Å Resolution. *Structure* **1996**, *4* (5), 567–579. https://doi.org/10.1016/S0969-2126(96)00062-7.

(35) Berkovitch, F.; Nicolet, Y.; Wan, J. T.; Jarrett, J. T.; Drennan, C. L. Crystal Structure of Biotin Synthase, an S-Adenosylmethionine-Dependent Radical Enzyme. *Science (80-.).* **2004**, *303* (5654), 76–79. https://doi.org/10.1126/SCIENCE.1088493/SUPPL_FILE/BERKOVITCH.SOM.PDF.

(36) Yeh, A. P.; Ambroggio, X. I.; Andrade, S. L. A.; Einsle, O.; Chatelet, C.; Meyer, J.; Rees, D. C. High Resolution Crystal Structures of the Wild Type and Cys-55 → Ser and Cys-59 → Ser Variants of the Thioredoxin-like [2Fe-2S] Ferredoxin from Aquifex Aeolicus. *J. Biol. Chem.* **2002**, *277* (37), 34499–34507. https://doi.org/10.1074/jbc.M205096200.

(37) Subramanian, S.; C. Duin, E.; E. J. Fawcett, S.; A. Armstrong, F.; Meyer, J.; K. Johnson, M. Spectroscopic and Redox Studies of Valence-Delocalized [Fe2S2]+ Centers in Thioredoxin-like Ferredoxins. *J. Am. Chem. Soc.* **2015**, *137* (13), 4567–4580. https://doi.org/10.1021/jacs.5b01869.

(38) STEINER, H.; JONSSON, B.-H.; LINDSKOG, S. The Catalytic Mechanism of Carbonic Anhydrase. Hydrogen-Isotope Effects on the Kinetic Parameters of the Human C Isoenzyme. *Eur. J. Biochem.* **1975**, *59* (1), 253–259. https://doi.org/10.1111/j.1432-1033.1975.tb02449.x.

(39) Pocker, Y.; Meany, J. E. The Catalytic Versatility of Carbonic Anhydrase from Erythrocytes. The Enzyme-Catalyzed Hydration of Acetaldehyde. *J. Am. Chem. Soc.* **1965**, *87* (8), 1809–1811.

(40) Tashian, R. E.; Plato, C. C.; Shows Jr, T. B. Inherited Variant of Erythrocyte Carbonic Anhydrase in Micronesians from Guam and Saipan. *Science (80-.).* **1963**, *140* (3562), 53–54.

(41) Keilin, D.; Mann, T. Carbonic Anhydrase. Purification and Nature of the Enzyme. *Biochem. J.* **1940**, *34* (8–9), 1163.

(42) Henderson, L. E.; Henriksson, D.; Nyman, P. O. Primary Structure of Human Carbonic

Anhydrase C. *J. Biol. Chem.* **1976**, *251* (18), 5457–5463.

(43) Lindskog, S.; Malmström, B. G. Metal Binding and Catalytic Activity in Bovine Carbonic Anhydrase. *J. Biol. Chem.* **1962**, *237* (4), 1129–1137.

(44) Eriksson, A. E.; Jones, T. A.; Liljas, A. Refined Structure of Human Carbonic Anhydrase II at 2.0 Å Resolution. *Proteins Struct. Funct. Bioinforma.* **1988**, *4* (4), 274–282. https://doi.org/10.1002/PROT.340040406.

(45) Håkansson, K.; Carlsson, M.; Svensson, L. A.; Liljas, A. Structure of Native and Apo Carbonic Anhydrase II and Structure of Some of Its Anion-Ligand Complexes. *J. Mol. Biol.* **1992**, *227* (4), 1192–1204. https://doi.org/10.1016/0022-2836(92)90531-N.

(46) Fisher, S. Z.; Maupin, C. M.; Budayova-Spano, M.; Govindasamy, L.; Tu, C.; Agbandje-McKenna, M.; Silverman, D. N.; Voth, G. A.; McKenna, R. Atomic Crystal and Molecular Dynamics Simulation Structures of Human Carbonic Anhydrase II: Insights into the Proton Transfer Mechanism. *Biochemistry* **2007**, *46* (11), 2930–2937. https://doi.org/10.1021/bi062066y.

(47) Silverman, D. N.; Lindskog, S. The Catalytic Mechanism of Carbonic Anhydrase: Implications of a Rate-Limiting Protolysis of Water. *Acc. Chem. Res.* **1988**, *21* (1), 30–36. https://doi.org/10.1021/ar00145a005.

(48) W. Christianson, D.; A. Fierke, C. Carbonic Anhydrase: Evolution of the Zinc Binding Site by Nature and by Design. *Acc. Chem. Res.* **1996**, *29* (7), 331–339. https://doi.org/10.1021/ar9501232.

(49) Lindskog, S. Structure and Mechanism of Carbonic Anhydrase. *Pharmacol. Ther.* **1997**, *74* (1), 1–20. https://doi.org/10.1016/S0163-7258(96)00198-2.

(50) Lesburg, C. A.; Christianson, D. W. X-Ray Crystallographic Studies of Engineered Hydrogen Bond Networks in a Protein–Zinc Binding Site. *J. Am. Chem. Soc.* **1995**, *117* (26), 6838–6844. https://doi.org/10.1021/ja00131a005.

(51) Nair, S. K.; Christianson, D. W. Unexpected PH-Dependent Conformation of His-64, the Proton Shuttle of Carbonic Anhydrase II. *J. Am. Chem. Soc.* **1991**, *113* (25), 9455–9458. https://doi.org/10.1021/ja00025a005.

(52) Fisher, Z.; A. Hernandez Prada, J.; Tu, C.; Duda, D.; Yoshioka, C.; An, H.; Govindasamy, L.; N. Silverman, D.; McKenna, R. Structural and Kinetic Characterization of Active-Site Histidine as a Proton Shuttle in Catalysis by Human Carbonic Anhydrase II,. *Biochemistry* **2005**, *44* (4), 1097–1105. https://doi.org/10.1021/bi0480279.

(53) Tu, C.; Silverman, D. N.; Forsman, C.; Jonsson, B. H.; Lindskog, S. Role of Histidine 64 in the Catalytic Mechanism of Human Carbonic Anhydrase II Studied with a Site-Specific Mutant. *Biochemistry* **1989**, *28* (19), 7913–7918. https://doi.org/10.1021/bi00445a054.

(54) Forsman, C.; Behravan, G.; Jonsson, B. H.; Liang, Z. wei; Lindskog, S.; Ren, X.; Sandström, J.; Wallgren, K. Histidine 64 Is Not Required for High CO2 Hydration Activity of Human Carbonic Anhydrase II. *FEBS Lett.* **1988**, *229* (2), 360–362. https://doi.org/10.1016/0014-5793(88)81156-6.

(55) Åqvist, J.; Warshel, A. Computer Simulation of the Initial Proton Transfer Step in Human Carbonic Anhydrase I. *J. Mol. Biol.* **1992**, *224* (1), 7–14. https://doi.org/10.1016/0022-2836(92)90572-2.

(56) Pocker, Y.; Sarkanen, S. Carbonic Anhydrase: Structure Catalytic Versatility, and Inhibition. *Adv. Enzymol. Relat. Areas Mol. Biol.* **1978**, *47*, 149–274. https://doi.org/10.1002/9780470122921.CH3.

(57) Silverman, D. N.; Vincent, S. H. Proton Transfer in the Catalytic Mechanism of Carbonic Anhydras. *Crit. Rev. Biochem. Mol. Biol.* **1983**, *14* (3), 207–255. https://doi.org/10.3109/10409238309102794.

(58) Nilsson, K.; Hersleth, H. P.; Rod, T. H.; Andersson, K. K.; Ryde, U. The Protonation Status of Compound II in Myoglobin, Studied by a Combination of Experimental Data and Quantum Chemical Calculations: Quantum Refinement. *Biophys. J.* **2004**, *87* (5), 3437–3447. https://doi.org/10.1529/biophysj.104.041590.

(59) Vanek, T.; Kohli, A. Biochemistry, Myoglobin. **2019**.

(60) Spiro, T. G.; Kozlowski, P. M. Is the CO Adduct of Myoglobin Bent, and Does It Matter? *Acc. Chem. Res.* **2001**, *34* (2), 137–144. https://doi.org/10.1021/ar000108j.

(61) Hersleth, H. P.; Uchida, T.; Røhr, Å. K.; Teschner, T.; Schünemann, V.; Kitagawa, T.; Trautwein, A. X.; Görbitz, C. H.; Andersson, K. K. Crystallographic and Spectroscopic Studies of Peroxide-Derived Myoglobin Compound II and Occurrence of Protonated FeIV–O *. *J. Biol. Chem.* **2007**, *282* (32), 23372–23386. https://doi.org/10.1074/JBC.M701948200.

(62) Yosca, T. H.; Behan, R. K.; Krest, C. M.; Onderko, E. L.; Langston, M. C.; Green, M. T. Setting an Upper Limit on the Myoglobin Iron(IV)Hydroxide p K a: Insight into Axial Ligand Tuning in Heme Protein Catalysis. *J. Am. Chem. Soc.* **2014**, *136* (25), 9124–9131. https://doi.org/10.1021/ja503588n.

(63) P.B. Danielson, B. S. P. The Cytochrome P450 Superfamily: Biochemistry, Evolution and Drug Metabolism in Humans. *Curr. Drug Metab.* **2002**, *3* (6), 561–597. https://doi.org/10.2174/1389200023337054.

(64) Hasemann, C. A.; Kurumbail, R. G.; Boddupalli, S. S.; Peterson, J. A.; Deisenhofer, J. Structure and Function of Cytochromes P450:A Comparative Analysis of Three Crystal Structures. *Structure* **1995**, *3* (1), 41–62. https://doi.org/10.1016/S0969-2126(01)00134-4.

(65) Denisov, I. G.; Makris, T. M.; Sligar, S. G.; Schlichting, I. Structure and Chemistry of Cytochrome P450. *Chem. Rev.* **2005**, *105* (6), 2253–2277. https://doi.org/10.1021/CR0307143/ASSET/CR0307143.FP.PNG_V03.

(66) Presnell, S. R.; Cohen, F. E. Topological Distribution of Four-Alpha-Helix Bundles. *Proc. Natl. Acad. Sci. U. S. A.* **1989**, *86* (17), 6592–6596. https://doi.org/10.1073/pnas.86.17.6592.

(67) Zhao, B.; Guengerich, F. P.; Bellamine, A.; Lamb, D. C.; Izumikawa, M.; Lei, L.; Podust, L. M.; Sundaramoorthy, M.; Kalaitzis, J. A.; Reddy, L. M.; Kelly, S. L.; Moore, B. S.; Stec, D.; Voehler, M.; Falck, J. R.; Shimada, T.; Waterman, M. R. Binding of Two Flaviolin Substrate Molecules, Oxidative Coupling, and Crystal Structure of Streptomyces Coelicolor A3(2) Cytochrome P450 158A2. *J. Biol. Chem.* **2005**, *280* (12), 11599–11607. https://doi.org/10.1074/jbc.M410933200.

(68) De Montellano, P. R. O.; others. *Cytochrome P450: Structure, Mechanism, and Biochemistry*; Springer, 2005; Vol. 3.

(69) Ortiz De Montellano, P. R. Hydrocarbon Hydroxylation by Cytochrome P450 Enzymes. *Chem. Rev.* **2010**, *110* (2), 932–948. https://doi.org/10.1021/CR9002193/ASSET/CR9002193.FP.PNG_V03.

(70) Kellner, D. G.; Hung, S. C.; Weiss, K. E.; Sligar, S. G. Kinetic Characterization of Compound I Formation in the Thermostable Cytochrome P450 CYP119. *J. Biol. Chem.* **2002**, *277* (12), 9641–9644. https://doi.org/10.1074/jbc.C100745200.

(71) Egawa, T.; Shimada, H.; Ishimura, Y. Evidence for Compound i Formation in the Reaction of Cytochrome-P450cam with m-Chloroperbenzoic Acid. *Biochemical and Biophysical Research Communications*. 1994, pp 1464–1469. https://doi.org/10.1006/bbrc.1994.1868.

(72) Rittle, J.; Green, M. T. Cytochrome P450 Compound I: Capture, Characterization, and C-H Bond Activation Kinetics. *Science (80-.).* **2010**, *330* (6006), 933–937. https://doi.org/10.1126/SCIENCE.1193478.

(73) Krest, C. M.; Onderko, E. L.; Yosca, T. H.; Calixto, J. C.; Karp, R. F.; Livada, J.; Rittle, J.; Green, M. T. Reactive Intermediates in Cytochrome P450 Catalysis. *J. Biol. Chem.* **2013**, *288* (24), 17074–17081. https://doi.org/10.1074/jbc.R113.473108.

(74) Yosca, T. H.; Rittle, J.; Krest, C. M.; Onderko, E. L.; Silakov, A.; Calixto, J. C.; Behan, R. K.; Green, M. T. Iron(IV)Hydroxide PKa and the Role of Thiolate Ligation in C-H Bond Activation by Cytochrome P450. *Science (80-.).* **2013**, *342* (6160), 825–829. https://doi.org/10.1126/science.1244373.

(75) Lawson, D. M.; Stevenson, C. E. M.; Andrew, C. R.; Eady, R. R. Unprecedented Proximal Binding of Nitric Oxide to Heme: Implications for Guanylate Cyclase. *EMBO J.* **2000**, *19* (21), 5661–5671. https://doi.org/10.1093/EMBOJ/19.21.5661.

(76) S. Karow, D.; Pan, D.; Tran, R.; Pellicena, P.; Presley, A.; A. Mathies, R.; A. Marletta, M. Spectroscopic Characterization of the Soluble Guanylate Cyclase-like Heme Domains from Vibrio Cholerae and Thermoanaerobacter Tengcongensis. *Biochemistry* **2004**, *43* (31), 10203–10211. https://doi.org/10.1021/bi049374l.

(77) Hao, B.; Isaza, C.; Arndt, J.; Soltis, M.; K. Chan, M. Structure-Based Mechanism of O2 Sensing and Ligand Discrimination by the FixL Heme Domain of Bradyrhizobium Japonicum,. *Biochemistry* **2002**, *41* (43), 12952–12958. https://doi.org/10.1021/bi020144l.

(78) Sykes, A. G.; Mauk, G. *Heme-Fe Proteins*; Elsevier, 2000.

(79) Reedy, C. J.; Elvekrog, M. M.; Gibney, B. R. Development of a Heme Protein Structure–Electrochemical Function Database. *Nucleic Acids Res.* **2008**, *36* (Database issue), D307. https://doi.org/10.1093/NAR/GKM814.

(80) Boon, E. M.; Davis, J. H.; Tran, R.; Karow, D. S.; Huang, S. H.; Pan, D.; Miazgowicz, M. M.; Mathies, R. A.; Marletta, M. A. Nitric Oxide Binding to Prokaryotic Homologs of the Soluble Guanylate Cyclase B1 H-NOX Domain. *J. Biol. Chem.* **2006**, *281* (31), 21892–21902. https://doi.org/10.1074/jbc.M600557200.

(81) Liao, M.-S.; Huang, M.-J.; D. Watts, J. Binding of O2 and NO to Heme in Heme-Nitric Oxide/Oxygen-Binding (H-NOX) Proteins. A Theoretical Study. *J. Phys. Chem. B* **2013**, *117* (35), 10103–10114. https://doi.org/10.1021/jp403998u.

(82) Toda, N.; Okamura, T. The Pharmacology of Nitric Oxide in the Peripheral Nervous System of Blood Vessels. *Pharmacol. Rev.* **2003**, *55* (2), 271–324. https://doi.org/10.1124/pr.55.2.3.

(83) Friedman, J.; Meharenna, Y. T.; Wilks, A.; Poulos, T. L. Diatomic Ligand Discrimination by the Heme Oxygenases from Neisseria Meningitidis and Pseudomonas Aeruginosa. *J. Biol. Chem.* **2007**, *282* (2), 1066–1071. https://doi.org/10.1074/jbc.M609112200.

(84) Boon, E. M.; Marletta, M. A. Ligand Discrimination in Soluble Guanylate Cyclase and the H-NOX Family of Heme Sensor Proteins. *Curr. Opin. Chem. Biol.* **2005**, *9* (5), 441–446. https://doi.org/10.1016/j.cbpa.2005.08.015.

(85) Boon, E. M.; Marletta, M. A. Ligand Specificity of H-NOX Domains: From SGC to Bacterial NO Sensors. *J. Inorg. Biochem.* **2005**, *99* (4), 892–902. https://doi.org/10.1016/j.jinorgbio.2004.12.016.

(86) Boon, E. M.; Huang, S. H.; Marletta, M. A. A Molecular Basis for No Selectivity in Soluble Guanylate Cyclase. *Nat. Chem. Biol.* **2005**, *1* (1), 53–59. https://doi.org/10.1038/nchembio704.

(87) Nioche, P.; Berka, V.; Vipond, J.; Minton, N.; Tsai, A. L.; Raman, C. S. Femtomolar Sensitivity of a NO Sensor from Clostridium Botulinum. *Science (80-.).* **2004**, *306* (5701), 1550–1553. https://doi.org/10.1126/SCIENCE.1103596/SUPPL_FILE/NIOCHE.SOM.PDF.

(88) Pellicena, P.; Karow, D. S.; Boon, E. M.; Marletta, M. A.; Kuriyan, J. Crystal Structure of an Oxygen-Binding Heme Domain Related to Soluble Guanylate Cyclases. *Proc. Natl. Acad. Sci. U. S. A.* **2004**, *101* (35), 12854–12859. https://doi.org/10.1073/PNAS.0405188101/SUPPL_FILE/05188FIG7.JPG.

(89) Paoli, M.; Marles-Wright, J.; Smith, A. Structure-Function Relationships in Heme-Proteins. *DNA Cell Biol.* **2002**, *21* (4), 271–280. https://doi.org/10.1089/104454902753759690.

(90) M. Shifman, J.; R. Gibney, B.; Eryl Sharp, R.; Leslie Dutton, P. Heme Redox Potential Control in de Novo Designed Four-α-Helix Bundle Proteins. *Biochemistry* **2000**, *39* (48), 14813–14821. https://doi.org/10.1021/bi000927b.

(91) Mao, J.; Hauser, K.; R. Gunner, M. How Cytochromes with Different Folds Control Heme Redox Potentials. *Biochemistry* **2003**, *42* (33), 9829–9840. https://doi.org/10.1021/bi027288k.

(92) Pascher, T.; Chesick, J. P.; Winkler, J. R.; Gray, H. B. Protein Folding Triggered by Electron Transfer. *Science (80-.).* **1996**, *271* (5255), 1558–1560. https://doi.org/10.1126/SCIENCE.271.5255.1558.

(93) Bertrand, P.; Mbarki, O.; Asso, M.; Blanchard, L.; Guerlesquin, F.; Tegoni, M. Control of the Redox Potential in C-Type Cytochromes: Importance of the Entropic Contribution. *Biochemistry* **2002**, *34* (35), 11071–11079. https://doi.org/10.1021/bi00035a012.

(94) Gunner, M. R.; Honig, B. Electrostatic Control of Midpoint Potentials in the Cytochrome Subunit of the Rhodopseudomonas Viridis Reaction Center. *Proc. Natl. Acad. Sci.* **1991**, *88* (20), 9151–9155. https://doi.org/10.1073/PNAS.88.20.9151.

(95) Rivera, M.; Seetharaman, R.; Girdhar, D.; Wirtz, M.; Zhang, X.; Wang, X.; White, S. The Reduction Potential of Cytochrome B5 Is Modulated by Its Exposed Heme Edge. *Biochemistry* **1998**, *37* (6), 1485–1494. https://doi.org/10.1021/bi972390g.

(96) Mauk, A. G.; Moore, G. R. Control of Metalloprotein Redox Potentials: What Does Site-Directed Mutagenesis of Hemoproteins Tell Us? *J. Biol. Inorg. Chem.* **1997**, *2* (1), 119–125. https://doi.org/10.1007/S007750050115/METRICS.

(97) Varadarajan, R.; Zewert, T. E.; Gray, H. B.; Boxer, S. G. Effects of Buried Ionizable Amino Acids on the Reduction Potential of Recombinant Myoglobin. *Science (80-.).* **1989**, *243* (4887), 69–72. https://doi.org/10.1126/SCIENCE.2563171.

(98) B. Cowley, A.; L. Kennedy, M.; Silchenko, S.; S. Lukat-Rodgers, G.; R. Rodgers, K.; R. Benson,

D. Insight into Heme Protein Redox Potential Control and Functional Aspects of Six-Coordinate Ligand-Sensing Heme Proteins from Studies of Synthetic Heme Peptides. *Inorg. Chem.* **2006**, *45* (25), 9985–10001. https://doi.org/10.1021/ic052205k.

(99) Kennedy, M. L.; Gibney, B. R. Metalloprotein and Redox Protein Design. *Curr. Opin. Struct. Biol.* **2001**, *11* (4), 485–490. https://doi.org/10.1016/S0959-440X(00)00237-2.

(100) Battistuzzi, G.; Borsari, M.; A. Cowan, J.; Ranieri, A.; Sola, M. Control of Cytochrome c Redox Potential: Axial Ligation and Protein Environment Effects. *J. Am. Chem. Soc.* **2002**, *124* (19), 5315–5324. https://doi.org/10.1021/ja017479v.

(101) Akif Tezcan, F.; R. Winkler, J.; B. Gray, H. Effects of Ligation and Folding on Reduction Potentials of Heme Proteins. *J. Am. Chem. Soc.* **1998**, *120* (51), 13383–13388. https://doi.org/10.1021/ja982536e.

(102) T. Fisher, M.; G. Sligar, S. Control of Heme Protein Redox Potential and Reduction Rate: Linear Free Energy Relation between Potential and Ferric Spin State Equilibrium. *J. Am. Chem. Soc.* **2002**, *107* (17), 5018–5019. https://doi.org/10.1021/ja00303a045.

(103) Jentzen, W.; Ma, J. G.; Shelnutt, J. A. Conservation of the Conformation of the Porphyrin Macrocycle in Hemoproteins. *Biophys. J.* **1998**, *74* (2), 753–763. https://doi.org/10.1016/S0006-3495(98)74000-7.

(104) Shelnutt, J. A.; Song, X. Z.; Ma, J. G.; Jia, S. L.; Jentzen, W.; Medforth, C. J. Nonplanar Porphyrins and Their Significance in Proteins. *Chem. Soc. Rev.* **1998**, *27* (1), 31–42. https://doi.org/10.1039/A827031Z.

(105) Olea Jr., C.; M. Boon, E.; Pellicena, P.; Kuriyan, J.; A. Marletta, M. Probing the Function of Heme Distortion in the H-NOX Family. *ACS Chem. Biol.* **2008**, *3* (11), 703–710. https://doi.org/10.1021/cb800185h.

(106) Tran, R.; M. Boon, E.; A. Marletta, M.; A. Mathies, R. Resonance Raman Spectra of an O2-Binding H-NOX Domain Reveal Heme Relaxation upon Mutation. *Biochemistry* **2009**, *48* (36), 8568–8577. https://doi.org/10.1021/bi900563g.

(107) Olea, C.; Kuriyan, J.; Marletta, M. A. Modulating Heme Redox Potential through Protein-Induced Porphyrin Distortion. *J. Am. Chem. Soc.* **2010**, *132* (37), 12794–12795. https://doi.org/10.1021/ja106252b.

(108) Onufriev, A.; Bashford, D.; Case, D. A. Exploring Protein Native States and Large-Scale Conformational Changes with a Modified Generalized Born Model. *Proteins Struct. Funct. Bioinforma.* **2004**, *55* (2), 383–394. https://doi.org/10.1002/prot.20033.
